# RNA-seq of the aging brain in the short-lived fish *N. furzeri* – conserved pathways and novel genes associated with neurogenesis

**DOI:** 10.1111/acel.12257

**Published:** 2014-07-25

**Authors:** Mario Baumgart, Marco Groth, Steffen Priebe, Aurora Savino, Giovanna Testa, Andreas Dix, Roberto Ripa, Francesco Spallotta, Carlo Gaetano, Michela Ori, Eva Terzibasi Tozzini, Reinhard Guthke, Matthias Platzer, Alessandro Cellerino

**Affiliations:** 1 Leibniz Institute for Age Research - Fritz Lipmann Institute e.V. (FLI)Jena, Germany; 2 Leibniz Institute for Natural Product Research and Infection Biology - Hans-Knöll-Institute e.V. (HKI)Jena, Germany; 3 Laboratory of NeuroBiology, Scuola Normale Superiore, University of PisaPisa, Italy; 4 Department of Biology, University of PisaPisa, Italy; 5 Klinikum der Johann Wolfgang Goethe-UniversitätFrankfurt am Main, Germany

**Keywords:** animal model, brain aging, epigenetics, gene expression, neural stem cells, neurogenesis, teleost, transcriptomics

## Abstract

The brains of teleost fish show extensive adult neurogenesis and neuronal regeneration. The patterns of gene regulation during fish brain aging are unknown. The short-lived teleost fish *Nothobranchius furzeri* shows markers of brain aging including reduced learning performances, gliosis, and reduced adult neurogenesis. We used RNA-seq to quantify genome-wide transcript regulation and sampled five different time points to characterize whole-genome transcript regulation during brain aging of *N. furzeri*. Comparison with human datasets revealed conserved up-regulation of ribosome, lysosome, and complement activation and conserved down-regulation of synapse, mitochondrion, proteasome, and spliceosome. Down-regulated genes differ in their temporal profiles: neurogenesis and extracellular matrix genes showed rapid decay, synaptic and axonal genes a progressive decay. A substantial proportion of differentially expressed genes (∼40%) showed inversion of their temporal profiles in the last time point: spliceosome and proteasome showed initial down-regulation and stress-response genes initial up-regulation. Extensive regulation was detected for chromatin remodelers of the DNMT and CBX families as well as members of the polycomb complex and was mirrored by an up-regulation of the H3K27me3 epigenetic mark. Network analysis showed extensive coregulation of cell cycle/DNA synthesis genes with the uncharacterized zinc-finger protein *ZNF367* as central hub. *In situ* hybridization showed that *ZNF367* is expressed in neuronal stem cell niches of both embryonic zebrafish and adult *N. furzeri*. Other genes down-regulated with age, not previously associated with adult neurogenesis and with similar patterns of expression are *AGR2, DNMT3A, KRCP, MEX3A, SCML4*, and *CBX1*. CBX7, on the other hand, was up-regulated with age.

## Introduction

Several studies analyzed genome-wide transcript regulation during aging of the primate and rodent brain (Lee *et al*., 2000; Loerch *et al*., 2008; Somel *et al*., 2010) including analysis of human samples across the entire life stages (Colantuoni *et al*., 2011). Some conserved features linked to brain aging are as follows: (i) down-regulation of genes coding for synaptic and axonal proteins (Loerch *et al*., 2008), (ii) increased activity of the polycomb repressive complex (Horvath *et al*., 2012), and (iii) reduced adult neurogenesis (Kempermann, 2011). In primates, where analyses along the entire postnatal life are available, gene regulation observed during brain aging is either the continuation, or the inversion, of a process that started during development. (Somel *et al*., 2010; Colantuoni *et al*., 2011).

The brain of teleost fish, as opposed to mammals, shows widespread adult neurogenesis and remarkable regenerative properties (Kempermann, 2011). The process of brain aging in teleost has received very little attention so far (Terzibasi Tozzini *et al*., 2012; Edelmann *et al*., 2013). It is therefore of interest to investigate whether the patterns of gene regulation identified in mammals are observed also in teleosts.

The short-lived fish *Nothobranchius furzeri* is a novel model organism for aging research. *N. furzeri* inhabits ephemeral habitats that last on average 75 days (Terzibasi Tozzini *et al*., 2013), and their captive lifespan is the shortest ever recorded for a vertebrate (Genade *et al*., 2005). As an adaptation to this environment, *N. furzeri* shows explosive growth (Blazek *et al*., 2013) and rapid expression of aging phenotypes at the behavioral, histological, and molecular levels (Valenzano *et al*., 2006a; Di Cicco *et al*., 2011; Hartmann *et al*., 2011) including age-dependent gliosis and rapid decay of adult neurogenesis (Terzibasi Tozzini *et al*., 2012). The *N. furzeri* transcriptome is available, and an initial study of RNA-seq, based on pool sequencing and two age steps, has confirmed a prominent decay of mitotic activity during brain aging (Petzold *et al*., 2013). A striking characteristic of *N. furzeri* is the existence of large differences in longevity across strains (Terzibasi *et al*., 2008) that has a polygenic origin (Kirschner *et al*., 2012). Here, we used the strain MZM-04/10 that has a median lifespan of ∼ 30 weeks and was characterized in previous studies (Terzibasi *et al*., 2008; Terzibasi Tozzini *et al*., 2012).

The aim of this study was to perform a systematic analysis of genome-wide transcript regulation during brain aging, using biological replicates and multiple time points. In particular, we concentrated our analysis on the following aspects: (i) characterization of the temporal profiles of expression, (ii) comparison with human data, (iii) characterization of networks of coregulated genes, and iv) localization of cells expressing differentially expressed genes in the brain of both adult *N. furzeri* and zebrafish embryos.

## Results

### Identification of differentially expressed genes and global analysis of gene expression

To describe age-dependent transcriptome regulation, we used RNA-seq (Illumina) and a reference *N. furzeri* transcriptome containing 19 812 annotated transcript contigs. The data cover male animals, 5 age-groups (5, 12, 20, 27, and 39 weeks), and five biological replicates for each age. These ages correspond to sexual maturity, young adult, adult (as defined by a decrease in growth rate), median lifespan, and old (∼30% survivorship) (Figs S1, S2). Differentially expressed genes (DEGs) were defined by the intersection of three independent statistical tests without cutoff for effect size (see Supplementary Material and Methods) in at least one of all pairwise comparisons between the age-groups, and 4104 DEGs were detected (Table S1). The highest number of DEGs between adjacent time points was detected in the comparison between 5 and 12 weeks and the minimum between 20 and 27 weeks (Fig. S3).

To validate the findings obtained by RNA-seq, we performed qPCR on 20 DEGs (Table S2). The fold-changes measured using the two techniques are highly correlated (Pearson's *R*^2^ = 0.70; Fig. S4).

**Figure 1:**
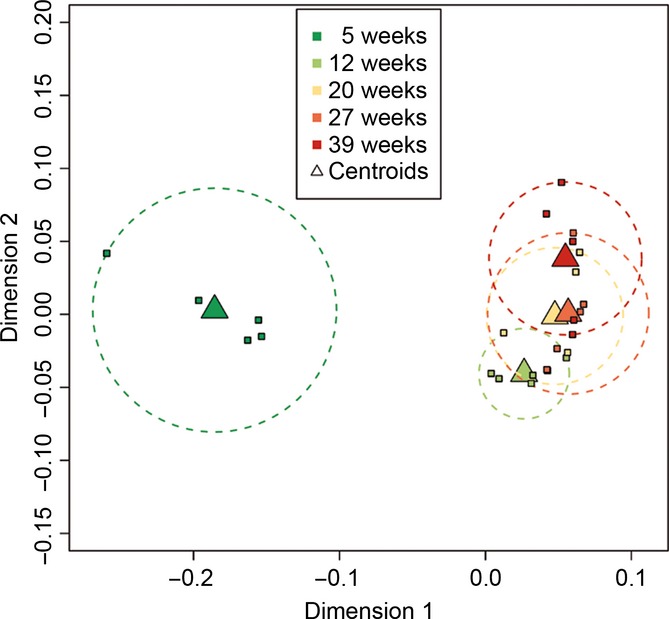
Multidimensional scaling. Age of samples is color-coded. Individual samples are represented by small squares, the centroids by triangles, and the confidence interval by dotted circles.

To visualize the impact of age on global transcript regulation, we applied multidimensional scaling (MDS) (Fig.1). Using this approach, the 5-week-, 12-week-, and 39-week-old samples can be separated in nonoverlapping regions in the MDS plot. However, the largest separation is clearly between the 5-week-old samples and the other samples in dimension 1 (see also Fig. S5), while in dimension 2, there is a progression from the 12-week-old samples to 20- and 27-week-old to 39-week-old samples.

### Clustering temporal profiles of gene expression

**Figure 2:**
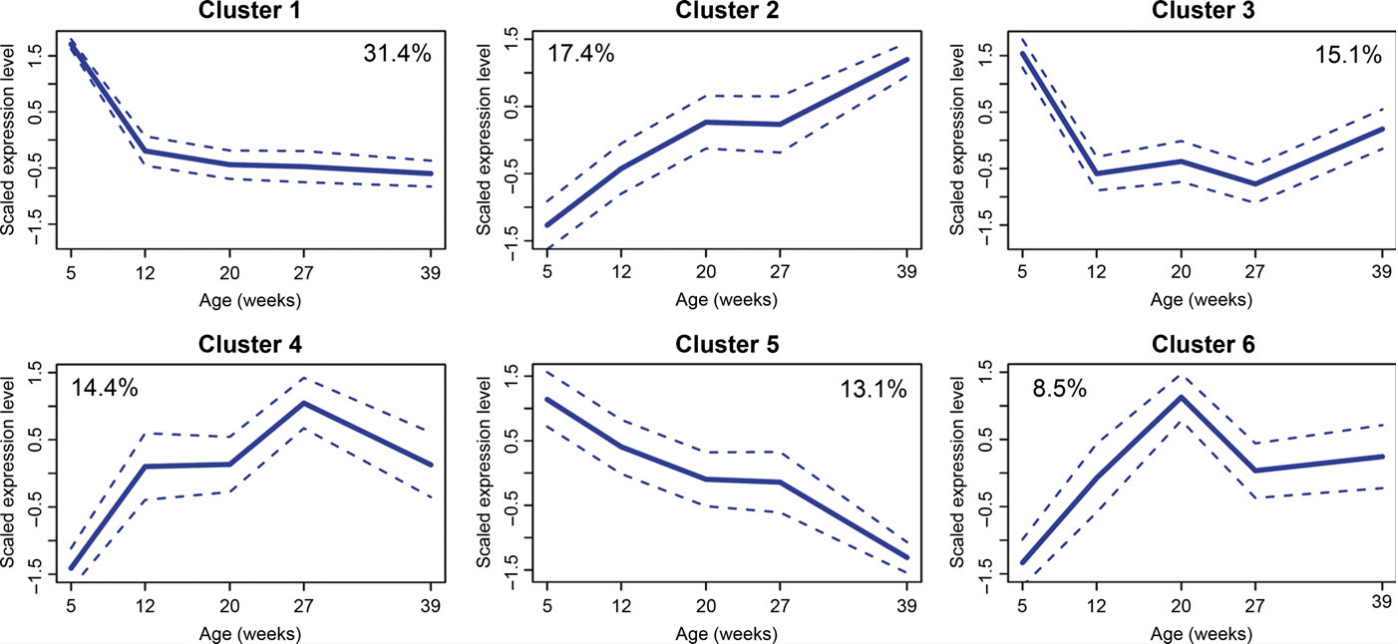
Fuzzy c-means clustering of 4014 differentially expressed genes (DEGs) according to temporal profiles. Each individual DEG profile was centered to mean and scaled to variance. The solid line represents the mean value of the cluster and the dashed lines 95% confidence intervals. For each cluster, the percentage of DEGs assigned to it is reported. Clusters are ordered according total number of members.

To group DEGs with similar temporal profiles, fuzzy c-means (FCM) clustering was used. The number of optimal clusters was estimated by the vote of different cluster validity indices, which capture different aspects of a clustering structure (Guthke *et al*., 2005). The temporal transcript profiles were grouped into 6 clusters ordered by the number of DEGs included (Fig.2 and Table S3). Three clusters exhibit a monotonic behavior: Cluster 2 shows a gradual increase, while clusters 1 and 5 show rapid and gradual decreases, respectively. The remaining clusters include more complex temporal profiles: Cluster 3 shows a U shape with a minimum at 27 weeks; clusters 4 and 6 include a peak at 27 and 20 weeks, respectively. In summary, 60.9% of DEGs were assigned to clusters with monotonous age regulation, and 39.1% were assigned to clusters with inversion in the initial direction of age regulation. 59.6% of the DEGs showed an initial decrease and 40.4% an initial increase in their expression.

As an independent assessment for inversions in the temporal profile of gene expression, we identified genes showing U- or bell-shaped trajectories by quadratic fitting (*P* < 0.05) of gene expression levels as function of age. The distributions of individual estimated peaks were plotted separately for genes showing U shape (*n* = 887) or bell shape (*n* = 2504). In both cases, the large majority of the peaks were in the 20-to 30-week range with median around 25 weeks (Fig. S6).

### Gene enrichment analysis

**Figure 3:**
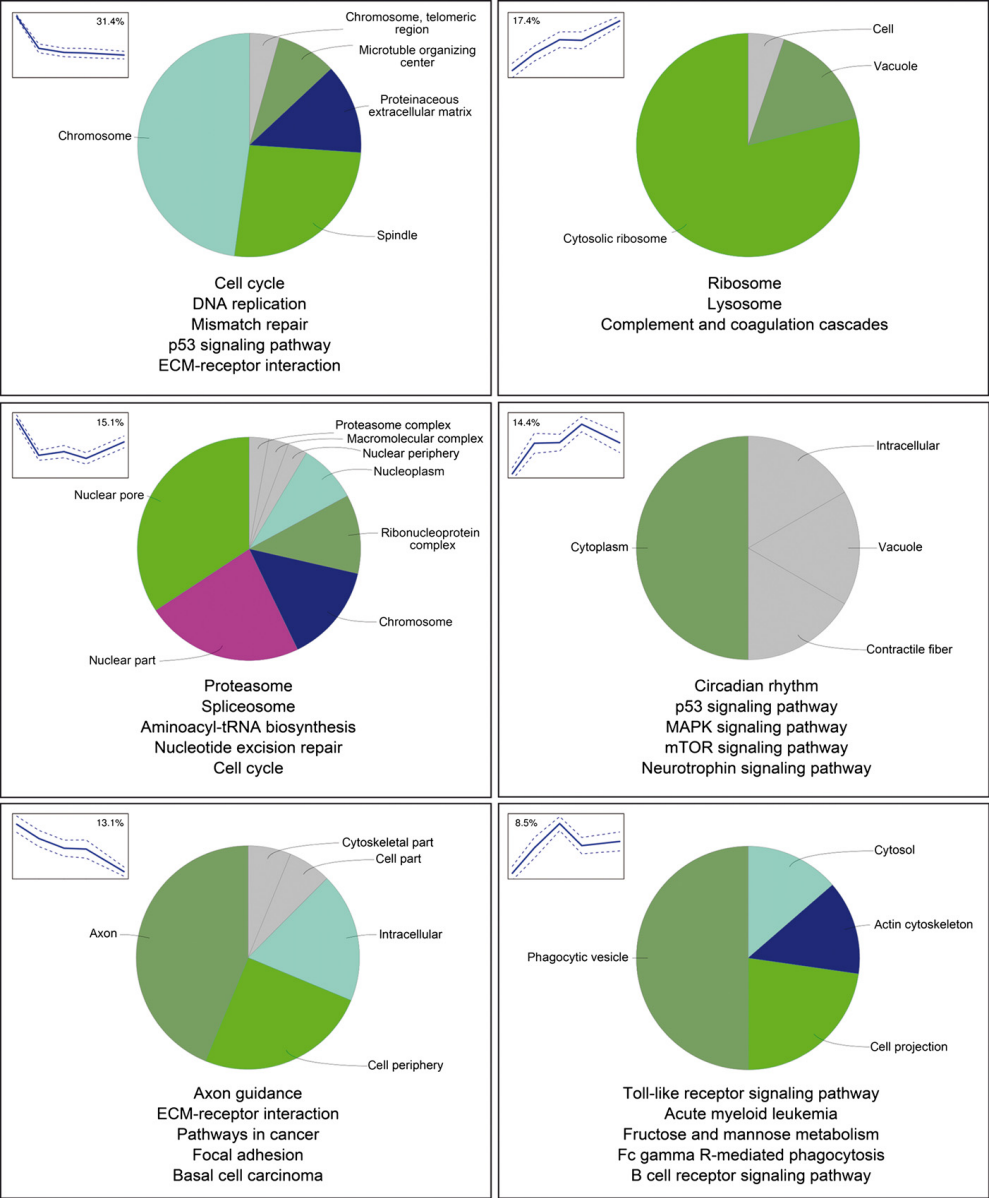
Gene enrichment analysis of the individual fuzzy c-means (FCM) clusters. The pie charts correspond to clustering of enriched cellular component terms as generated by ClueGO. The dimension of the wedges is proportional to the number of clustered terms, and the term with the highest number of differentially expressed genes was used for annotation. Gray wedges indicate individual terms that could not be clustered. The text below the pie charts reports the five most enriched KEGG pathways for each of the FCM clusters.

**Figure 4:**
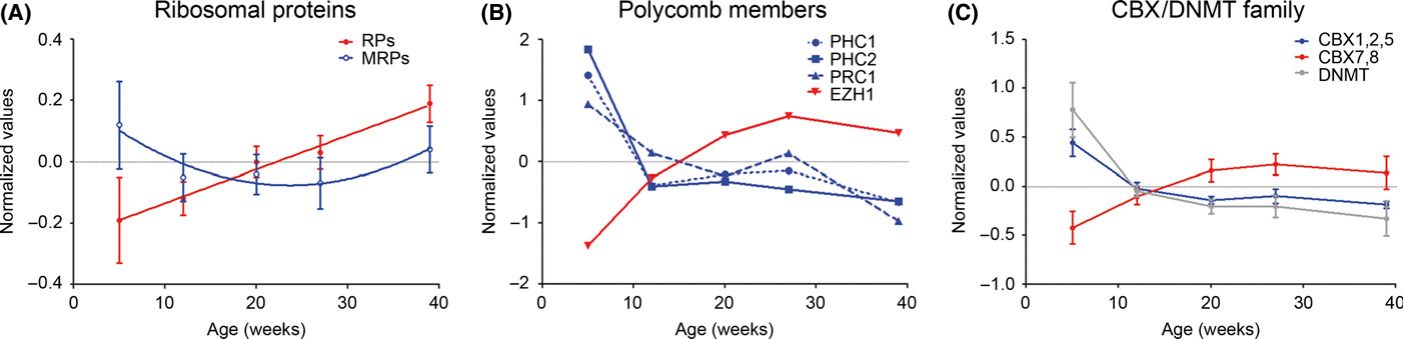
Age-dependent transcript levels of selected differentially expressed genes. Expression values at each age were centered and scaled to the mean. (A) The average of all 87 ribosomal genes (RP) and mitochondrial ribosomal genes (MRP) are shown. Error bars represent standard deviations. (B) Members of the polycomb complexes 1 and 2. (C) Averaged expression of all *DNMT* genes and members of the *CBX* genes family. CBX members in cluster 1 are shown in blue, and CBX members in clusters 2 and 6 are shown in red.

In order to assess whether different temporal profiles correspond to different cellular components or biological processes, we determined for the *N. furzeri* genes their human orthologs and performed gene enrichment analysis based on annotations of the human genes corresponding to each of the temporal clusters. In a first step, we analyzed KEGG pathways, and we report for each cluster up to the top five annotations (Fig.3) a complete list with percentage of up- and down-regulated genes (Table S4) and a mapping of fold-changes (Appendix S1). To reduce the redundancy of the large numbers of enriched gene ontology (GO) terms (Table1), we used ClueGO to cluster terms that are sharing significant number of genes and report here the results for cellular components only (Fig.3). Cluster 1 (rapid decay) was enriched for genes coding for proteins that are in nuclear components (chromosome, spindle, and telomeres) as well as extracellular matrix and microtubule-organizing center. Consistently, the most enriched KEGG pathways were ‘cell cycle’ and ‘ECM–receptor interaction’. It is of note that 10 genes of the Notch pathway are assigned to cluster 1 (Table S5). Cluster 2 (linear increase) was enriched for genes coding for proteins that are assigned to ‘cytosolic ribosome’ and ‘vacuole’. KEGG analysis showed an extensive enrichment of genes belonging to the pathways ‘ribosome’, ‘lysosome’, and ‘complement and coagulation cascades’. Averaged expression of all genes encoding cytosolic ribosomal proteins (RPs) increased linearly as function of age with 47% increase in expression between 5 and 39 weeks, while mitochondrial ribosomal proteins showed U-shaped profile (Fig.4A). The term ‘GO: 0006413 translational initiation’ is enriched as well (4.4-fold enrichment, Fisher's exact test, *P* = 0.0023). Cluster 3 (U shaped, minimum at 27 weeks) was enriched for genes coding for nuclear proteins (nuclear pore, chromosome, nucleoplasm, nucleus periphery) as well as ribonucleoprotein and proteasome complexes. KEGG analysis showed highly significant enrichment of ‘proteasome’ and ‘spliceosome’. Cluster 4 (bell shaped, peak at 27 weeks) was enriched for genes coding for proteins in cytoplasm as well as contractile fiber and vacuole. KEGG analysis showed highly significant enrichment of genes belonging to the ‘circadian rhythm’ and ‘MAPK-pathways’ and in addition ‘P53-’, ‘mTOR-’, and ‘neurotrophin-signaling-pathway’. Notably, key negative regulators of the cell cycle such as the cyclin-dependent kinase inhibitor-1 (*CDKN1A, P21*), members of growth arrest and DNA damage gene family (*GADD45*), and the TP53 target inhibitory cyclin G1 (*CCNG1*) (Okamoto & Beach, 1994), are assigned to cluster 4. Cluster 5 (linear decrease) showed significant enrichment in proteins present in neuronal processes: Interestingly, the most significantly enriched GO term for cellular components is synapse (GO:0045202, 23 genes, 3.2-fold enrichment, Fisher's exact test, *P* = 10^−6^), and further categories are cell periphery, intracellular, and cytoskeletal part. Top enriched KEGG pathways are ‘axon guidance’ and ‘ECM–receptor interaction’ (Fig.3). It is of note that three key genes of the sonic hedgehog (SHH) signaling pathway, a pathway known to be essential for adult neurogenesis (Favaro *et al*., 2009), are assigned to this cluster (Table S5). Cluster 6 (bell shaped, peak at 20 weeks) was enriched for genes belonging to KEGG pathways related to innate immunity.

**Table 1:** GO categories common between *Nothobranchius furzeri* and human brain aging

Category	Human	Intersection	*N. furzeri*	Top categories	FDR
Overrepresented terms among up-regulated genes					
GO: biological process	194	56 (40%)	142	Translational elongation	1.6e-41
				Protein targeting to ER	1.4e-37
				Nonsense-mediated decay	1.5e-35
GO: cellular compartment	72	20 (77%)	26	Ribosome	1.4e-41
				Lysosome	6.3e-6
GO: molecular function	22	4 (80%)	5	Structural constituent of ribosome	1.7e-25
KEGG pathway	2	1 (50%)	2	Ribosome	1.4e-38
Overrepresented terms among down-regulated genes					
GO: biological process	349	116 (32%)	364	Cell cycle	2.6e-59
				DNA damage	1.7e-22
				Cellular response to stress	5.8e-15
				Protein folding	2.9e-06
				RNA processing	6.2e-06
GO: cellular compartment	201	59 (41%)	143	Nucleus	3.9e-39
				Organelle	6.8e-39
				Microtubule cytoskeleton	3e-16
				Mitochondrion	1.1e-10
GO: molecular function	99	27 (33%)	82	ATPase activity	4.6e-7
				RNA binding	4.9e-05
				Threonine-type peptidase activity	0.00016
				Unfolded protein binding	0.00044
KEGG pathways	25	5 (25%)	20	Proteasome	2.3e-5
				RNA transport	0.00017
				Spliceosome	0.00019
				Nucleotide excision repair	0.00038
				Protein processing in endoplasmic reticulum	0.0006

The numbers reported refer to categories significant with hypergeometric test and FDR < 0.05. Human and *N. furzeri* figures refer to the total number of categories in each gene set, respectively. Percentage in intersection column refers to percentage of *N. furzeri* categories. The top categories are defined as nonoverlapping categories ranked on FDR-corrected *P*-value for the *N. furzeri* dataset whose values are reported in the last column.

### Chromatin-remodeling genes

Age-dependent epigenetic remodeling is prominent in the human brain (Horvath *et al*., 2012; Numata *et al*., 2012; Watson *et al*., 2012), and chromosome is a predominant GO term in cluster 1 (Fig.3). We examined the chromatin-remodeling DEGs and found that several members of the DNA methyl-transferase gene family were down-regulated and belong to cluster 1 (*DNMT1/3B/3AA/3AB,* Fig.4C). Particularly evident was the regulation of members of the polycomb repressive complex 1: protein regulator of cytokinesis (*PRC1*), polyhomeotic-like 1 and 3 (*PHC1* and *PHC 2*; cluster 1), and enhancer of zeste homolog 1 (*EZH1*; cluster 4), that is part of polycomb complex 2 (Fig.4B). Similarly, members of the chromobox homolog (*CBX*) gene family showed opposite trajectories of expression (Fig.4C) with *CBX1A/B*,*CBX2,* and *CBX5* in cluster 1 and *CBX7A/B* and *CBX8A/B* in clusters 2 and 6.

### Comparison with other datasets

We compared the list of DEGs obtained in *N. furzeri* with a public dataset of 269 samples from human prefrontal cortex from fetal to old age (Colantuoni *et al*., 2011). DEGs from *N. furzeri* are regulated during human ontogeny, and FCM clustering separates them into three groups: (i) rapid decrease during fetal life, (ii) peak during childhood followed by a decrease, and (iii) increase from fetal life to childhood (Fig. S7). To specifically compare aging-related DEGs from human and *N. furzeri*, we used a dataset of 13 young (age 25–40) and 15 old subjects (age 70–95) (Loerch *et al*., 2008) and performed gene enrichment analysis on human and *N. furzeri* DEGs separately. For both down- and up-regulated GO terms, we detected an intersection that included between 25% and 80% of the *N. furzeri* terms and that was particularly extensive for up-regulated DEGs (Table1). For a broad-level comparison, we used a meta-analysis of human brain, kidney, and muscle microarray data (Zahn *et al*., 2006) and a meta-analysis of different tissues from human, primate, and rodent tissues (de Magalhaes *et al*., 2009). Four up-regulated terms (‘cytosolic ribosome’, ‘lysosome’, ‘negative regulation of apoptosis’, and ‘complement activation’) were shared between *N. furzeri,* and at least one of these analysis and two down-regulated terms (‘mitochondrion’ and ‘collagen’) were shared between de Magalhaes *et al*. (2009) and *N. furzeri* (Table2). In addition, 16% of the up-regulated genes and 21% of the down-regulated genes identified by de Magalhaes *et al*. (2009) were differentially expressed with concordant direction in *N. furzeri* (Table S9 and Fig. S12).

**Table 2:** Comparison of the meta-analysis of de Magalhaes *et al*. (2009) and Zahn *et al*. (2006) with genes differentially expressed in *Nothobranchius furzeri* brain

Meta-analysis by de Magalhaes *et al*. (2009)	Meta-analysis by Zahn *et al*. (2006)	This study
Overrepresented terms among down-regulated genes		
Mitochondrion	ND	GO:0005739 mitochondrion, *P* = 2.5 × 10^−5^
Oxidative phosphorylation	Oxidative phosphorylation	ND
Hydroxylisine, hydroxylation, collagen	ND	GO:0005581 collagen, *P* = 1.5 × 10^−5^
Overrepresented terms among up-regulated genes		
Immune response, complement activation	Complement activation	KEGG hsa04610 complement and coagulation cascades, *P* = 0.035
ND	Cytosolic ribosome	KEGG hsa03010 ribosome, *P* = 8.4 × 10^−49^
Lysosome	ND	KEGG hsa04142 lysosome, *P* = 0.0028
Negative regulation of apoptosis	ND	GO:0043066 negative regulation of apoptosis, *P* = 0.0053

The terms reported for meta-analysis are obtained from Table I de Magalhaes *et al*. (2009) and represent the broadest of the top annotation of functional clusters obtained by DAVID. The terms reported for Zahn *et al*. (2006) were detected by GSEA using correlation of gene expression with age. *N. furzeri DEGs* were analyzed by DAVID using standard setting and the ‘FAT’ level for GO terms. Significance values refer to Fisher's exact test.

### Network analysis

**Figure 5:**
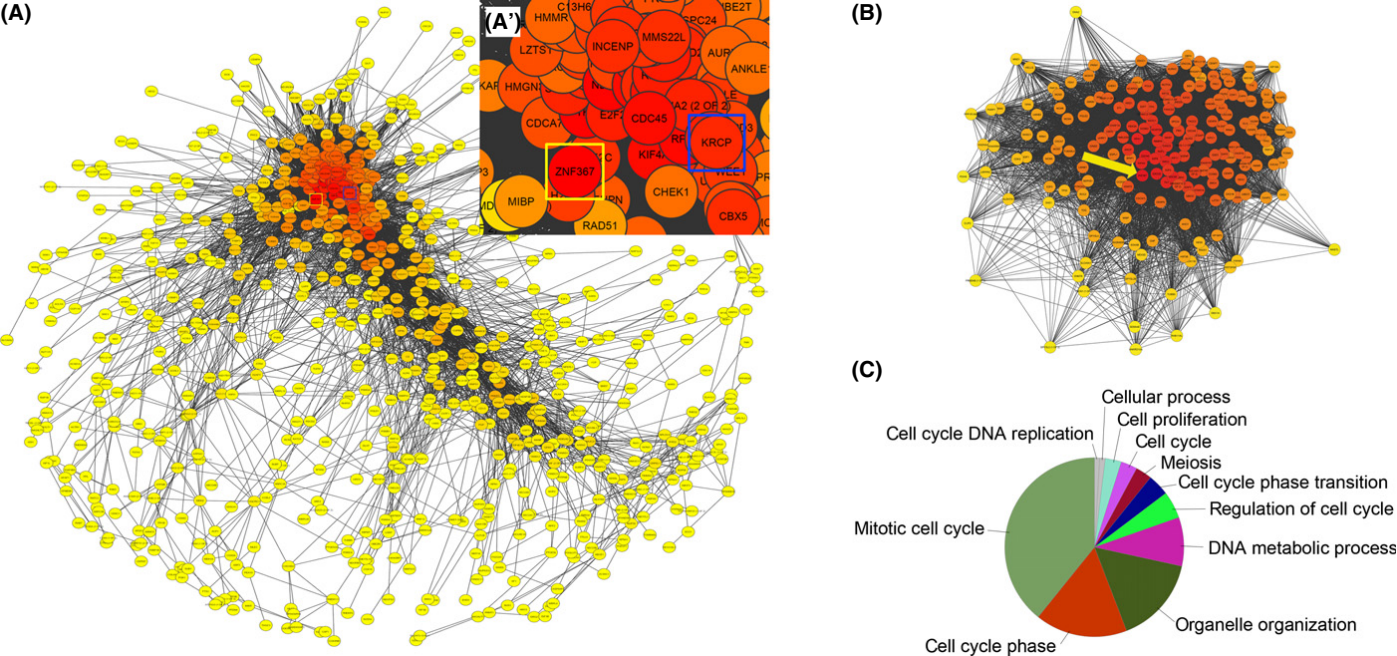
Network analysis. (A) Network of RKPM values of all expressed genes based on Pearson's correlation r ≥ 0.95, spring-embedded layout weighted on correlation. Color of nodes indicates the edge degree value with red assigned to nodes of highest values. Only the cluster with largest number of nodes is shown. (A′) Enlargement of (A): *ZNF367*, the node of highest edge, is indicated by yellow square, the position of *KRCP*, a gene of high edge degree value that was further studied by *in situ* hybridization, is indicated by a blue square. (B) Network of the first neighbors of *ZNF367*, 192 nodes, 1030 edges. The position of *ZNF367* is indicated by a yellow arrow. (C) Gene enrichment analysis of *ZNF367* first neighbors.

We constructed a correlation-based network of the transcript levels of all expressed genes in the 25 samples. Using Pearson′s r > 0.95 as selection criterion for edges, the resulting network contains 1707 nodes and 18 008 edges (Fig.5A). The node of highest edge value is the putative transcription factor zinc-finger protein 367 gene (*ZNF367*) in cluster 1 (Fig.5A'). The subnetwork of *ZNF367* first neighbors (Fig.5B) contains 192 nodes and 1030 edges and is clearly enriched for genes that regulate the cell cycle (Fig.5C).

We further analyzed whether correlation in expression changes as function of age and computed correlation-based networks of the DEGs only across samples of the same age for the five time points separately (Fig. S8). In the 5-week age-group, one single network emerges and contains genes belonging to all above-described FCM clusters. In the last time point, 39 weeks, two distinct clusters emerge that are not connected by edges: one contains mainly genes of FCM clusters one and five (continuous down-regulation) and the second mainly genes of FCM clusters two and three that increase in expression between 27 and 39 weeks.

### Expression in the brain and identification of genes related to adult neurogenesis

**Figure 6:**
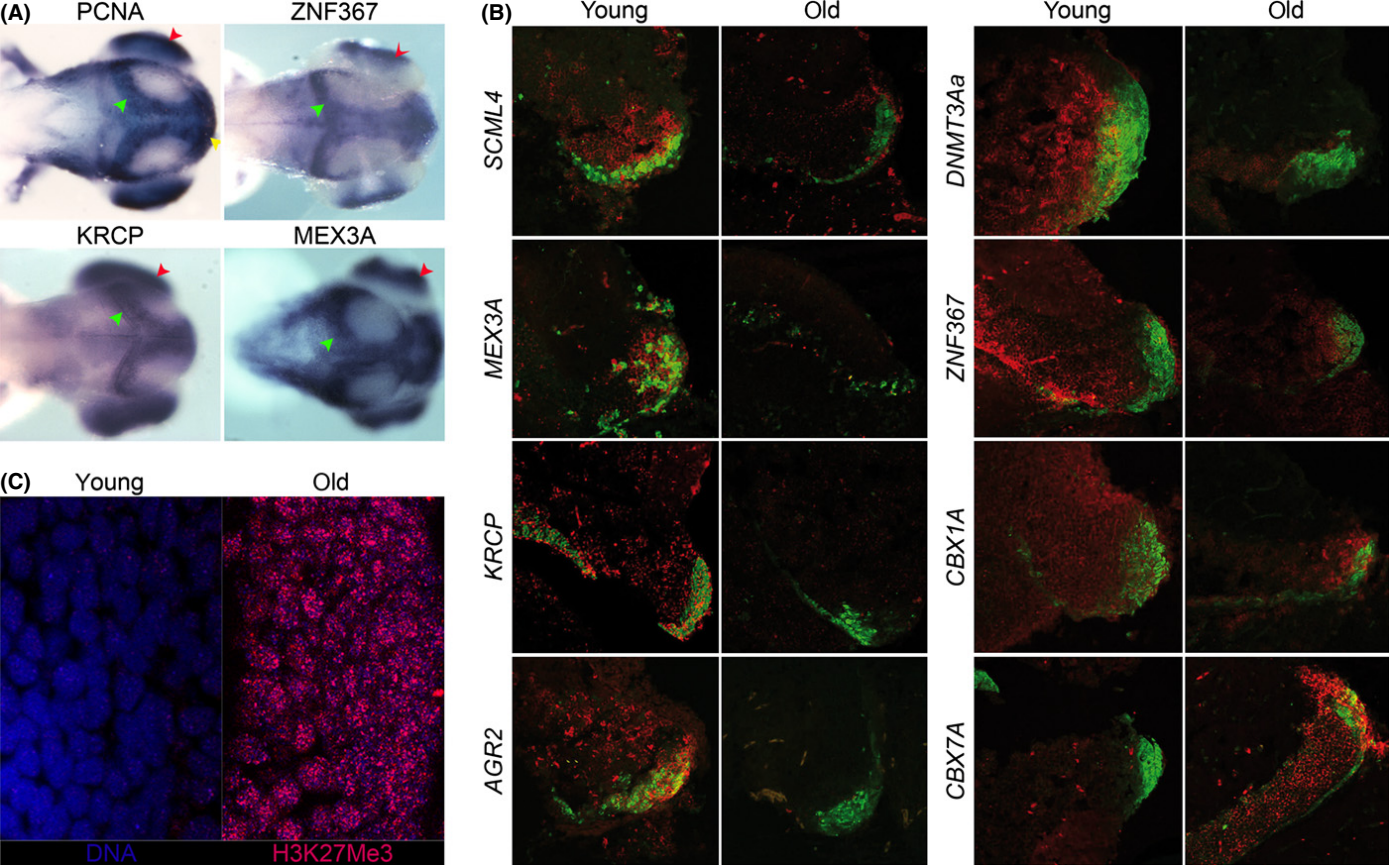
*In situ* hybridization and immunohistochemistry. (A) Dorsal view of 72 hpf zebrafish embryos processed by *in situ* hybridization for *ZNF367*,*KRCP,* and *MEX3A*. Proliferating cell nuclear antigen (*PCNA*) riboprobe has been used to visualize proliferative region of the zebrafish larval brain in the optic tectum (green arrowhead) and retina (red arrow head). (B) Double-labeling *in situ* hybridization (ISH) and immunohistochemistry (IHC) on the posterior margin of the optic tectum (OTp) in *Nothobranchius furzeri* for the following genes: *SCML4*,*MEX3A*,*KRCP*,*AGR2*,*DNMT3AA*,*ZNF367*,*CBX1A*, and *CBX7A* – ISH signal was revealed using fluorescent Fast Red and is visualized in red, PCNA IHC is visualized in green to localize the proliferative niche of the OTp. Only merged signals are shown. Young animals were 6 weeks old, and old animals were 25 weeks old. Separated red and green channels are reported in Fig. S10. The images are representative of three replicates. (C) Changes of PRC2 markers with age. Immunohistochemical detection of H3K27me3 in the posterior optic tectum of young (5 weeks) and old (35 weeks) individuals. The images are representative of three replicates. Immunohistochemical signal is shown in red and DNA staining (TOPRO3) in blue. Overlap of signals gives rise to a purple hue.

Cluster 1 is highly enriched for genes related to cell cycle and DNA replication and shows a rapid decay. Adult neurogenesis is known to decrease exponentially with age (Pekcec *et al*., 2008; Ben Abdallah *et al*., 2010), and we reasoned that this cluster may contain genes specifically enriched in the niches of neuronal stem cells (NSCs) and not previously characterized in this context. As a first step, we interrogated the ZFIN database and identified 96 genes of cluster 1 for which high-quality *in situ* hybridization pictures in zebrafish embryos were available. Of these, 86% (83/96) are expressed in the nervous system and 50% (48/96) are expressed specifically in the NSC niches of the zebrafish embryo (Table S6). We selected a short list of genes of putative regulatory function to visualize their expression by *in situ* hybridization in the central nervous system of zebrafish embryos at age 72 h when niches of NSCs are well delimited (Figs6A and S9). These genes were anterior gradient related 2 (*AGR2*), previously implicated in regeneration of amphibian limbs (Kumar *et al*., 2007), the chromatin remodelers *CBX1A*,*CBX7A*,*DNMT3AA,* and sex comb midleg like 4 (*Drosophila*) (*SCML4*), and the RNA-binding protein mex3 homolog A (*C. elegans*) (*MEX3A*) (Buchet-Poyau *et al*., 2007). In addition, we analyzed the genes for two putative transcription factors: *ZNF367* and the teleost-specific kelch repeat containing protein (*KRCP*) that both occupy very central positions in the correlation-based network (Fig.5A, A′). The expression of *ZNF367, KRCP,* and *MEX3A* was very similar to the expression of the mitotic marker proliferating cell nuclear antigen (*PCNA,* Fig.6A). Neuronal expression of *AGR2* was detected exclusively in the mitotically active olfactory epithelium (Fig. S9). All other genes (Fig. S9) were detected in the ciliary marginal zone that represents the NSC niche of the retina and showed different expression patterns in the other NSC niches. Expression of *SCML4* was widespread caudally, but was restricted rostrally to NSC niches in the optic tectum, retina, and olfactory epithelium. *CBX1A* and *DNMT3AA* showed more widespread expression in the developing brain. *CBX7A* was not expressed at detectable levels in zebrafish embryos.

We then performed double-labeling *in situ* hybridization with PCNA in the *N. furzeri* brain at 5 and 25 weeks of age (Figs6B and S10) focusing attention on the posterior margin of the optic tectum that represents one of the most active NSC niches in *N. furzeri* brain (Terzibasi Tozzini *et al*., 2012). Mitotically active cells were identified by double labeling with antibodies against PCNA. All these genes were expressed in the NSCs in an age-dependent manner (Fig.6B): *CBX7A* was not detected in young animals and showed a widespread expression in old animals including both the postmitotic neurons and the stem cell niche. This was opposite to the expression of *CBX1A* that was widespread in young animals and down-regulated in old animals with expression concentrated in the NSC niche. *DNMT3AA*,*KRCP*,*MEX3A*,*SCML4,* and *ZNF367* all were associated with the NSC niche in young animals, and their expression was down-regulated as a function of age. To obtain an independent confirmation for differential activity of polycomb members, we performed immunohistochemistry for tri-methylation of lysine 27 of histone H3 (H3K27me3) that is a known repressive modification linked to PRC2 activity (Simon & Kingston, 2013). Analysis of optic tectum revealed a clear up-regulation of H3K27me3 signal in old fish (Fig.6C).

To investigate the regulation of these genes in humans, we interrogated the ‘Braincloud’ database (Colantuoni *et al*., 2011) that reports expression in the prefrontal cortex in 269 human subjects from fetal stage to 80 years. Five of these eight genes show a pattern of age-dependent regulation concordant to *N. furzeri*:*CBX7* is strongly up-regulated starting after birth, *CBX1* and *ZNF367* are strongly down-regulated between fetal ages and newborn, *MEX3A* and *DNMT3A* show more progressive down-regulation, and *SCML4* is up-regulated during fetal life and down-regulated during postnatal life (Fig. S11).

## Discussion

In the present paper, we analyzed age-dependent genome-wide transcript regulation in the short-lived fish *N. furzeri* by means of RNA-seq. Analysis of aging-dependent gene regulation requires the use of multiple time points, as different genes may follow different trajectories and reach their peaks or plateaus at different times. In this study, we sampled five time points during adult life starting from sexual maturity and including individuals in the ‘old’ class (around 30% survivorship) to resolve different age-dependent expression profiles. In addition, we compared our results with public data on gene regulation in the aging human cortex and detected conservation in the regulated pathways and GO groups.

### Temporal inversions

We observed that almost 40% of the genes showed U or bell shape mostly with inversion at 27 weeks and a change of gene coregulatory networks in the oldest group. This could be the result of heterogeneity in the rate of aging and selection for individuals with retarded aging at the oldest group. This cluster of bell-shaped genes contains *GADD45* family members that are typically activated by a variety of stressors and induce apoptosis or senescence *via* the MAPK and TP53 pathways (Liebermann *et al*., 2011) and the cell cycle inhibitors CDKN1A and CCNG1. The U-shaped cluster is enriched for cell cycle and DNA replication genes (Table S4), suggesting that the oldest group on average shows lower levels of cellular stress than the animals sampled at median lifespan, possibly because they delayed or escaped age-dependent dysfunctions, as it is the case for human centenarians (Evert *et al*., 2003). Inversions in the direction of age-dependent gene expression are prominent in the postnatal primate cortex (Somel *et al*., 2010; Colantuoni *et al*., 2011) and also observed in the rat brain (Wood *et al*., 2013). One of these turning points is observed around the age of 60 years in humans (Colantuoni *et al*., 2011). Our observation that in *N. furzeri,* the frequency of turning points peaks shortly before median lifespan suggests that this may be a general phenomenon in vertebrates.

### Up-regulation of ribosomal genes and protein homeostasis

A surprising observation of the present study is a concerted and progressive up-regulation of genes coding for ribosomal proteins accompanied by up-regulation of genes involved in initiation of translation. Up-regulation of ribosomal protein genes was detected also by a meta-analysis of human brain, kidney, and muscle datasets (Zahn *et al*., 2006). Enrichment of lysosome-related terms, such as cathepsins, is observed in the same cluster and appears as a conserved signature of aging in a meta-analysis of microarray experiments (de Magalhaes *et al*., 2009). Aging is linked to disrupted protein homeostasis and accumulation of misfolded proteins (David *et al*., 2010) that is also a unifying mechanism of progressive neurodegenerative diseases (Ross & Poirier, 2004). In parallel, accumulation of lysosomal aggregates (lipofuscin) is a well-characterized histopathological hallmark of neuronal aging (Jonker *et al*., 2013; Terzibasi Tozzini *et al*., 2013). Finally, down-regulation of proteasome is observed both in *N. furzeri* and human (Table1), and the network of coregulated genes at the oldest age shows enrichment for ER stress response and protein folding (Fig. S8). All these data indicate that impaired protein homeostasis is a conserved hallmark of brain aging.

### Different profiles of down-regulation

Clustering down-regulated genes according to temporal profiles revealed two different kinetics of down-regulation: progressive and rapid. Both clusters are enriched for ECM terms clearly showing that brain aging in *N. furzeri* is associated with major remodeling of the extracellular matrix, as already shown for the skin by an initial study of RNA-seq based on pool sequencing (Petzold *et al*., 2013) and this is a conserved hallmark of aging across several tissues (de Magalhaes *et al*., 2009). However, several other GO terms and KEGG pathways are differentially represented in the two clusters. Of particular interest is the down-regulation of synaptic proteins that is also a feature of the aging primate brain (Loerch *et al*., 2008; Somel *et al*., 2010) and indicates a progressive deterioration of neuronal function/plasticity during aging that underlies the age-related learning impairments observed in *Nothobranchius* (Valenzano *et al*., 2006b).

Rapid decay of gene expression (i.e. large differences between 5 and 12 weeks) can be partly related to the continuation of somatic growth past sexual development that is a typical trait of teleost fish. However, a fraction of DEGs were clearly associated with age-dependent decrease in neurogenesis in agreement with previous histological and transcriptomic studies (Terzibasi Tozzini *et al*., 2012; Petzold *et al*., 2013). As the number of neuronal stem cells (radial glia) does not decrease during aging (Terzibasi Tozzini *et al*., 2012), this difference should be primarily be due to reduced activity of the stem cells that are physically preserved but become increasingly quiescent.

Genes with initial down-regulation and increase in the last point, on the other hand, were enriched for genes coding for proteins of proteasome and spliceosome, and these terms were enriched also in a dataset of human cortex aging (Loerch *et al*., 2008; Table1). It is highly interesting that RNA-seq studies have shown that both splicing (Wood *et al*., 2013; Mazin et al., 2013) and the expression of genes coding for splicing factors (Mazin *et al*., 2013) show a U-shape profile during mammalian brain ontogeny.

### Morphogen pathways and aging

Morphogens are known to regulate the state of adult NSCs, and Notch pathway is important in regulating quiescence in teleost fish (Chapouton *et al*., 2010). In mammals, Notch signaling is activated in NSCs (Lugert *et al*., 2010), and Notch activity in the NSC niche is down-regulated during aging of the mouse (Sun *et al*., 2013). In *N. furzeri* as well, Notch pathway is down-regulated with age and is enriched in cluster 1 (rapid decay).

The sonic hedgehog (*SHH*) pathway, in combination with SOX2, is also known to regulate adult NSC activity in mammals (Favaro *et al*., 2009). *SHH* pathway is down-regulated with age but with a more progressive time course as opposed to Notch. This suggests that during the course of aging, there is an imbalance between the activities of these two pathways.

### Circadian rhythm genes

Circadian rhythm genes are highly enriched in cluster 4 (peak at 27 weeks). All animals in the study were sampled at the same time and in fasted state, and it can be excluded that this difference is due to sampling artifacts. In adult zebrafish, genes of the circadian clock are highly expressed in the NSC niches (Weger *et al*., 2013), and their regulation might be a consequence of age-dependent decay of adult neurogenesis.

### Chromatin remodeling and brain aging

Aging of primate brain is associated with prominent remodeling of chromatin (Horvath *et al*., 2012). In *N. furzeri,* histone core proteins of the H2A and H3 family are rapidly down-regulated with age. Chromatin-remodeling genes are also age-regulated, in particular DNMT and polycomb genes. DNMTs decrease rapidly with age and are known to regulate neurogenesis and neuronal differentiation by promoting expression of neurogenic genes *via* antagonization of polycomb repression (Wu *et al*., 2010). In human brain and peripheral tissues, polycomb-mediated methylation increases with age (Horvath *et al*., 2012). Polycomb members are dynamically regulated in *N. furzeri*. *EZH1*, the catalytic element of polycomb repressive complex 2 (PRC2) that catalyzes the tri-methylation of K27 on H3 (H3K27me3) (Simon & Kingston, 2013), is up-regulated with age and so is H3K27me3. As for CBX family, *CBX7*, whose activity is dependent on PRC2 (Morey *et al*., 2012), is up-regulated. On the other hand, *CBX* family members that are antagonistic to CBX7 (Morey *et al*., 2012) are down-regulated and so are components of PRC1: *PRC1, PHC1, and PHC2*. These data suggest that brain aging in *N. furzeri* is associated with imbalance in the activity of PRC1 and PRC2.

### Identification of novel genes associated with neuronal stem cell niches

Adult neurogenesis is sustained by adult NSC (aNSCs). Mammalian aNSCs are restricted to defined regions, and transcriptome analysis of aNSCs in mammals requires their purification by fluorescence-assisted cell sorting (Beckervordersandforth *et al*., 2010). Teleost brains, on the other hand, contain widespread aNSCs and no astroglia. Activity of aNSCs decreases exponentially with age (Kempermann, 2011) and is also rapidly down-regulated in *N. furzeri* between 5 and 12 weeks and further decreased at 25 weeks (Terzibasi Tozzini *et al*., 2012). Due to the large number of aNSCs, decreased neurogenesis generates in *N. furzeri* a signal detectable in whole-brain transcriptome. We identified seven genes of possible regulatory function specifically associated with aNSCs in *N. furzeri* that are also expressed in at least one neurogenic region of the zebrafish embryo. Five of these genes are down-regulated during ontogeny of the human prefrontal cortex (Colantuoni *et al*., 2011). Some of the identified genes are of unknown function in the nervous system. *MEX3A* codes for an RNA-binding protein that, in intestinal cells, regulates *MSI1* (Pereira *et al*., 2013), a neurogenic gene expressed in *N. furzeri* NSCs (Terzibasi Tozzini *et al*., 2012). *ZNF367* and *KRCP* are putative transcription factors that act as central nodes in gene coregulation networks, and their function in the nervous system is unknown. *SCML4* is a putative members of the polycomb repressive complex, *DNMT* and *CBX* family members are known to regulate the self-renewal capacity of embryonic stem cells in an interplay that is dependent on activity of the polycomb repressive complex (Wu *et al*., 2010). Our data strongly suggest that regulation of chromatin-remodeling genes is associated with aging of aNSCs *in vivo* and that these transcripts are regulated during human ontogeny.

## Materials and methods

Materials and Methods are available in the Supplements.

## Funding

No funding information provided.

## Data deposition

Data of this experiment were deposited in gene expression omnibus (GEO), Accession No. GSE52462.

## Supporting Information

**Fig. S1** Growth curve of *N. furzeri* MZM-04/10. **Fig. S2** Survivorship of *N. furzeri* MZM-04/10. **Fig. S3** Number of DEGs in pairwise comparisons. **Fig. S4** Validation by qPCR. **Fig. S5** Sample clustering. **Fig. S6** Distribution of inversion points. **Fig. S7** Clustering of human orthologs of *N. furzeri* DEGs. **Fig. S8** Age-dependent networks. **Fig. S9***In situ* hybridization in zebra fish embryos. **Fig. S10***In situ* hybridization in *N. furzeri* with separated channels. **Fig. S11** Expression plots of selected genes from Braincloud. **Fig. S12** Overlap of DEGs between this study and de Magalhaes *et al*.

Table S1 RPKMs of all genes and list of differentially expressed genes.

Table S2 List of genes analyzed by qPCR.

Table S3 Cluster membership of DEGs.

Table S4 Enriched KEGG pathways.

Table S5 List of down-regulated genes related to morphogen pathways.

Table S6 Genes of cluster 1 with expression present in ZFIN.

Table S7 Primers used to generate zebra fish ISH probes.

Table S8 Primers and sequences used to generate *N. furzeri* ISH probes.

Table S9 Intersection of DEGs with de Magalhaes *et al*.

Appendix S1 Graphical representation of regulated KEGG pathways with rendering of the fold-changes.
