# Supplementary material for: RNA-seq of the aging brain in the short-lived fish N. furzeri – conserved pathways and novel genes associated with neurogenesis
Source: Aging Cell. 2014 Jul 25;13(6):965–74. doi: 10.1111/acel.12257 (PMC4326923; doi:10.1111/acel.12257)
Supplement: Supplementary file 1 — Fig. S1 Growth curve of N. furzeri MZM-04/10. Fig. S2 Survivorship of N. furzeri MZM-04/10. Fig. S3 Number of DEGs in pairwise comparisons. Fig. S4 Validation by qPCR. Fig. S5 Sample clustering. Fig. S6 Distribution of inversion points. Fig. S7 Clustering of human orthologs of N. furzeri DEGs. Fig. S8 Age-dependent networks. Fig. S9In situ hybridization in zebra fish embryos. Fig. S10In situ hybridization in N. furzeri with separated channels. Fig. S11 Expression plots of selected genes from Braincloud. Fig. S12 Overlap of DEGs between this study and de Magalhaes et al. [file acel0013-0965-sd1.pdf]

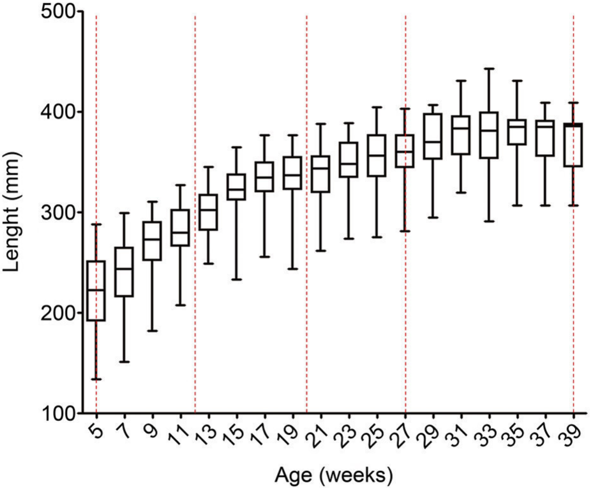

Figure S1 Growth curve of the animals in the present study as box plots. The bars represent the two extreme values, the box the 25%-75% percentile and the line the median. The red dotted lines represent the sampling times.

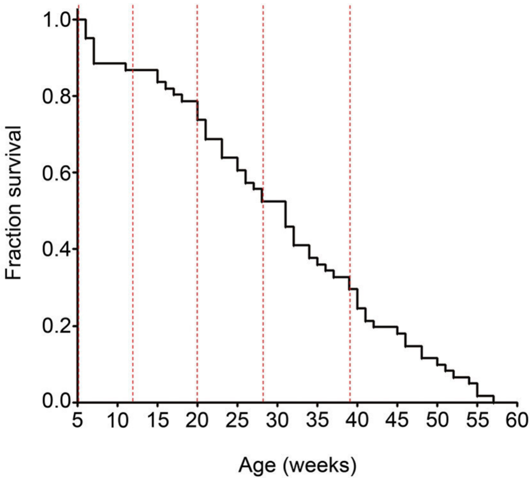

Figure S2 Representative survival curve of the strain MZM-04/10p in our facility.The red dotted lines represent the sampling times.

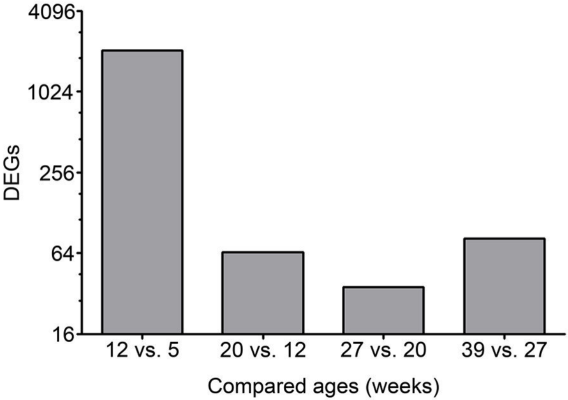

Figure S3 Number of DEGs of the four pairwise-comparisons for successive time points. Each point represents the transition from one age step to the next. Note logarithmic scale on the Y axis.

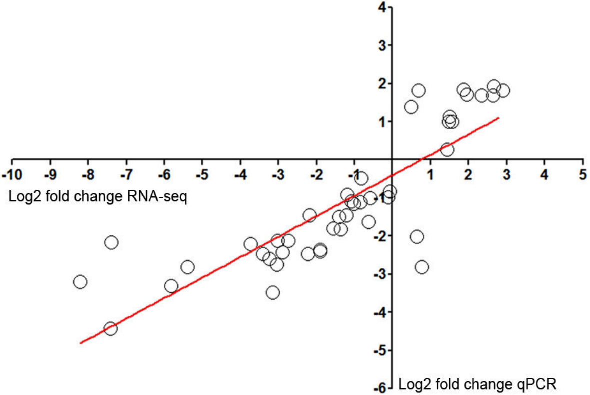

Figure S4 Correlation of qPCR and RNA-seq for 20 genes. Fold-changes were always relative to the expression value at 5 weeks. Each point represents one gene and one single comparison, so 13 gene are represented by two points 5, 12, 27 weeks analyzed) and seven by three points (also 39 weeks) (for list of genes see Table S2).

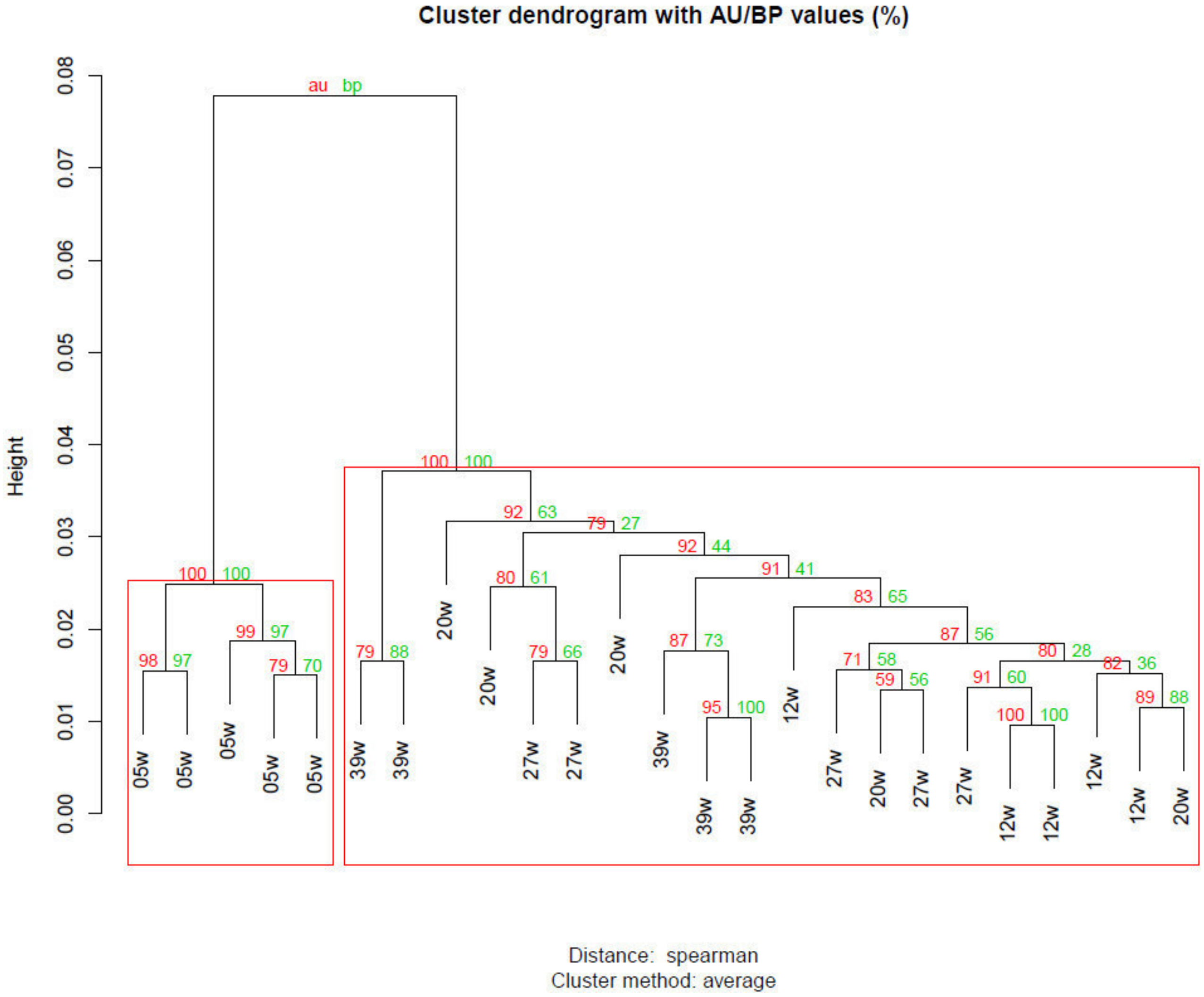

Figure S5 Hierarchical clustering

The figure shows the results from hierarchical cluster analysis using the pvclust R package (Suzuki and Shimodaira1, 2006). Pvclust provides two types of p-values: AU (Approximately Unbiased) p-value and BP (Bootstrap Probability) value.. As input data the unnormalized gene counts of all 4104 DEG were used. Spearman correlation was used as distance measure and the number of bootstrap replications was 1,000. All other parameters were set as default. Clusters with AU p-value > 0.95 were highlighted in red rectangles

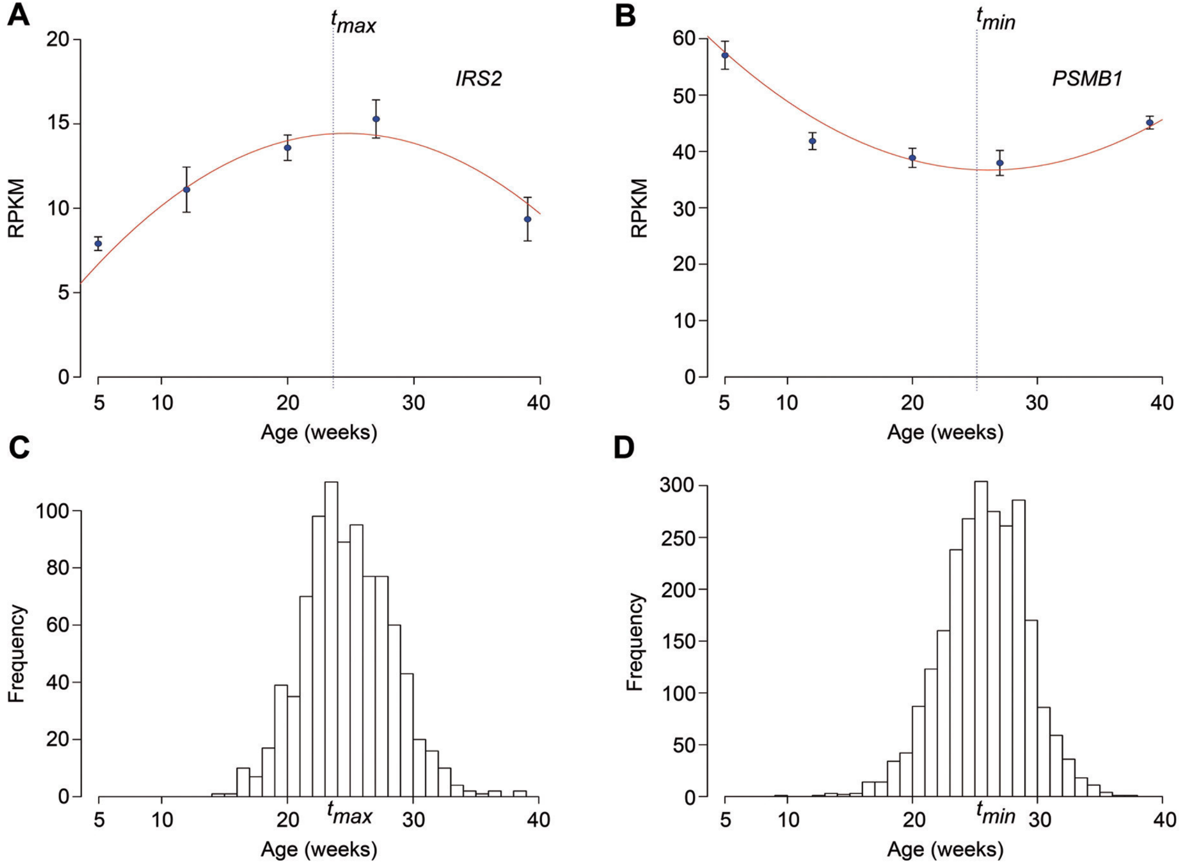

Figure S6 Assessment for inversion time points in the temporal profile of gene expression for U- or bell-shaped trajectories by quadratic fitting. (A, B) Examples of individual genes. (C, D) Distribution of the estimated time of the maximal/minimal transcript levels for the genes showing initial up-regulation (C) or down-regulation (D).

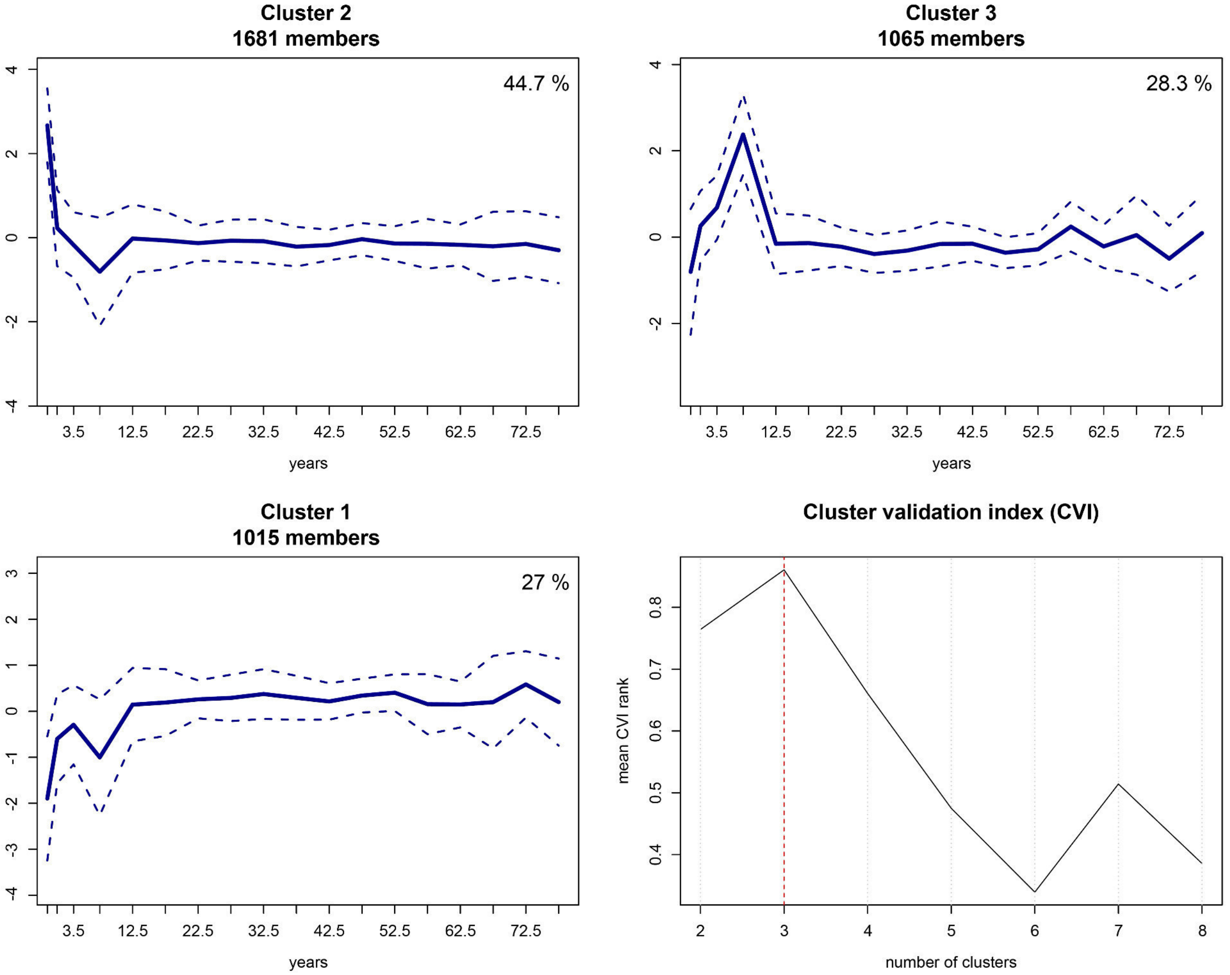

Figure S7 Clustering of human orthologs of *N. furzeri* DEGs. FCM Clustering of public expression data from braincloud (http://braincloud.jhmi.edu/). Samples were grouped in the following way: prenatal life, 1 year, 3.5 years, 7.5 years and in 5-years bins after that age. Each individual gene expression profile was centered to mean and scaled to variance. The solid line represents the mean value of the cluster and the dashed lines 95% confidence intervals. For each cluster, the percentage of genes assigned to it is reported. Clusters are ordered according total number of members. The optimal number of clusters was decided by the vote of different indices as described in Guthke et al., 2006. The cluster validation index is reported in lower right graph.

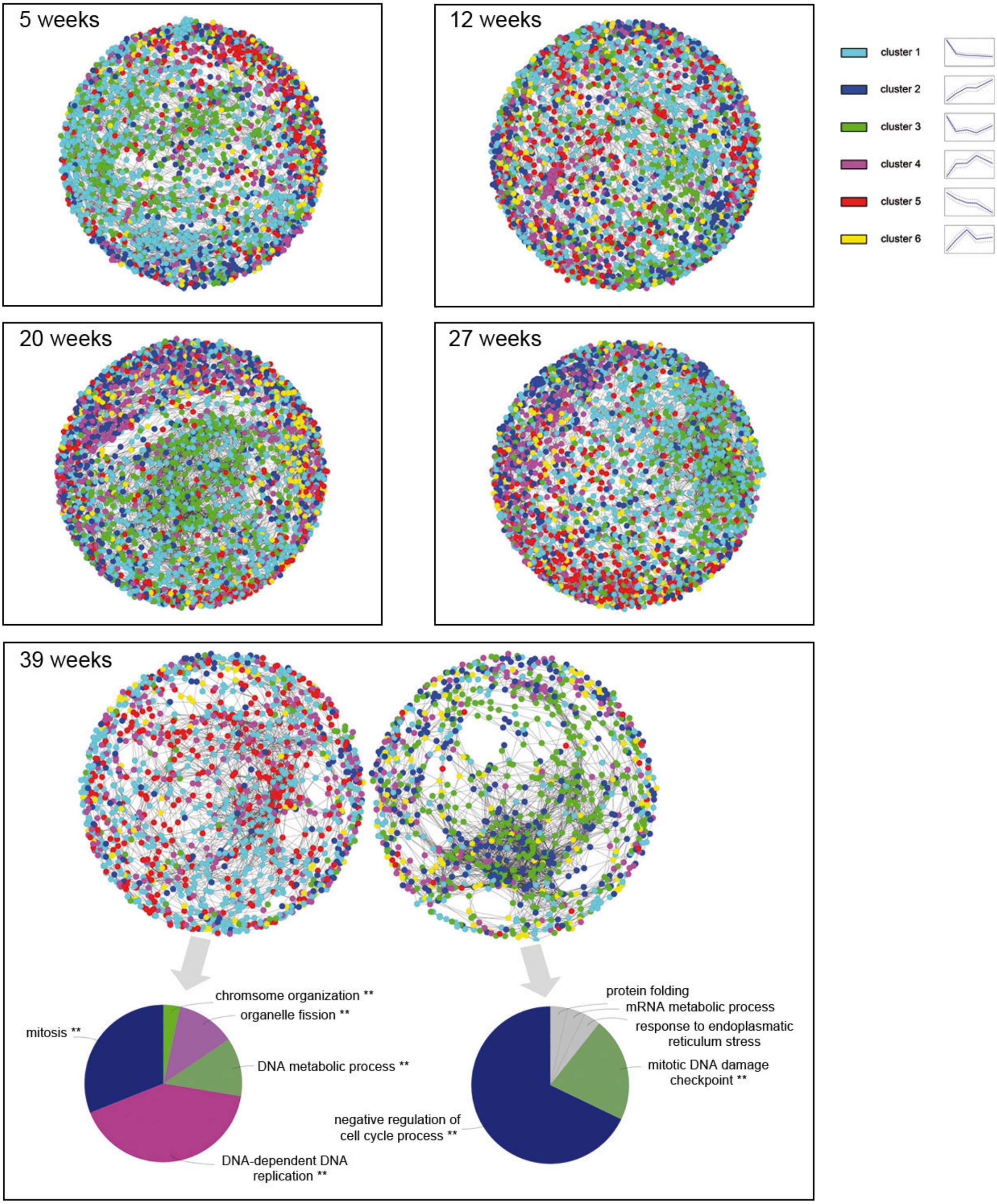

Figure S8 Correlation-based network of DEGs as a function of age calculated in Cytoscape. In the top row are shown clusters of the genes connected by Pearson´s correlation ≥ 0.99 at ages 5 (left) an 12 weeks (right), spring-embedded layout. FCM cluster membership is color-coded (upper right). Middle row, correlation-based networks at age 20 (left) and 27 weeks (right), settings as for 5 weeks. In 39 weeks genes separate in two cluster. Bottom row, GO categories (Biological Process) that are specific for the left and the right network at 39 weeks computed using ClueGO. The dimension of the wedges is proportional to the number of clustered terms. Grey wedges indicates individual terms that could not be clustered

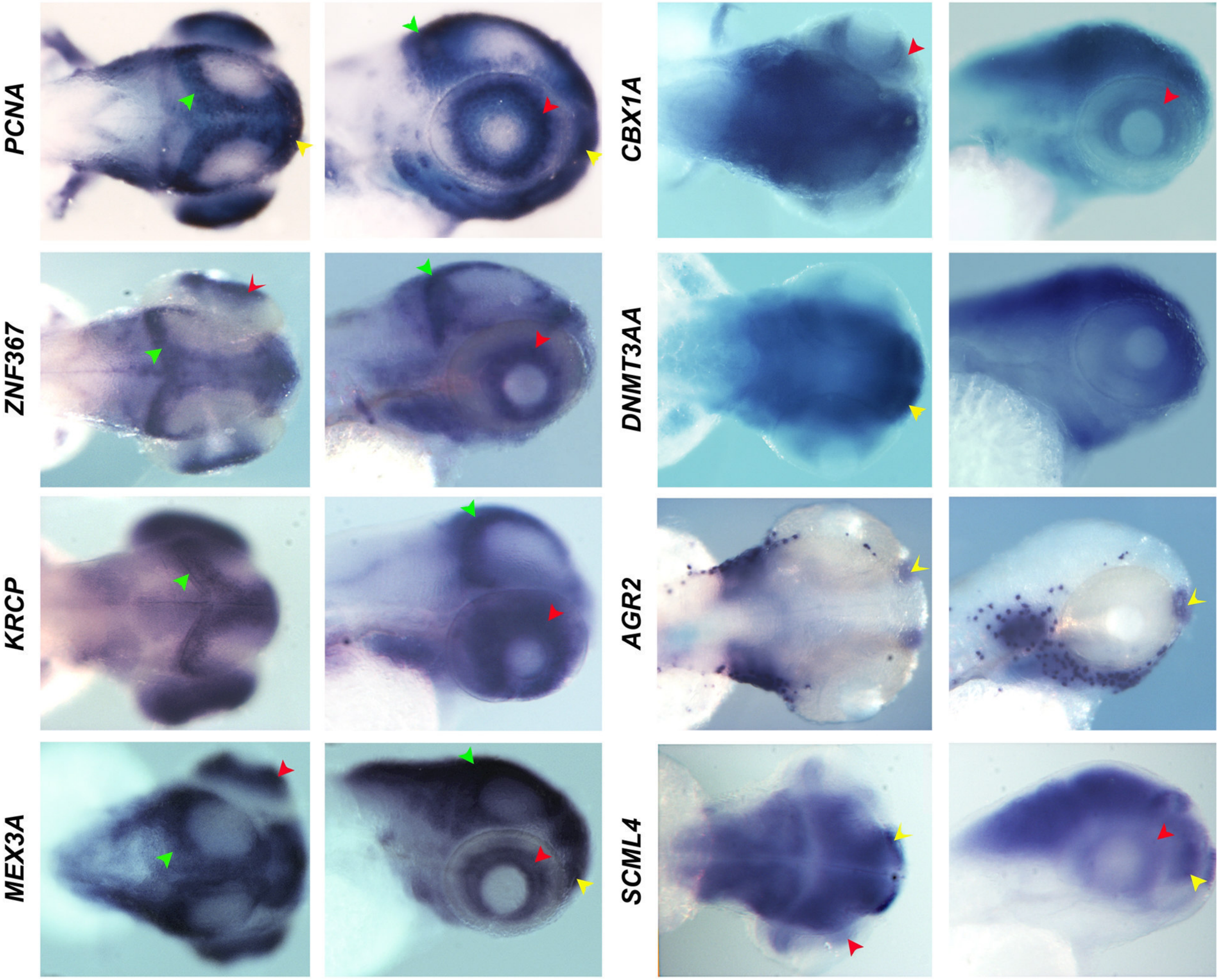

Figure S9 Dorsal and lateral view of 72hpf zebrafish embryos processed by *in situ* hybridization. *PCNA* (Proliferating Cell Nuclear Antigen) riboprobe has been used to visualize proliferative region of the zebrafish larval brain as indicated by the arrowheads. It is possible to compare the PCNA expression pattern with that of the mRNAs of the following cluster 1 DEGs: *ZNF367*, *KRCP*, *MEX3A*, *CBX1A*, *DNMT3AA*, *AGR2*, and *SCML4*. Arrowheads point to neurogenic brain regions: red arrowhead, ciliary marginal zone of the embryonic retina; green arrowhead, proliferative niche in the optic tectum; yellow arrowhead, olfactory neuroepithelium.

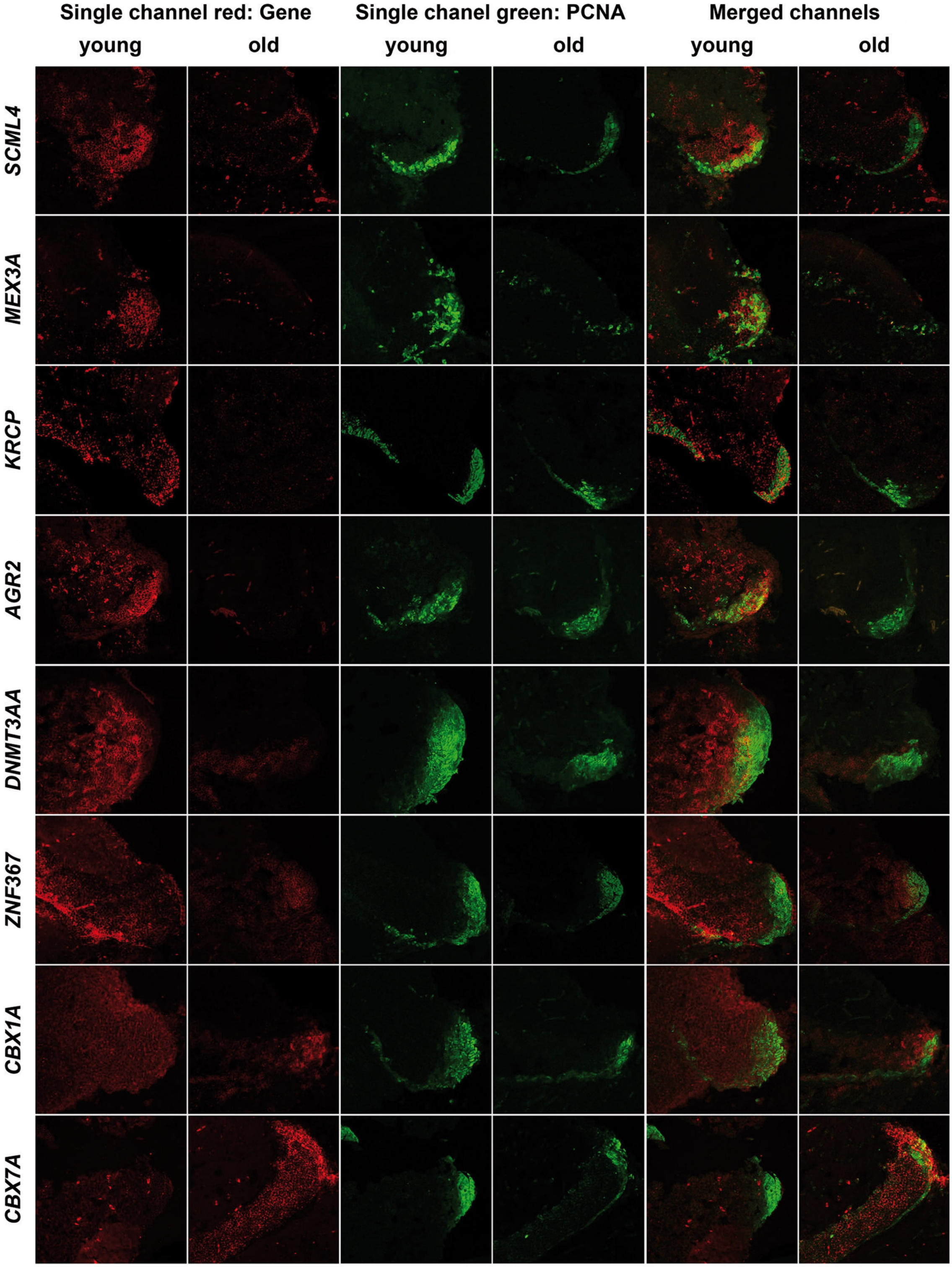

Figure S10 Double-labeling *in situ* hybridization (ISH) and immunohistochemistry (IHC) in *N. furzeri* on the posterior margin of the optic tectum (OTp) for the following genes: *SCML4*, *MEX3A*, *KRCP*, *AGR2*, *DNMT3AA*, *ZNF367*, *CBX1A*, and *CBX7A* – ISH signal was revealed using fluorescent Fast Red and is shown in red, PCNA IHC is visualized in green to localize the proliferative niche of the Otp. Red and green channels are represented separately in columns 1 and 2, 3 and 4, respectively, and merged in columns 5, 6. Columns 1, 3, 5 show the genes expression in the OTp of 6 weeks old animals; Columns 2, 4, 6 show the genes expression in the OTp of 25 weeks old animals. Images are representative of three replicates.

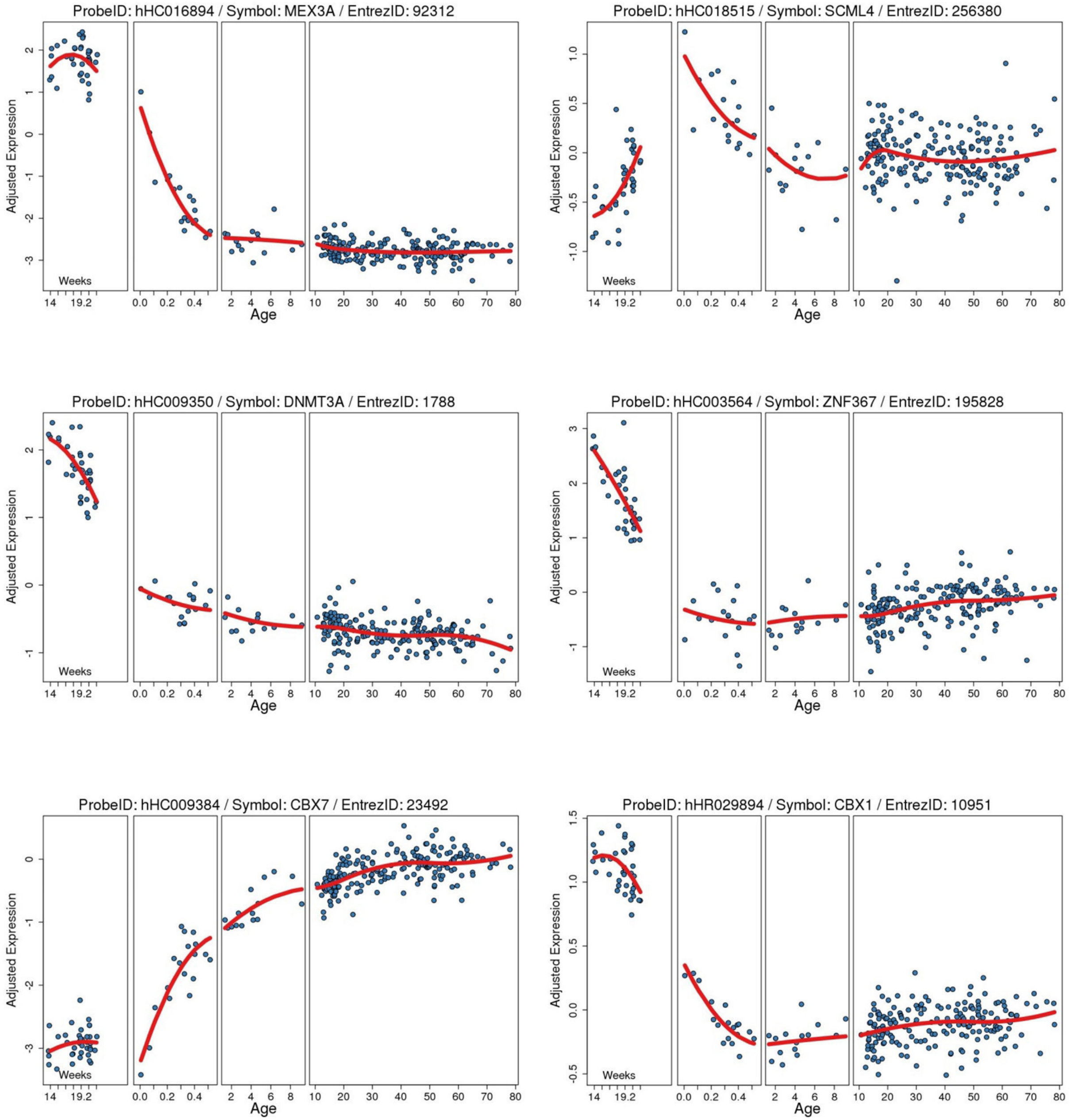

Figure S11 Expression profiles in human prefrontal cortex from 14 weeks gestation time to 100 years were downloaded from braincloud (http://braincloud.jhmi.edu/). Dark points represent individuals and the red line depicts the interpolation of the individual data

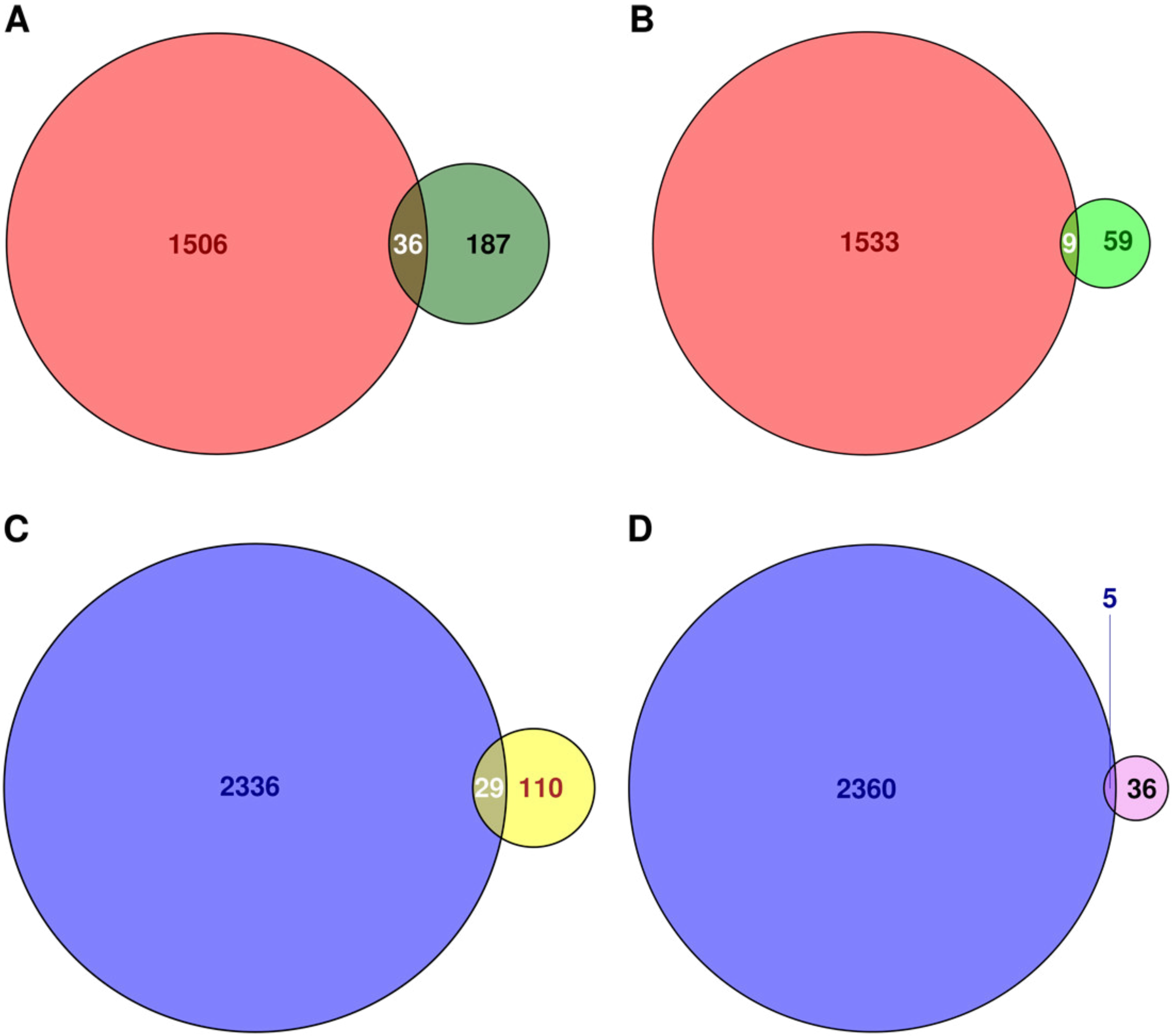

Figure S12 Intersection of DEG found in this study and in meta-analysis study (de Magalhães et al, 2009). (A, B) Up-regulated DEG (Cluster 2,4,6) vs. Table S3 and S5, respectively. (C, D) Down- regulated DEG (Cluster 1,3,5) vs. Table S4 and S6, respectively.
