# Supplementary material for: RNA-seq of the aging brain in the short-lived fish N. furzeri – conserved pathways and novel genes associated with neurogenesis
Source: Aging Cell. 2014 Jul 25;13(6):965–74. doi: 10.1111/acel.12257 (PMC4326923; doi:10.1111/acel.12257)
Supplement: Table S5 — List of down-regulated genes related to morphogen pathways. [file acel0013-0965-sd6.doc]

Table S5 List of genes in Cluster 1 with high-quality in situ hybridization in ZFIN.

| **Gene name** | **Expression in the nervous system** |
| --- | --- |
| zgc:92591 | NSC |
| abca1b | ? |
| agr2 | NSC |
| arr3 | ? |
| bora | yes |
| ccnb1 | NSC |
| ccnb2 | NSC |
| ccne2 | NSC |
| ccnf | yes |
| cdca7 | NSC |
| cenpf | NSC |
| chad | yes |
| chaf1a | NSC |
| chaf1b | yes |
| chek1 | yes |
| cnp | yes |
| col11a1a | ? |
| col1a1b | ? |
| col1a2 | ? |
| col6a2 | ? |
| col9a1 | ? |
| dbx1b | yes |
| ddost | yes |
| depdc1a | NSC |
| dla | yes |
| dld | yes |
| dlgap5 | NSC |
| dtl | NSC |
| e2f8 | NSC |
| exo1 | NSC |
| fads2 | ? |
| fbn2b | ? |
| fbxo5 | NSC |
| g2e3 | NSC |
| gins3 | yes |
| gmnn | yes |
| h2afx | NSC |
| hells | NSC |
| her12 | yes |
| hmmr | NSC |
| hoxc6a | yes |
| kera | ? |
| kif11 | NSC |
| kif15 | NSC |
| kif23 | NSC |
| kpna2 | NSC |
| krt5 | ? |
| lect1 | yes |
| matn4 | yes |
| mcm2 | NSC |
| mcm3 | NSC |
| mcm4 | NSC |
| mcm5 | NSC |
| melk | NSC |
| mibp | NSC |
| mki67 | NSC |
| naprt1 | yes |
| nasp | NSC |
| ncapd3 | yes |
| ncapg | NSC |
| ncapg2 | yes |
| ncaph | NSC |
| ndc80 | NSC |
| nes | yes |
| neurod4 | yes |
| neurog1 | yes |
| nuf2 | NSC |
| nusap1 | NSC |
| nutf2l | yes |
| ogn | ? |
| pcna | NSC |
| plk1 | NSC |
| pole | NSC |
| pole2 | yes |
| racgap1 | NSC |
| rpa1 | yes |
| rrm1 | NSC |
| rrm2 | NSC |
| rtkn2 | NSC |
| scarb2 | yes |
| serpinf1 | yes |
| si:ch211-69g19.2 | NSC |
| si:dkeyp-113d7.4 | ? |
| smc2 | NSC |
| smc4 | NSC |
| sox11a | yes |
| stmn1a | yes |
| tacc3 | NSC |
| tcf7 | yes |
| tipin | yes |
| tk1 | NSC |
| tram2 | yes |
| tuba8l3 | yes |
| tubb5 | yes |
| uhrf1 | yes |
| whsc1 | NSC |

Yes means that the genes is generically expressed in the developing nervous system, NSC indicates exression restricted to the neurogenic niches.
