# Supplementary material for: RNA-seq of the aging brain in the short-lived fish N. furzeri – conserved pathways and novel genes associated with neurogenesis
Source: Aging Cell. 2014 Jul 25;13(6):965–74. doi: 10.1111/acel.12257 (PMC4326923; doi:10.1111/acel.12257)
Supplement: Table S6 — Genes of cluster 1 with expression present in ZFIN. [file acel0013-0965-sd7.docx]

Table S6 List of primers for qPCR.

| **Gene** | ***N. furzeri* cDNA** | **Sequence** | |
| --- | --- | --- | --- |
| *AGR2* | Nofu_GRZ_cDNA_3_0070469 | F: TGAGGAGGCTCTCTTCTGG | R: CGTGGAACATACTGTCCATCTG |
| *CBX7* | Nofu_GRZ_cDNA_3_0008423 | F: AGAAAGGTCTCAGGCCCAG | R: ACAGCTGTTCAACGTCTGTG |
| *CCNB1* | Nofu_GRZ_cDNA_3_0209004 | F: CCTTGAAGGTCCTGGAAGCA | R: TGTGCCCCATAACAGGAATCA |
| *CDKN1A* | Nofu_GRZ_cDNA_3_0180519 | F: AGAAGCGAACTGCACCTGAT | R: GCAGTCCGGTACTCCATCTG |
| *CDKN2D* | Nofu_GRZ_cDNA_3_0049631 | F: GCAGCAGCTAAAGGGAACAC | R: AGCTCCTTTTTCCAACAGCA |
| *COL1A2* | Nofu_GRZ_cDNA_3_0003083 | F: TCTGGTTCGGAGAGACCATC | R: CGCTGTTCTTGCAGTGGTAG |
| *COL10A1* | Nofu_GRZ_cDNA_3_0002120 | F: CCACTGGAAAGGGGTATGTG | R: GGCAGACCAATTCCATTCTC |
| *DNMT3Aa* | Nofu_GRZ_cDNA_3_0011890 | F: GACGACTGGCCAATCAGACT | R: CGGTTTCCTCTTCTCTGCTG |
| *EEF1B2* | Nofu_GRZ_cDNA_3_0054203 | F: CTGGCCTCAAAGTGCTGAAC | R: GGCTGACAGCACATCAAAGA |
| *IGFBP1A* | Nofu_GRZ_cDNA_3_0029716 | F: AGGAGAGCATGAAAGCCAAA | R: AGGTCGGAGGAGCCTAAAAG |
| *KIAA0101* | Nofu_GRZ_cDNA_3_0200960 | F: ACGTTGCCAGGATGGTAAGA | R: GACGTGTTGGAGGAACCAGT |
| *KRCP* | Nofu_GRZ_cDNA_3_0016363 | F: CACTCTGGAGGGAGGAAACA | R: CCTCCTGATCTGCTGATCG |
| *MEX3A* | Nofu_GRZ_cDNA_3_0010775 | F: CTGCAAGGGAAGAGCAAGTT | R: CCTCGTACCGGAGTTTTGAT |
| *PVALB4* | Nofu_GRZ_cDNA_3_0054916 | F: GTGGCTTCATTGAGGAGGAG | R: TCTTGCCATCACCATCAGAG |
| *RELA* | Nofu_GRZ_cDNA_3_0066749 | F: TACAAGTGTGAGGGCCGTTC | R: TGTCACCAGAGAGATGCGAA |
| *SCML4* | Nofu_GRZ_cDNA_3_0005059 | F: GGAGGAAACACAACGCAGAG | R: CCGTCTGACTGGGGATAAAA |
| *TERT* | Nofu_GRZ_cDNA_3_0195214 | F: GTGGATGTGAGCAGTGCCTA | R: CAGGAAATCCGCCTGTCTAA |
| *TP53* | Nofu_GRZ_cDNA_3_0066717 | F: TCAGAGCCACAGCGGTTTAT | R: AACGCGGATCAAATGACTTT |
| *ZNF367* | Nofu_GRZ_cDNA_3_0177522 | F: CTCCTACCGGGACTCAGACA | R: AGGACGTTTCTCCCTCGTTT |
| *ZNF395b* | Nofu_GRZ_cDNA_3_0176905 | F: CCACTCGGAGCATCTCTATCA | R: TGAGAGTTGTTGTGGCTGGA |
| *TBP* | Nofu_GRZ_cDNA_3_0013286 | F: CGGTTGGAGGGTTTAGTCCT | R: GCAAGACGATTCTGGGTTTG |

The accession number refers to the N. furzeri transcriptome browser (https://gen100.imb-jena.de/EST2UNI/nfintb/)
