# Supplementary material for: RNA-seq of the aging brain in the short-lived fish N. furzeri – conserved pathways and novel genes associated with neurogenesis
Source: Aging Cell. 2014 Jul 25;13(6):965–74. doi: 10.1111/acel.12257 (PMC4326923; doi:10.1111/acel.12257)
Supplement: Table S7 — Primers used to generate zebra fish ISH probes. [file acel0013-0965-sd8.docx]

Table S7 sequences of primers and probes for *in situ* hybridization.

| **gene** | **Probe and primers** |
| --- | --- |
| ***ZNF-367***  ID: [**Nofu_GRZ_cDNA_3_0177522**](https://gen100.imb-jena.de/EST2UNI/nfintb/sequence.php?seq_name=Nofu_GRZ_cDNA_3_0177522) | GATGAACAGAATGATACAGGGTGTGGTCAGAGAAAGAAAATGTGTTGCTTTCCAGGTTTCATCTCCGGCGCTTTACGGACAGTTTTCCCGCTGCTGGCGAAAGCAGCGGTCATGGAAAGAAACTGAAACGTGTGAAACAGTTCTAACCTCACTGTTACTACTCTGTTTAAAAAGTGGGCAAGCAGTGATGGAGTCCGTCATCATGTGGGTCATTTTTATACGCAAATAAGAGATCATTGCAAGCCTAATCCAGATATCTGATCATGCAGACCAAAGCACTCTGGTCGGATTTTCCTGTTTAGAGATTCTTGTTTTGTGGTCAAATTTCTACTTTTAGAACATAACCTTTAATATAAAACATGCGTAAAAGATTTATTTTTGCCTAAAAACAGAAACATTTCCATACTTTTTGCTTCCATTTCCTCGAATCTTTCAGATCATTCTATACAAAATACTCAACTTTTTAAAATATCAAACTTGTTTAATTTCCACAAAGAAAATAATCCAAATGAAGCACTTACACAAACTAAATTCTAGGATTAAGCTTGCAATGCAGTTTTTGTCTGCTATATAAACCGTAGTGCTGGGTGGAT  F: TAAGATGAACAGAATGATACAGG  T7- R:GTAATACGACTCACTATAGGGAATCCACCCAGCACTACGG |
| ***AGR-2***  ID: Nofu_GRZ_cDNA_3_0070469 | TTGCTGCTCTCCTGGTTTTTGTGACCGTGTCCTCAGCTTTTGCCAAATATTTTCCAAAGACAGGAAAAAGGATTCCACAGACTCTGTCCAGAGGGTGGGGGGACCAGCTGATCTGGGCTCAGACGTATGAGGAGGCTCTCTTCTGGTCCCGATCCAAGAACAAGCCTCTGATGGTTCTGTTCCACCTGGAGGACTGCCCACACAGCCAAGCACTAAAGAAGGTGTTCTCGGAGAACAATGAGATCCAGAAAACCCTTGATGAAGACTTCATTGTCCTCAATCTGGTGTATGAAACCACAGACAAACATCTCTCTCCAGATGGACAGTATGTTCCACGAATCATTTTTGTTGACCCCACCATGACGGTGAGAGCTGACATCACTGGCCGTTACTCCAATCGCATGTACGCCTATGAAACTGGAGACATCAAACTATTGATCACCAACATGCAGAAGGCTAAAAAGCTGCTGAAGTCTGAGCTGTAAGCAGCTATCTACCAGCAGACAGAACCACCACAGACCTTCAGAATATGGTCTCCATCTTTCTGTTGAGGATGTTCATTACTTCAGGTGTA  F: TTGCTGCTCTCCTGGTTTTTG  T7-R: GTAATACGACTCACTATAGGGTACACCTGAAGTAATGAACATCCTC |
| ***SCML4***  ID: Nofu_GRZ_cDNA_3_0005059 | AGTCCAAATCTCCACCCAACAAGGACCCAACCACTTGGTCAGTGGAGGACGTCGTCTGGTTCATCAGGGATGCAGACCCTCAGGGACTCGGACCTCACGCAGACGTCTTCAGGAAACACGAGATAGACGTAAACGCTTTGTTACTCCTCAAGAGCGACATGATCATGAAGTACCTCGGACTGAAGCTCGGGCCGGCCTTGAAGCTGTGCTTCCACATCGACAAGCTAAAGCAGACGAAGTTCTGAAAGCTGTCCCTCAGAACCCGGCAGAACATTTGAACTGGGTCGGTTCCAGGTCTCCCAGCCTCACGGGGTGTGGCATTTCCTTTAGGGTTCCCAGAAACAAGGTACTTATAAATAAAAGGCAACAATTAACAGTCACTGCTTTAAAGTCAAGTGCAATGATTAAATGTAACTTAGCAGATATTACTTCAGAAATGCATCTGTAACATTACACTTTAAGCCATGAGAAGATTCACTTCTAAACATCAGGCTCTCCAACTGCAGTTTATGTAAAAACATCATTTTAAGGTAATTTTGCATCTTCATGACTAAAGGTGGAAGTTTTGGGTTTTT  F: GAGTCCAAATCTCCACCCAAC  T7-R: GTAATACGACTCACTATAGGGAAAAACCCAAAACTTCCACC |
| ***KRCP***  ID: Nofu_GRZ_cDNA_3_0016363 | ATGGAAGAGTTTGGAGTTTATGCAGTTTTTGGAGTAAACGGTCCGCCTCAAAGGCTGCTGAGTGCGGACGGGTCGTGCAGAGTTTCTGTTGCAGTTCCTCCATCCGTCCAGCTGGTGGTGGTGTTCAGCTGCGGACCGTGGGGGGAGAGGATCTGCGTGAACGCGGAGCTCAGTGATGCTGATCGGTTCCCCATCACTATAGGAAAACTCACTCCTTATAACAGATGCCTGGCCTGGGAGCAGTGGGAAGAGGAGACATGGACAGACTGTGTCACACTGAATCTCACTCTGGAGGGAGGAAACACGGACTCTGTGGTGCCCCTCGCAGCCCGAGAGCTCCACGGCAAGAGGAAGAGAGAGCGATCAGCAGATCAGGAGGTGGACGGTGGTCTAAATAAGGTGACCAAGGCGGGACAAGAAGAGAACGTGTGTCCTAATGCCAGTAAGGAGAAGCCCACGCCTGTACGAAAGGTCAGAGGTCAAAGCAGAATCAGCCAGAAGCTGTTTGCTAGTGGAGCGGATTCCTCAGAGGTGAAAGCAGCAGGTCATGAAGCAGGAGGGGAGGGGACGGCTCCTCCCCAGACTGCATCCAGGATGAAGAGTCGGCAGACCAAGACTCCCACTCAGACCACTTCTCTGATCATCCCATCGGGACGCTGGGGACAAACCTTATGTCCCATTGACTCTCAGACGG  F: ATGGAAGAGTTTGGAGTTTATG  T7-R: GTAATACGACTCACTATAGGCCGTCTGAGAGTCAATGGGAC |
| ***CBX-1***  ID: Nofu_GRZ_cDNA_3_0026839 | CGGATCCTGCTGGTCCCCAGGTCTACCCGATGAGTATGAGCCAGACTCCAGAACCCTCTAATGATGCTCCTGCTGTTACAGAAGAGGCTAAAGTGACTTCAGTGGAGAAAGAAAAGAAGCCAGACGATGTCCAGAGGAAGAAGAGGAAGAGGAGGAATACGTCGTGGAAAAGGTCCTGAATCGACGGGTGGTGAAAGGCAGGGTAGAGTACCTTCTCAAGTGGAAGGGCTTCTCTGAGGAAGATAACACTTGGGAGCCTGAAGAAAACTTAGACTGTCCAGATCTGATCGCAGAATATCTGCAGACTCATAAAACTGCTCATGAGGGCAAAAGGAAGGCAGCTGGAGAAGCAGATGGGGATGAAAATAAATCAAAAAAGAAAAAAGAAGATAATGAGAAGCTACGGGGTTTTGCTCGAGGCCTGGAGCCTGAACGAATCATCGGTGCCACAGACTCCACAGGAGAACTCATGTTCCTCATGAAATGGAAAAACTCAGATGAAGCGGATCTGGTGCCGGCGAAAGAGGCTAATGTGAAGTGTCCACAGGTGGTGATCTCCTTCTACGAAGAGAGACTGACTTGGCACTCGTACCCTTCTGAAGACGAGAAGAAAGATGATAAAAACTAAGGGTTGAGGCA  F: AGCCAGACGATGTTCCAGAGG  T7-R: GTAATACGACTCACTATAGGTTCATCCACACCCCTTCGAACC |
| ***CBX-7a***  id: Nofu_GRZ_cDNA_3_0008423 | GAAGAGAGTCAGAAAGGGTAATGTGGAGTATCTACTGAAGTGGCAGGGATGGCCCCCAGAGTACAGCACGTGGGAACCAGAGGACAACATCTTGGACCCTCTCCTGGTCCTGGCCTACGAAGAGAATCAGGAGAAGATCAGATCTTTGGCCTACCGAAAGAAAGGTCTCAGGCCCAGGAAGCTCATGCTGCGGAACATCTTTGCCATGGACCTCCGCAGTGCCAGCAAGGATTCGGAGAACGCCCCCCTAGCCTGCGTCTGTCCCTCACCAGGTCCATGAGCACAGACGTTGAACAGCTGTGCCGTCGTCCAGCTGGGAGGAGGTACAGATCGAGGATGACCAAACTAGGGTCCAAACGCTCGTCCTGCAAGCCCATCCATCTCCAGAAGAAGAAGCTGGACTCCCCGCTGAAGCACTGGGGGGAGGCCAGTGAGGAGGACAGGCCAGAGTGTGAAAGTGCTGCTGAGGAGAAATGTGAAGACAGCTGCTACGGTCATTCAGAGTGCAGCTCACCACCCTTCGTGGAGCGACAGGACT  F: GAAGAGAGTCAGAAAGGGTAA  T7-R: GTAATACGACTCACTATAGGGTCCAAGTCCTGTCGCTCCAC |
| ***Mex-3a***  id: Nofu_GRZ_cDNA_3_0161794 | GACGAAGGGACGAGGCTGCAACATCACGGAATGCGTCCCGGTGCCGAGCTCCGAACATGTGGCCGAAATAGTGGGAAGACAAGGTTGCAAGATCAAAGCCCTGCGAGCCAAGACCAACACCTACATCAAAACTCCGGTACGAGGCGAGGAGCCCGTGTTCCTGATCACGGGCCGGAAGGAAGACGTCGCTTTGGCCCGCCGTGAGATCATATCTGCCGCAGAGCACTTCTCCATGCTTCGAGCGTCTCGTAATAAGCTAGGCGTGTCCTTCAGTGGCTCTCCACCCACACCGCTTCCAGGTCAGACCACCATTCAGGTGAGAGTGCCATACCGCGTTGTGGGGCTGGTGGTGGGGCCCAAAGGCTCCACCATCAAGCGTATCCAGCAGCAGACCTGCACTTACATTGTCACTCCCAGCCGGGACCGAGACCCCGTCTTTGAGATCACCGGGTCACCGAGCAATGCCGAGCGGGCCCGCGAGGAGATCGAAGCCCACATCGCCTTTCGAACGGGAGGTCTGCATGACCACAACAACGAGAACGACTGTTTGGGTCCAAACGGCGGAAGCAGCCCAGTGAGCAGCACTGGTGGTTTGGAGAGCCGGCTACAGCAGGTGTGGGGGCTGCAGGGGGGCCAGCGCAAGCCCCTCACCAGCAGCTACCGCCAGAACTTCTCAGACACCATAGTTGGAGGGGGAAACGGAGGAGGCGGAGAGGGAGGGGGGATCTACAACAAGACCAACTTC  F: GACGAAGGGACGAGGCTG  t7-R: GTAATACGACTCACTATAGGCTGGAGAAGTTGGTCTTGTTG |
