# Supplementary material for: RNA-seq of the aging brain in the short-lived fish N. furzeri – conserved pathways and novel genes associated with neurogenesis
Source: Aging Cell. 2014 Jul 25;13(6):965–74. doi: 10.1111/acel.12257 (PMC4326923; doi:10.1111/acel.12257)
Supplement: Appendix S1 — Graphical representation of regulated KEGG pathways with rendering of the fold-changes. [file acel0013-0965-sd11.pdf]

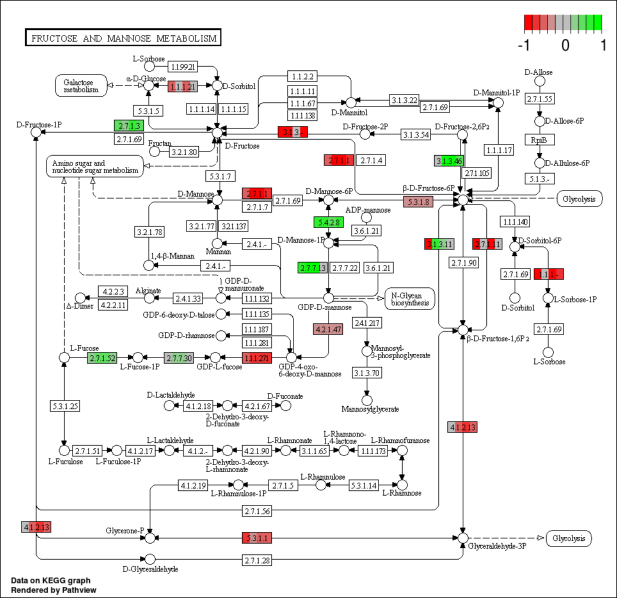
FRUCTOSE AND MANNOSE METABOLISM
-1
0
1
L-Sorbose
1.1.99.21
α-D-Glucose
Galactose metabolism
1.1.1.21
D-Sorbitol
1.1.2.2
1.1.1.11
1.1.1.67
1.1.1.138
D-Mannitol
3.1.3.22
2.7.1.69
D-Mannitol-1P
D-Allose
2.7.1.55
D-Allose-6P
RpiB
D-Allulose-6P
5.1.3.-
5.3.1.5
1.1.1.14
1.1.1.15
D-Fructose-1P
2.7.1.3
2.7.1.69
3.1.3.-
D-Fructose-2P
3.1.3.54
D-Fructose-2,6P2
1.1.1.17
Fructan
3.2.1.80
D-Fructose
Amino sugar and nucleotide sugar metabolism
2.7.1.1
2.7.1.4
3.1.3.46
2.7.1.105
5.3.1.7
D-Mannose
2.7.1.1
2.7.1.7
2.7.1.69
D-Mannose-6P
5.3.1.8
β-D-Fructose-6P
Glycolysis
ADP-mannose
5.4.2.8
3.6.1.21
1.1.1.140
3.2.1.78
3.2.1.77
3.2.1.137
Mannan
2.4.1.-
D-Mannose-1P
3.1.3.11
2.7.1.11
D-Sorbitol-6P
1,4-β-Mannan
2.4.1.-
2.7.7.13
2.7.7.22
3.6.1.21
2.7.1.90
2.7.1.69
1.1.1.-
GDP-D-mannuronate
4.2.2.3
4.2.2.11
Alginate
2.4.1.33
1.1.1.132
Δ-Dimer
N-Glycan biosynthesis
D-Sorbitol
L-Sorbose-1P
GDP-D-mannose
GDP-6-deoxy-D-talose
1.1.1.135
2.4.1.217
GDP-D-rhamnose
1.1.1.187
1.1.1.281
4.2.1.47
2.7.1.69
β-D-Fructose-1,6P2
Mannosyl-3-phosphoglycerate
L-Fucose
2.7.1.52
L-Fucose-1P
2.7.7.30
GDP-L-fucose
1.1.1.271
GDP-4-oxo-6-deoxy-D-mannose
3.1.3.70
L-Sorbose
Mannosylglycerate
5.3.1.25
D-Lactaldehyde
4.1.2.18
2-Dehydro-3-deoxy-D-fuconate
4.2.1.67
D-Fuconate
4.1.2.13
L-Lactaldehyde
L-Rhamnonate
L-Rhamnono-1,4-lactone
L-Rhamnofuranose
L-Fuculose
2.7.1.51
L-Fuculose-1P
4.1.2.17
4.1.2.-
2-Dehydro-3-deoxy-L-rhamnonate
4.2.1.90
3.1.1.65
1.1.1.173
L-Rhamnulose
4.1.2.19
L-Rhamnulose-1P
2.7.1.5
5.3.1.14
L-Rhamnose
2.7.1.56
4.1.2.13
Glycerone-P
5.3.1.1
Glyceraldehyde-3P
Glycolysis
D-Glyceraldehyde
2.7.1.28
Data on KEGG graph
Rendered by Pathview

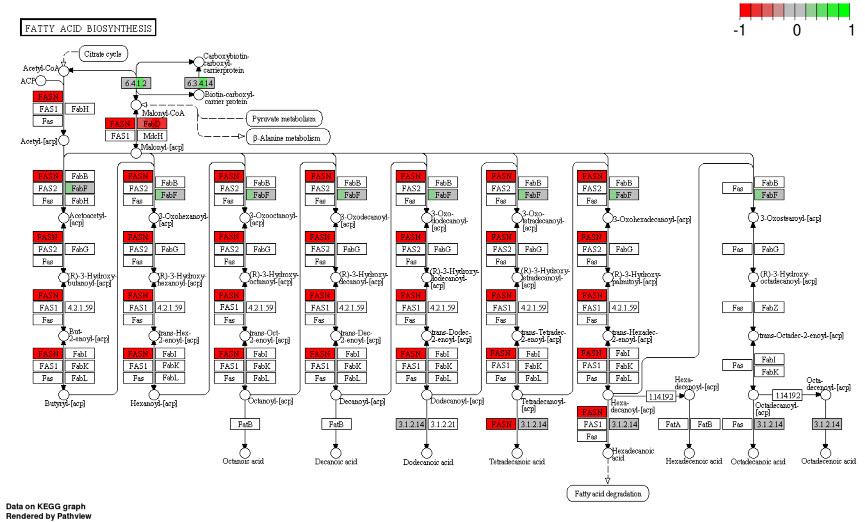
FATTY ACID BIOSYNTHESIS
-1
0
1
Citrate cycle
Acetyl-CoA
ACP
Carboxybiotin-carboxyl-carrierprotein
Biotin-carboxyl-carrier protein
6.4.1.2
6.3.4.14
Malonyl-CoA
Pyruvate metabolism
β-Alanine metabolism
FASN
FAS1
FabH
Fas
FabD
MdcH
Acetyl-[acp]
Malonyl-[acp]
FAS2
FabB
FabF
FabG
FabI
FabK
FabL
FabZ
4.2.1.59
Acetoacetyl-[acp]
3-Oxohexanoyl-[acp]
3-Oxooctanoyl-[acp]
3-Oxodecanoyl-[acp]
3-Oxo-dodecanoyl-[acp]
3-Oxo-tetradecanoyl-[acp]
3-Oxohexadecanoyl-[acp]
3-Oxostearoyl-[acp]
(R)-3-Hydroxy-butanoyl-[acp]
(R)-3-Hydroxy-hexanoyl-[acp]
(R)-3-Hydroxy-octanoyl-[acp]
(R)-3-Hydroxy-decanoyl-[acp]
(R)-3-Hydroxy-dodecanoyl-[acp]
(R)-3-Hydroxy-tetradecanoyl-[acp]
(R)-3-Hydroxy-palmitoyl-[acp]
(R)-3-Hydroxy-octadecanoyl-[acp]
But-2-enoyl-[acp]
trans-Hex-2-enoyl-[acp]
trans-Oct-2-enoyl-[acp]
trans-Dec-2-enoyl-[acp]
trans-Dodec-2-enoyl-[acp]
trans-Tetradec-2-enoyl-[acp]
trans-Hexadec-2-enoyl-[acp]
trans-Octadec-2-enoyl-[acp]
Butyryl-[acp]
Hexanoyl-[acp]
Octanoyl-[acp]
Decanoyl-[acp]
Dodecanoyl-[acp]
Tetradecanoyl-[acp]
Hexa-decanoyl-[acp]
Hexa-decenoyl-[acp]
Octadecanoyl-[acp]
Octa-decenoyl-[acp]
1.14.19.2
FatA
FatB
3.1.2.14
3.1.2.21
Octanoic acid
Decanoic acid
Dodecanoic acid
Tetradecanoic acid
Hexadecanoic acid
Hexadecenoic acid
Octadecanoic acid
Octadecenoic acid
Fatty acid degradation
Data on KEGG graph
Rendered by Pathview

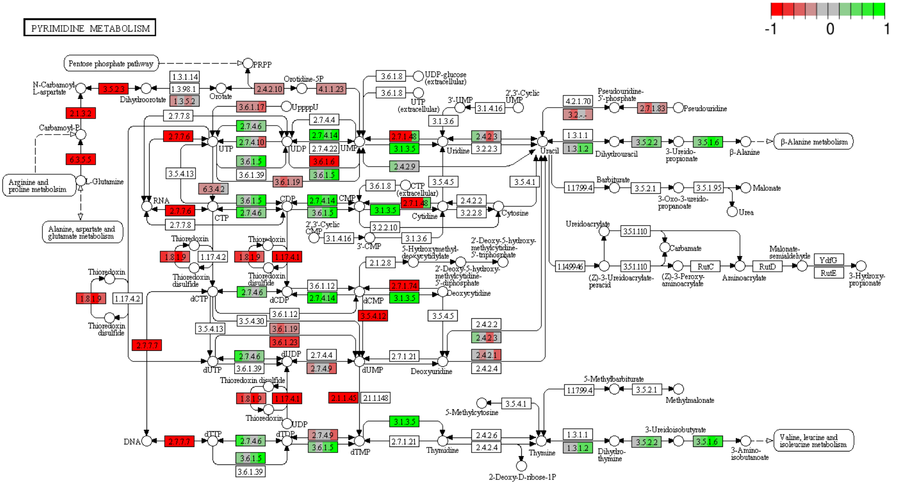

Data on KEGG graph
Rendered by Pathview

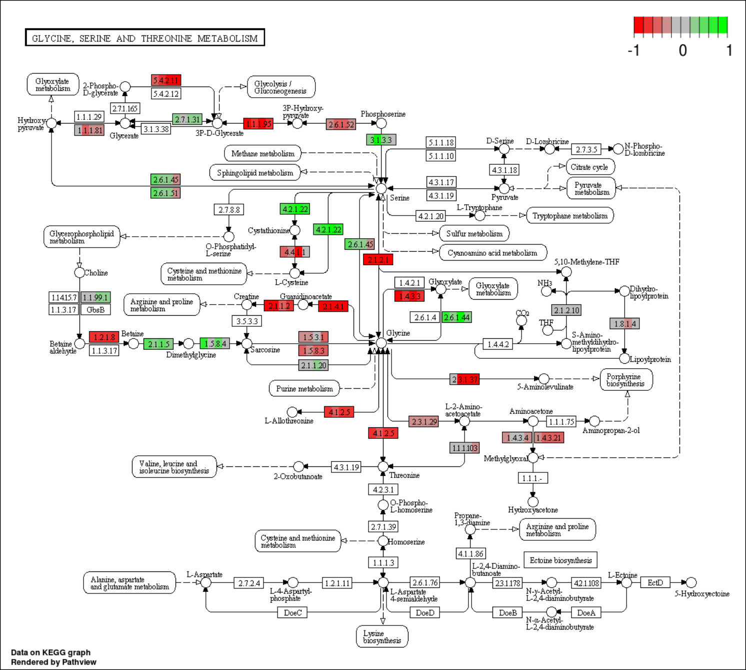
GLYCINE, SERINE AND THREONINE METABOLISM
-1
0
1
Glyoxylate metabolism
2-Phospho-D-glycerate
5.4.2.11
5.4.2.12
Glycolysis / Gluconeogenesis
2.7.1.165
Hydroxypyruvate
1.1.1.29
1.1.1.81
Glycerate
3.1.3.38
2.7.1.31
3P-D-Glycerate
1.1.1.95
3P-Hydroxypyruvate
2.6.1.52
Phosphoserine
3.1.3.3
Methane metabolism
Sphingolipid metabolism
2.6.1.45
2.6.1.51
5.1.1.18
5.1.1.10
D-Serine
D-Lombricine
2.7.3.5
N-Phospho-D-lombricine
4.3.1.18
Citrate cycle
Pyruvate metabolism
4.3.1.17
4.3.1.19
Serine
Pyruvate
4.2.1.20
L-Tryptophane
Tryptophane metabolism
2.7.8.8
4.2.1.22
Cystathionine
4.2.1.22
Glycerophospholipid metabolism
O-Phosphatidyl-L-serine
2.6.1.45
Sulfur metabolism
4.4.1.1
Cyanoamino acid metabolism
2.1.2.1
5,10-Methylene-THF
Choline
Cysteine and methionine metabolism
L-Cysteine
1.4.2.1
Glyoxylate
Glyoxylate metabolism
NH3
Dihydrolipoylprotein
1.1.99.1
1.1.1.8.1
1.14.15.7
1.1.3.17
GbsB
Creatine
Guanidinoacetate
Arginine and proline metabolism
2.1.1.2
2.1.4.1
1.4.3.3
2.6.1.4
2.6.1.44
CO2
2.1.2.10
THF
1.8.1.4
3.5.3.3
Betaine aldehyde
1.2.1.8
1.1.3.17
Betaine
2.1.1.5
Dimethylglycine
1.5.8.4
Sarcosine
1.5.3.1
1.5.8.3
2.1.1.20
Glycine
1.4.4.2
S-Aminomethyldihydrolipoylprotein
Lipoylprotein
Purine metabolism
2.3.1.37
5-Aminolevulinate
Porphyrine biosynthesis
L-Allothreonine
4.1.2.5
L-2-Amino-acetoacetate
2.3.1.29
Aminoacetone
1.1.1.75
Aminopropan-2-ol
4.1.2.5
1.4.3.4
1.4.3.21
1.1.1.103
Methylglyoxal
Valine, leucine and isoleucine biosynthesis
2-Oxobutanoate
4.3.1.19
Threonine
1.1.1.-
4.2.3.1
Hydroxyacetone
O-Phospho-L-homoserine
Propane-1,3-diamine
2.7.1.39
Arginine and proline metabolism
Cysteine and methionine metabolism
Homoserine
4.1.1.86
Ectoine biosynthesis
1.1.1.3
L-2,4-Diamino-butanoate
Alanine, aspartate and glutamate metabolism
L-Aspartate
2.7.2.4
L-4-Aspartyl-phosphate
1.2.1.11
L-Aspartate 4-semialdehyde
2.6.1.76
2.3.1.178
N-γ-Acetyl-L-2,4-diaminobutyrate
4.2.1.108
L-Ectoine
EctD
5-Hydroxyectoine
DoeC
DoeD
DoeB
N-α-Acetyl-L-2,4-diaminobutyrate
DoeA
Lysine biosynthesis
Data on KEGG graph
Rendered by Pathview

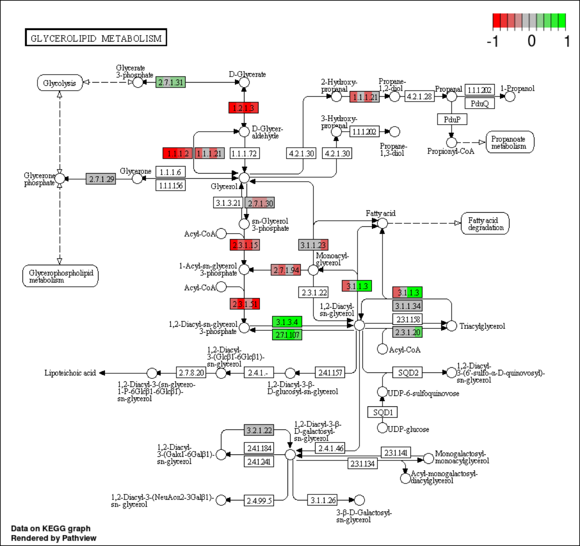

GLYCEROLIPID METABOLISM
-1 0 1
Glycolysis
Glycerate 3-phosphate
2.7.1.31
D-Glycerate
1.2.1.3
D-Glyceraldehyde
2-Hydroxy-propanal
1.1.1.21
Propane-1,2-diol
4.2.1.28
Propanal
1.1.1.202
PduQ
1-Propanol
PduP
Propionyl-CoA
Propanoate metabolism
3-Hydroxy-propanal
1.1.1.202
Propane-1,3-diol
1.1.1.2
1.1.1.21
1.1.1.72
4.2.1.30
4.2.1.30
Glycerone phosphate
2.7.1.29
Glycerone
1.1.1.6
1.1.1.156
Glycerol
3.1.3.21
2.7.1.30
sn-Glycerol 3-phosphate
Acyl-CoA
2.3.1.15
3.1.1.23
Fatty acid
Fatty acid degradation
1-Acyl-sn-glycerol 3-phosphate
2.7.1.94
Monoacylglycerol
Acyl-CoA
2.3.1.51
3.1.1.3
3.1.1.3
2.3.1.22
3.1.1.34
1,2-Diacyl-sn-glycerol
2.3.1.158
1,2-Diacyl-sn-glycerol 3-phosphate
3.1.3.4
2.7.1.107
2.3.1.20
Triacylglycerol
Acyl-CoA
Glycerophospholipid metabolism
1,2-Diacyl-3-(Glcβ1-6Glcβ1)-sn-glycerol
Lipoteichoic acid
2.7.8.20
2.4.1.-
2.4.1.157
1,2-Diacyl-3-(sn-glycero-1-P-6Glcβ1-6Glcβ1)-sn-glycerol
1,2-Diacyl-3-β-D-glucosyl-sn-glycerol
SQD2
1,2-Diacyl-3-(6'-sulfo-α-D-quinovosyl)-sn-glycerol
UDP-6-sulfoquinovose
SQD1
UDP-glucose
3.2.1.22
1,2-Diacyl-3-β-D-galactosyl-sn-glycerol
1,2-Diacyl-3-(Galα1-6Galβ1)-sn-glycerol
2.4.1.184
2.4.1.241
2.4.1.46
2.3.1.141
Monogalactosyl-monoacylglycerol
2.3.1.134
Acyl-monogalactosyl-diacylglycerol
1,2-Diacyl-3-(NeuAcα2-3Galβ1)-sn- glycerol
2.4.99.5
3.1.1.26
3-β-D-Galactosyl-sn-glycerol
Data on KEGG graph
Rendered by Pathview

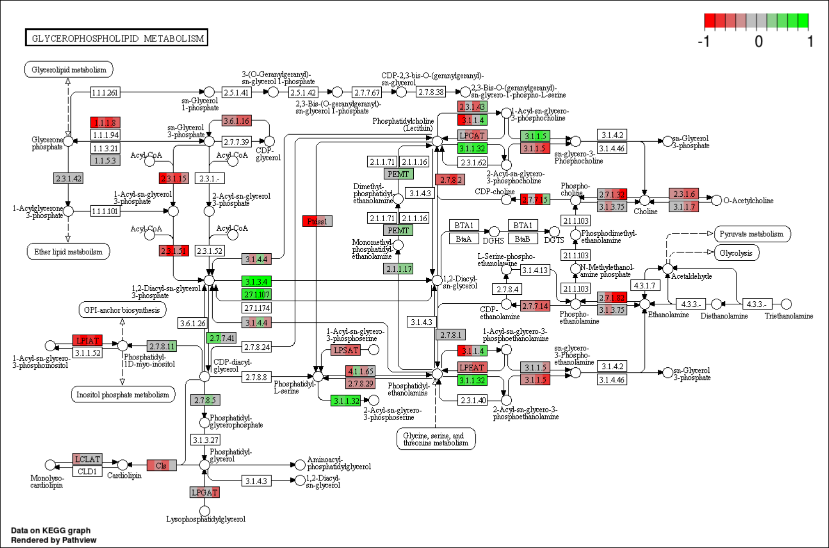

GLYCEROPHOSPHOLIPID METABOLISM
-1
0
1
Glycerolipid metabolism
1.1.1.261
sn-Glycerol 1-phosphate
2.5.1.41
3-(O-Geranylgeranyl)-sn-glycerol 1-phosphate
2.5.1.42
2,3-Bis-(O-geranylgeranyl)-sn-glycerol 1-phosphate
2.7.7.67
CDP-2,3-bis-O-(geranylgeranyl)-sn-glycerol
2.7.8.38
2,3-Bis-(O-geranylgeranyl)-sn-glycero-1-phospho-L-serine
1.1.1.8
1.1.1.94
1.1.3.21
1.1.5.3
Glycerone phosphate
sn-Glycerol 3-phosphate
3.6.1.16
2.7.7.39
CDP-glycerol
Acyl-CoA
2.3.1.42
2.3.1.15
2.3.1.-
1-Acylglycerone 3-phosphate
1.1.1.101
1-Acyl-sn-glycerol 3-phosphate
2-Acyl-sn-glycerol 3-phosphate
Ether lipid metaboilsm
2.3.1.51
2.3.1.52
1,2-Diacyl-sn-glycerol 3-phosphate
3.1.4.4
3.1.3.4
2.7.1.107
2.7.1.174
GPI-anchor biosynthesis
3.6.1.26
LPIAT
3.1.1.52
2.7.8.11
2.7.7.41
2.7.8.24
1-Acyl-sn-glycero-3-phosphoinositol
Phosphatidyl-1D-myo-inositol
CDP-diacylglycerol
2.7.8.8
Inositol phosphate metabolism
2.7.8.5
Phosphatidylglycerophosphate
3.1.3.27
Phosphatidylglycerol
LCLAT
CLD1
Cls
Monolysocardiolipin
Cardiolipin
LPGAT
Lysophosphatidylglycerol
Aminoacylphosphatidylglycerol
3.1.4.3
1,2-Diacyl-sn-glycerol
Ptdss1
Phosphatidylcholine (Lecithin)
2.3.1.43
3.1.1.4
LPCAT
3.1.1.32
2.3.1.62
1-Acyl-sn-glycero-3-phosphocholine
2-Acyl-sn-glycero-3-phosphocholine
3.1.1.5
sn-glycero-3-Phosphocholine
3.1.4.2
3.1.4.46
sn-Glycerol 3-phosphate
2.1.1.71
2.1.1.16
PEMT
2.7.8.2
Dimethylphosphatidylethanolamine
3.1.4.3
CDP-choline
2.7.7.15
Phosphocholine
2.7.1.32
3.1.3.75
2.3.1.6
3.1.1.7
Choline
O-Acetylcholine
Monomethylphosphatidylethanolamine
BTA1
BtaA
BtaB
DGHS
DGTS
2.1.1.103
Phosphodimethylethanolamine
Pyruvate metabolism
Glycolysis
2.1.1.17
1,2-Diacyl-sn-glycerol
L-Serine-phosphoethanolamine
3.1.4.13
N-Methylethanolamine phosphate
Acetaldehyde
2.7.8.4
CDP-ethanolamine
2.7.7.14
Phosphoethanolamine
2.7.1.82
4.3.1.7
4.3.3.-
Ethanolamine
Diethanolamine
Triethanolamine
2.7.8.1
1-Acyl-sn-glycero-3-phosphoserine
LPSAT
1-Acyl-sn-glycero-3-phosphoethanolamine
LPEAT
4.1.1.65
2.7.8.29
Phosphatidyl-L-serine
Phosphatidylethanolamine
2.3.1.40
sn-glycero-3-Phosphoethanolamine
2-Acyl-sn-glycero-3-phosphoserine
2-Acyl-sn-glycero-3-phosphoethanolamine
Glycine, serine, and threonine metabolism
Data on KEGG graph
Rendered by Pathview

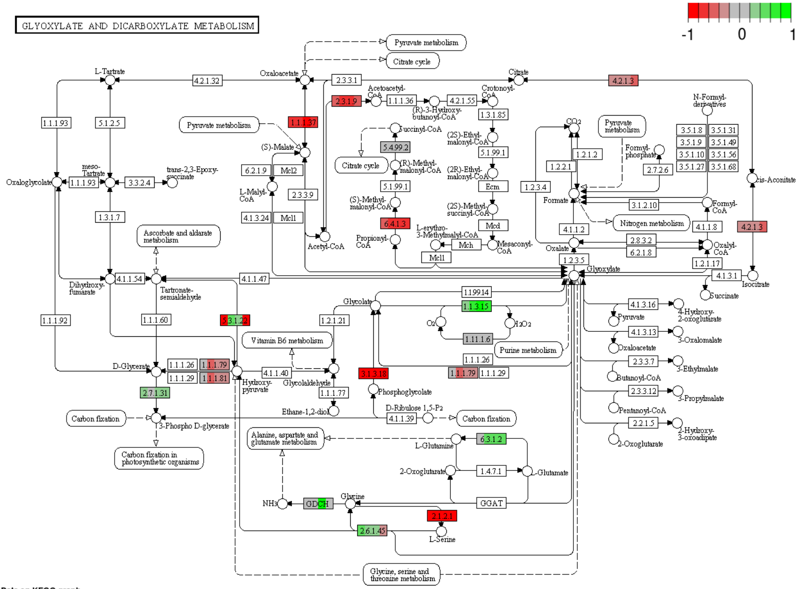

Data on KEGG graph
Rendered by Pathview

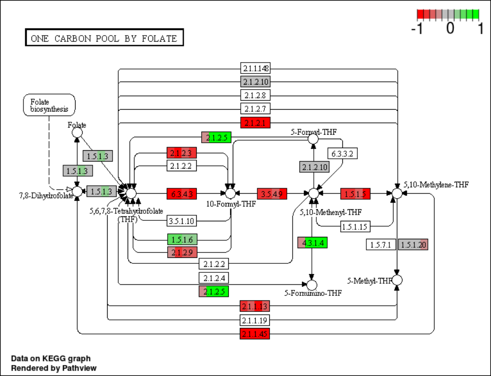

-1 0 1
ONE CARBON POOL BY FOLATE
2.1.1.148
2.1.2.10
2.1.2.8
2.1.2.7
2.1.2.1
Folate biosynthesis
Folate
1.5.1.3
1.5.1.3
7,8-Dihydrofolate
1.5.1.3
5,6,7,8-Tetrahydrofolate (THF)
2.1.2.5
2.1.2.3
2.1.2.2
6.3.4.3
10-Formyl-THF
3.5.4.9
5-Formyl-THF
6.3.3.2
2.1.2.10
1.5.1.5
5,10-Methylene-THF
5,10-Methenyl-THF
1.5.1.15
3.5.1.10
1.5.1.6
2.1.2.9
4.3.1.4
1.5.7.1
1.5.1.20
2.1.2.2
2.1.2.4
2.1.2.5
5-Formimino-THF
5-Methyl-THF
2.1.1.13
2.1.1.19
2.1.1.45
Data on KEGG graph
Rendered by Pathview

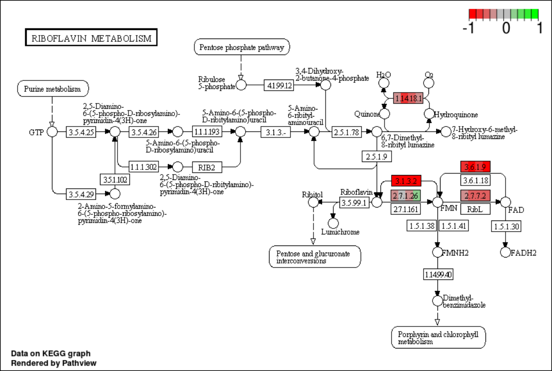
-1 0 1
RIBOFLAVIN METABOLISM
Pentose phosphate pathway
Purine metabolism
Ribulose 5-phosphate
4.1.99.12
3,4-Dihydroxy-2-butanone-4-phosphate
H2O
O2
1.14.18.1
Quinone
Hydroquinone
GTP
3.5.4.25
2,5-Diamino-6-(5-phospho-D-ribosylamino)-pyrimidin-4(3H)-one
3.5.4.26
5-Amino-6-(5-phospho-D-ribosylamino)uracil
1.1.1.193
5-Amino-6-(5-phospho-D-ribitylamino)uracil
3.1.3.-
5-Amino-6-ribityl-aminouracil
2.5.1.78
6,7-Dimethyl-8-ribityl lumazine
7-Hydroxy-6-methyl-8-ribityl lumazine
2.5.1.9
1.1.1.302
RIB2
2,5-Diamino-6-(5-phospho-D-ribitylamino)-pyrimidin-4(3H)-one
3.5.1.102
3.5.4.29
2-Amino-5-formylamino-6-(5-phospho-ribosylamino)-pyrimidin-4(3H)-one
Ribitol
Riboflavin
3.5.99.1
Lumichrome
3.1.3.2
2.7.1.26
2.7.1.161
3.6.1.9
3.6.1.18
2.7.7.2
RibL
FMN
FAD
1.5.1.38
1.5.1.41
1.5.1.30
FMNH2
FADH2
1.14.99.40
Dimethyl-benzimidazole
Pentose and glucuronate interconversions
Porphyrin and chlorophyll metabolism
Data on KEGG graph
Rendered by Pathview

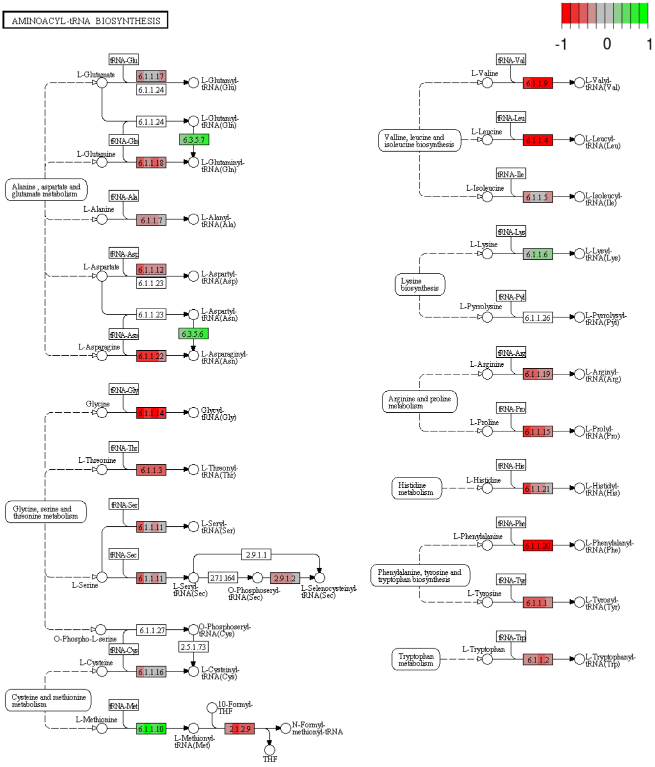

Data on KEGG graph
Rendered by Pathview

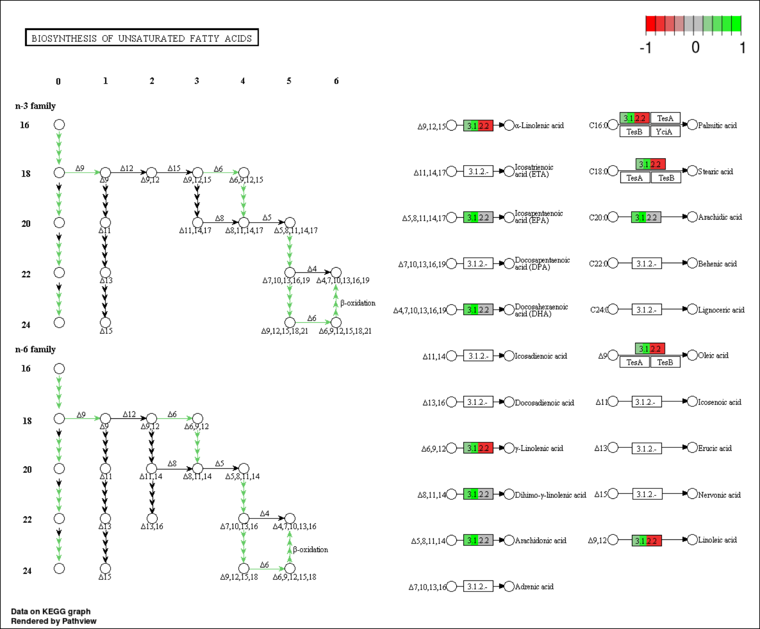
BIOSYNTHESIS OF UNSATURATED FATTY ACIDS
-1
0
1
0
1
2
3
4
5
6
n-3 family
16
18
20
22
24
Δ9
Δ12
Δ15
Δ6
Δ9
Δ9,12
Δ9,12,15
Δ6,9,12,15
Δ8
Δ5
Δ11
Δ11,14,17
Δ8,11,14,17
Δ5,8,11,14,17
Δ4
Δ13
Δ7,10,13,16,19
Δ4,7,10,13,16,19
β-oxidation
Δ6
Δ15
Δ9,12,15,18,21
Δ6,9,12,15,18,21
n-6 family
16
18
20
22
24
Δ9
Δ12
Δ6
Δ9
Δ9,12
Δ6,9,12
Δ8
Δ5
Δ11
Δ11,14
Δ8,11,14
Δ5,8,11,14
Δ4
Δ13
Δ13,16
Δ7,10,13,16
Δ4,7,10,13,16
β-oxidation
Δ6
Δ15
Δ9,12,15,18
Δ6,9,12,15,18
Δ9,12,15
3.1.2.2
α-Linolenic acid
Δ11,14,17
3.1.2.-
Icosatrienoic acid (ETA)
Δ5,8,11,14,17
3.1.2.2
Icosapentaenoic acid (EPA)
Δ7,10,13,16,19
3.1.2.-
Docosapentaenoic acid (DPA)
Δ4,7,10,13,16,19
3.1.2.2
Docosahexaenoic acid (DHA)
Δ11,14
3.1.2.-
Icosadienoic acid
Δ13,16
3.1.2.-
Docosadienoic acid
Δ6,9,12
3.1.2.2
γ-Linolenic acid
Δ8,11,14
3.1.2.2
Dihimo-γ-linolenic acid
Δ5,8,11,14
3.1.2.2
Arachidonic acid
Δ7,10,13,16
3.1.2.-
Adrenic acid
C16:0
3.1.2.2
TesA
TesB
YciA
Palmitic acid
C18:0
3.1.2.2
TesA
TesB
Stearic acid
C20:0
3.1.2.2
Arachidic acid
C22:0
3.1.2.-
Behenic acid
C24:0
3.1.2.-
Lignoceric acid
Δ9
3.1.2.2
TesA
TesB
Oleic acid
Δ11
3.1.2.-
Icosenoic acid
Δ13
3.1.2.-
Erucic acid
Δ15
3.1.2.-
Nervonic acid
Δ9,12
3.1.2.2
Linoleic acid
Data on KEGG graph
Rendered by Pathview

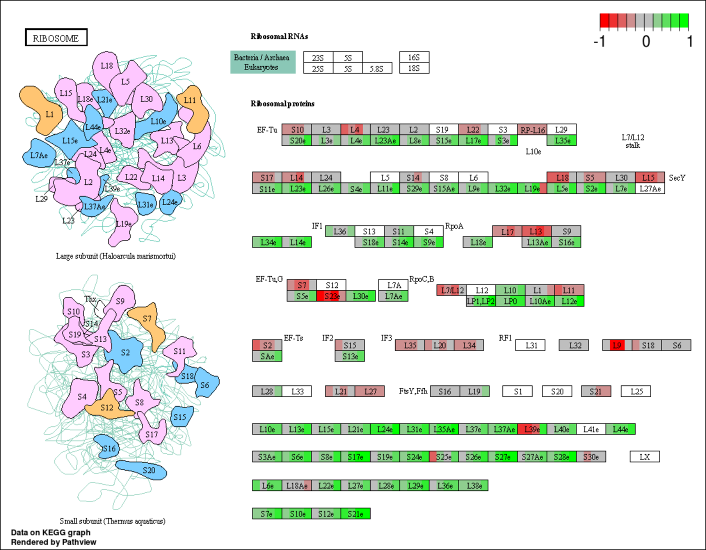
RIBOSOME
Ribosomal RNAs
Bacteria / Archaea
Eukaryotes
23S
5S
16S
25S
5S
5.8S
18S
Ribosomal proteins
-1
0
1
EF-Tu
L7/L12 stalk
L10e
SecY
IF1
RpoA
EF-Tu,G
RpoC,B
EF-Ts
IF2
IF3
RF1
FtsY,Ffh
Large subunit (Haloarcula marismortui)
Small subunit (Thermus aquaticus)
Data on KEGG graph
Rendered by Pathview

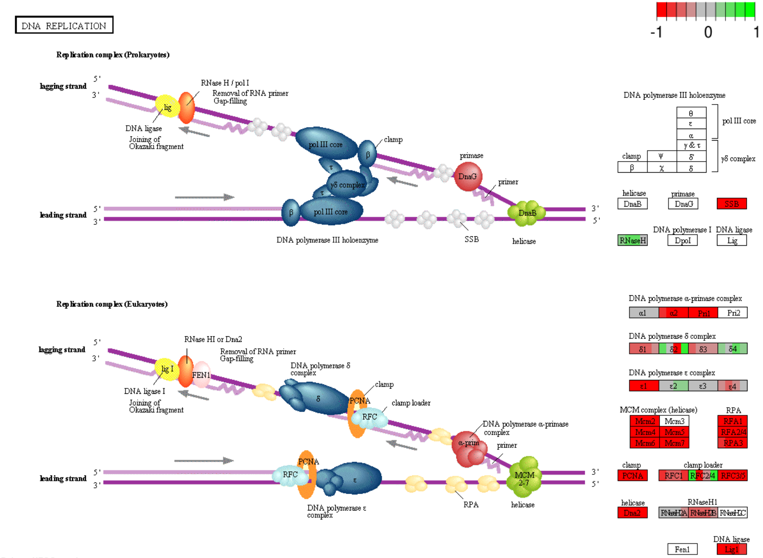

Data on KEGG graph
Rendered by Pathview

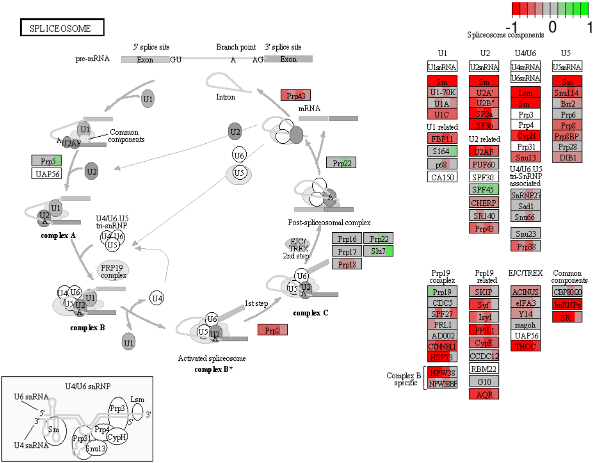

Data on KEGG graph
Rendered by Pathview

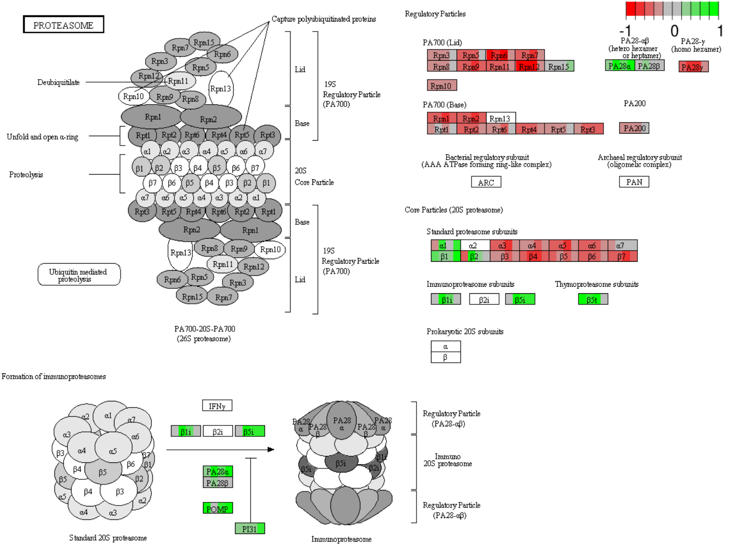

Data on KEGG graph
Rendered by Pathview

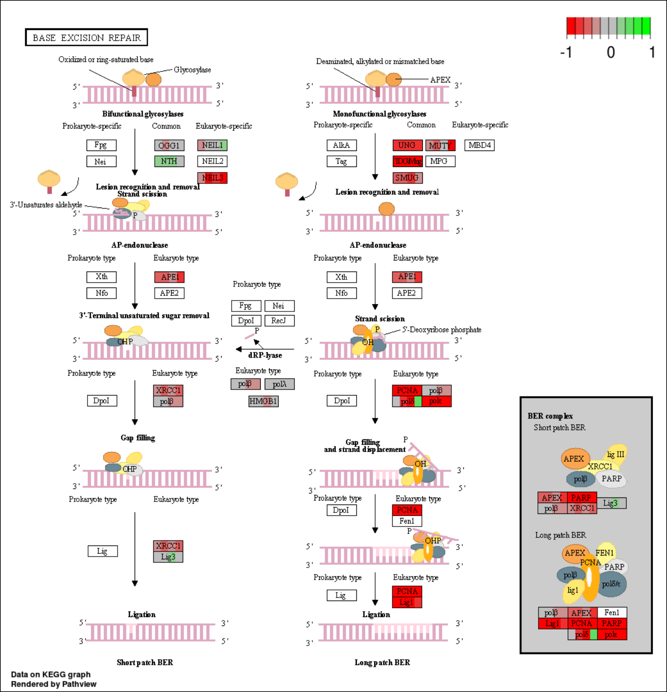

BASE EXCISION REPAIR
-1
0
1
Oxidized or ring-saturated base
Glycosylase
Bifunctional glycosylases
Prokaryote-specific
Common
Eukaryote-specific
Fpg
Nei
OGG1
NTH
NEIL1
NEIL2
NEIL3
Lesion recognition and removal
Strand scission
3'-Unsaturates aldehyde
P
AP-endonuclease
Prokaryote type
Eukaryote type
Xth
Nfo
APE1
APE2
3'-Terminal unsaturated sugar removal
OHP
DpoI
XRCC1
polβ
Gap filling
Lig
Lig3
Ligation
Short patch BER
Prokaryote type
Fpg
Nei
DpoI
RecJ
dRP-lyase
Eukaryote type
polβ
polλ
HMGB1
Deaminated, alkylated or mismatched base
APEX
Monofunctional glycosylases
AlkA
Tag
UNG
MUTY
TDG/Mbd4
MPG
SMUG
MBD4
Lesion recognition and removal
Strand scission
5'-Deoxyribose phosphate
OH
PCNA
polδ
polε
Gap filling and strand displacement
Fen1
Lig1
Long patch BER
BER complex
Short patch BER
APEX
lig III
XRCC1
polβ
PARP
Lig3
Long patch BER
FEN1
PCNA
polδ/ε
lig1
Data on KEGG graph
Rendered by Pathview

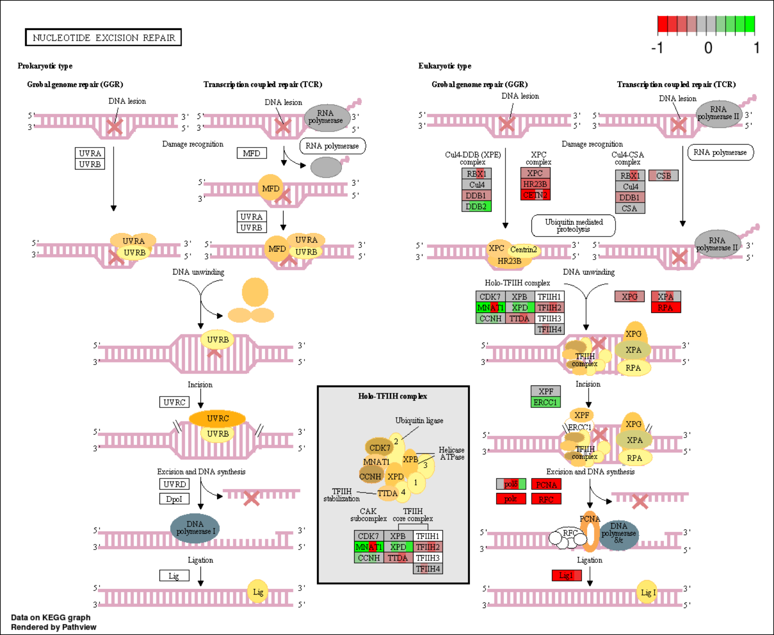
NUCLEOTIDE EXCISION REPAIR
-1
0
1
Prokaryotic type
Grobal genome repair (GGR)
Transcription coupled repair (TCR)
DNA lesion
UVRA
UVRB
Damage recognition
MFD
RNA polymerase
DNA unwinding
Incision
UVRC
Excision and DNA synthesis
UVRD
DpoI
DNA polymerase I
Ligation
Lig
Eukaryotic type
Cul4-DDB (XPE) complex
RBX1
Cul4
DDB1
DDB2
XPC complex
XPC
HR23B
CETN2
Cul4-CSA complex
CSA
CSB
RNA polymerase II
Ubiquitin mediated proteolysis
Centrin2
Holo-TFIIH complex
CDK7
XPB
TFIIH1
MNAT1
XPD
TFIIH2
CCNH
TTDA
TFIIH3
TFIIH4
XPG
XPA
RPA
TFIIH complex
XPF
ERCC1
polδ
PCNA
polε
RFC
DNA polymerase δ/ε
Lig1
Lig I
Ubiquitin ligase
Helicase ATPase
TFIIH stabilization
CAK subcomplex
TFIIH core complex
Data on KEGG graph
Rendered by Pathview

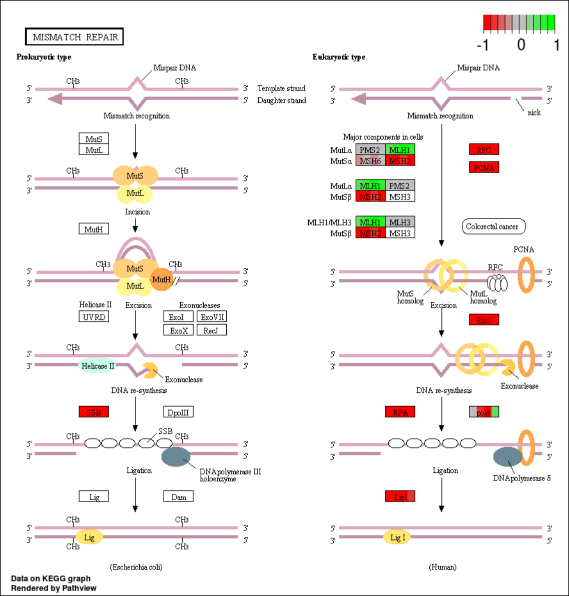

MISMATCH REPAIR
-1
0
1
Prokaryotic type
Mispair DNA
CH3
5'
3'
Template strand
Daughter strand
Mismatch recognition
MutS
MutL
Incision
MutH
Helicase II
UVRD
Excision
Exonucleases
ExoI
ExoVII
ExoX
RecJ
Exonuclease
DNA re-synthesis
SSB
DpoIII
DNApolymerase III holoenzyme
Ligation
Lig
Dam
(Escherichia coli)
Eukaryotic type
nick
Major components in cells
MutLα
MutSα
PMS2
MLH1
MSH6
MSH2
MutSβ
MSH3
MLH1/MLH3
MLH3
RFC
PCNA
Colorectal cancer
MutS homolog
MutL homolog
ExoI
RPA
polδ
DNApolymerase δ
LigI
Lig I
(Human)
Data on KEGG graph
Rendered by Pathview

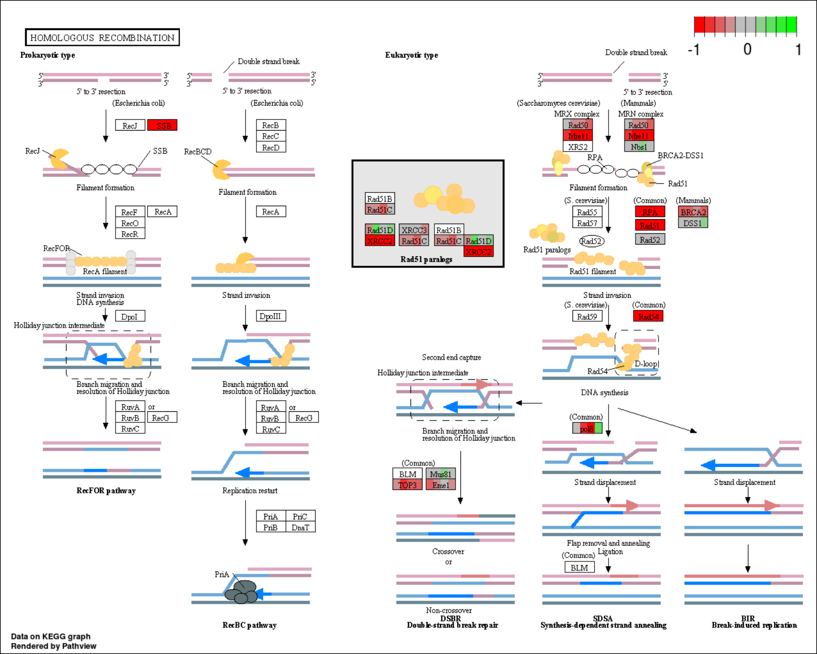
HOMOLOGOUS RECOMBINATION
Prokaryotic type
Eukaryotic type
Double strand break
5' to 3' resection
(Escherichia coli)
RecJ
SSB
RecB
RecC
RecD
RecBCD
Filament formation
RecF
RecO
RecR
RecA
RecFOR
RecA filament
Strand invasion
DNA synthesis
DpoI
DpoIII
Holliday junction intermediate
Branch migration and resolution of Holliday junction
RuvA
RuvB
RuvC
or
RecG
RecFOR pathway
Replication restart
PriA
PriB
PriC
DnaT
RecBC pathway
Rad51B
Rad51C
Rad51D
XRCC2
XRCC3
Rad51 paralogs
(Saccharomyces cerevisiae)
(Mammals)
MRX complex
MRN complex
Rad50
Mre11
XRS2
Nbs1
RPA
BRCA2-DSS1
Rad51
(S. cerevisiae)
(Common)
Rad55
Rad57
Rad52
BRCA2
DSS1
Rad51 filament
Rad59
Rad54
D-loop
Second end capture
DNA synthesis
polδ
BLM
TOP3
Mus81
Eme1
Crossover
or
Non-crossover
Strand displacement
Flap removal and annealing
Ligation
DSBR
Double-strand break repair
SDSA
Synthesis-dependent strand annealing
BIR
Break-induced replication
-1
0
1
Data on KEGG graph
Rendered by Pathview

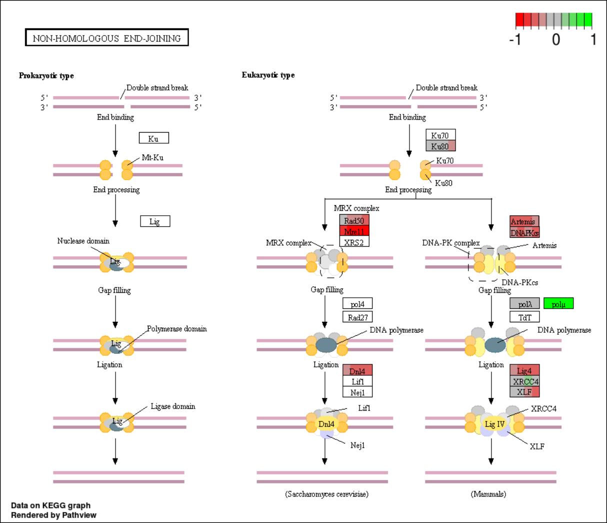

NON-HOMOLOGOUS END-JOINING
-1
0
1
Prokaryotic type
Double strand break
5'
3'
3'
5'
End binding
Ku
Mt-Ku
End processing
Lig
Nuclease domain
Lig
Gap filling
Polymerase domain
Lig
Ligation
Ligase domain
Lig
Eukaryotic type
Double strand break
5'
3'
3'
5'
End binding
Ku70
Ku80
Ku70
Ku80
End processing
MRX complex
Rad50
Mre11
XRS2
MRX complex
Artemis
DNAPKcs
DNA-PK complex
Artemis
DNA-PKcs
Gap filling
pol4
Rad27
DNA polymerase
Gap filling
polλ
polμ
TdT
DNA polymerase
Ligation
Dnl4
Lif1
Nej1
Lif1
Dnl4
Nej1
Ligation
Lig4
XRCC4
XLF
XRCC4
Lig IV
XLF
(Saccharomyces cerevisiae)
(Mammals)
Data on KEGG graph
Rendered by Pathview

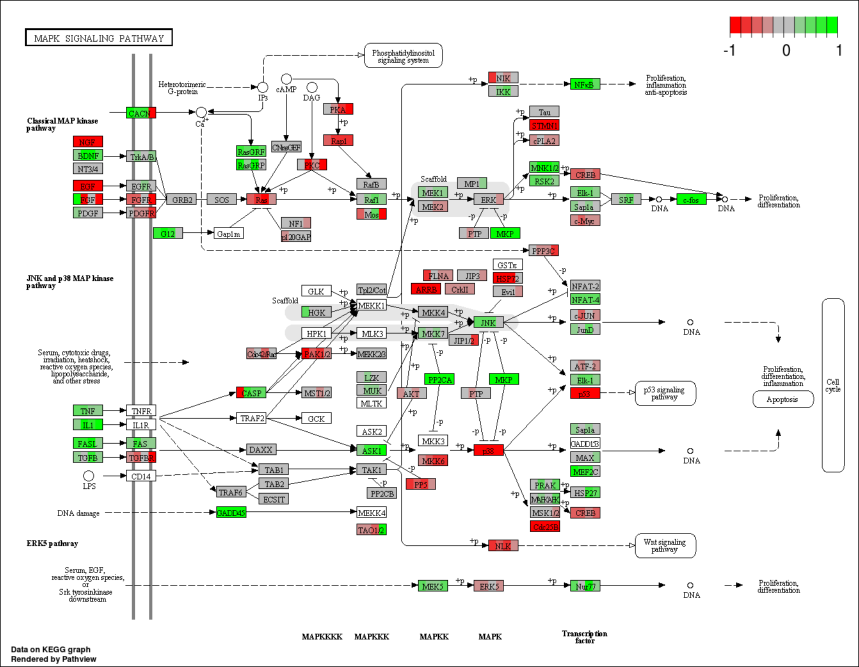
MAPK SIGNALING PATHWAY
-1
0
1
Classical MAP kinase pathway
JNK and p38 MAP kinase pathway
ERK5 pathway
Heterotrimeric G-protein
Phosphatidylinositol signaling system
IP3
cAMP
DAG
Ca2+
CACN
PKA
Rap1
RasGRF
RasGRP
CNrasGEF
PKC
NGF
BDNF
NT3/4
TrkA/B
EGF
FGF
PDGF
EGFR
FGFR
PDGFR
GRB2
SOS
Ras
RafB
Raf1
Mos
NF1
p120GAP
G12
Gap1m
Scaffold
MEK1
MEK2
MP1
ERK
PTP
MKP
NIK
IKK
NFκB
Proliferation, inflammation anti-apoptosis
Tau
STMN1
cPLA2
MNK1/2
RSK2
CREB
Elk-1
Sap1a
c-Myc
SRF
c-fos
DNA
Proliferation, differentiation
PPP3C
NFAT-2
NFAT-4
GSTπ
HSP72
Evi1
FLNA
JIP3
ARRB
CrkII
GLK
Tpl2/Cot
MEKK1
HGK
HPK1
MLK3
MKK4
MKK7
JNK
JIP1/2
c-JUN
JunD
Cdc42/Rac
PAK1/2
MEKK2/3
Serum, cytotoxic drugs, irradiation, heatshock, reactive oxygen species, lipopolysaccharide, and other stress
CASP
MST1/2
LZK
MUK
MLTK
AKT
PP2CA
PTP
MKP
ATF-2
Elk-1
p53
p53 signaling pathway
Proliferation, differentiation, inflammation
Apoptosis
Cell cycle
TNF
IL1
FASL
TGFB
LPS
TNFR
IL1R
FAS
TGFBR
CD14
TRAF2
GCK
DAXX
ASK2
ASK1
MKK3
MKK6
p38
TAB1
TAB2
TAK1
TRAF6
ECSIT
PP2CB
PP5
Sap1a
GADD153
MAX
MEF2C
PRAK
MAPKAPK
MSK1/2
HSP27
CREB
Cdc25B
DNA damage
GADD45
MEKK4
TAO1/2
NLK
Wnt signaling pathway
Serum, EGF, reactive oxygen species, or Srk tyrosinkinase downstream
MEK5
ERK5
Nur77
MAPKKKK
MAPKKK
MAPKK
MAPK
Transcription factor
Data on KEGG graph
Rendered by Pathview

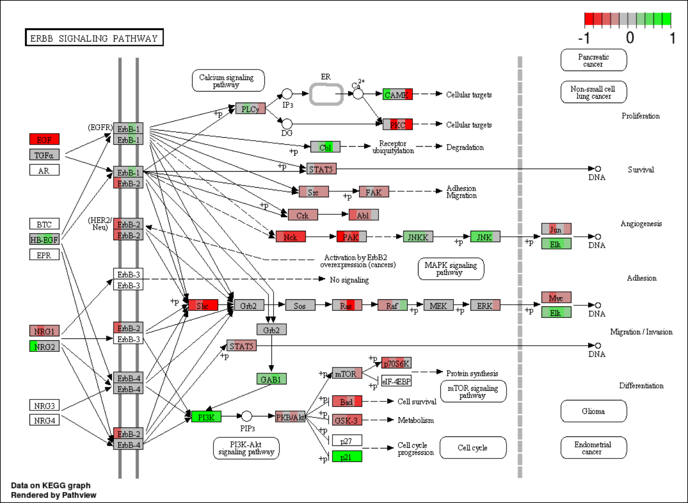
ERBB SIGNALING PATHWAY
-1
0
1
Pancreatic cancer
Non-small cell lung cancer
Proliferation
Survival
Angiogenesis
Adhesion
Migration / Invasion
Differentiation
Glioma
Endometrial cancer
EGF
TGFα
AR
BTC
HB-EGF
EPR
NRG1
NRG2
NRG3
NRG4
(EGFR)
ErbB-1
ErbB-1
ErbB-1
ErbB-2
(HER2/ Neu)
ErbB-2
ErbB-2
ErbB-3
ErbB-3
ErbB-2
ErbB-3
ErbB-4
ErbB-4
ErbB-2
ErbB-4
Calcium signaling pathway
ER
IP3
DG
Ca2+
PLCγ
CAMK
PKC
Cellular targets
Cellular targets
Cbl
Receptor ubiquitylation
Degradation
STAT5
DNA
Src
FAK
Adhesion Migration
Crk
Abl
Nck
PAK
JNKK
JNK
Jun
Elk
DNA
Activation by ErbB2 overexpression (cancers)
MAPK signaling pathway
No signaling
Shc
Grb2
Sos
Ras
Raf
MEK
ERK
Myc
Elk
DNA
Grb2
STAT5
DNA
GAB1
mTOR
p70S6K
eIF-4EBP
Protein synthesis
mTOR signaling pathway
PI3K
PIP3
PKB/Akt
Bad
Cell survival
GSK-3
Metabolism
p27
p21
Cell cycle progression
Cell cycle
PI3K-Akt signaling pathway
+p
Data on KEGG graph
Rendered by Pathview

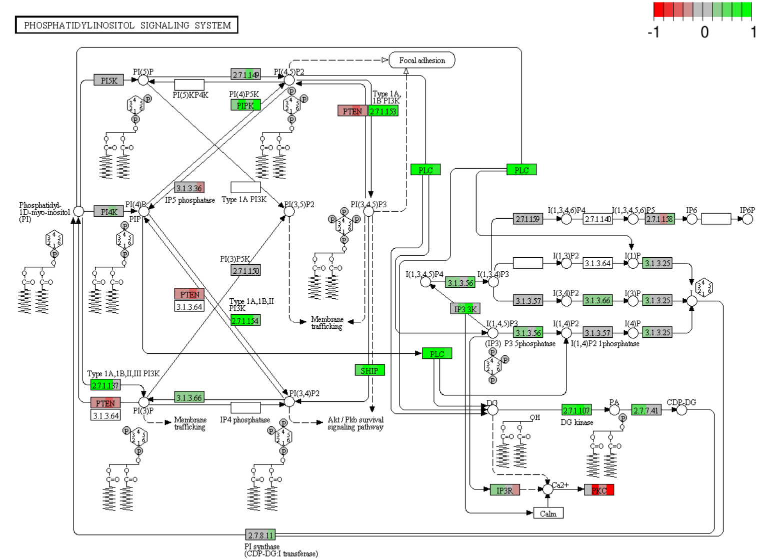

Data on KEGG graph
Rendered by Pathview

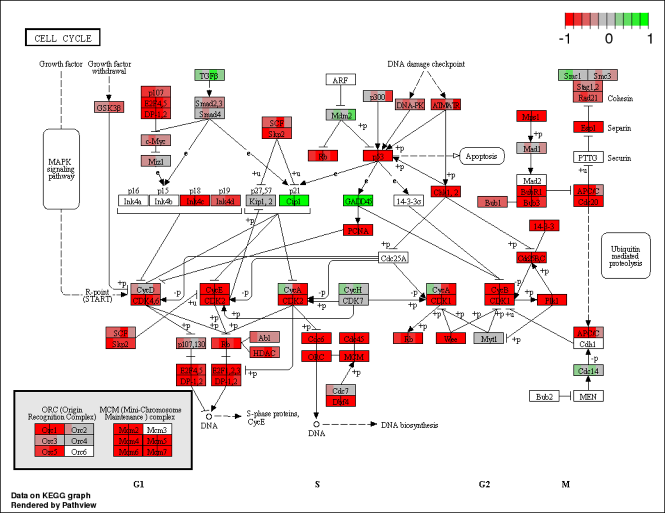
CELL CYCLE
-1
0
1
Growth factor
Growth factor withdrawal
GSK3β
MAPK signaling pathway
R-point (START)
p107
E2F4,5
DP-1,2
TGFβ
Smad2,3
Smad4
c-Myc
Miz1
SCF
Skp2
ARF
Mdm2
p300
DNA damage checkpoint
DNA-PK
ATM/ATR
Rb
p53
Apoptosis
p16
Ink4a
p15
Ink4b
p18
Ink4c
p19
Ink4d
p27,57
Kip1, 2
p21
Cip1
GADD45
14-3-3σ
Chk1, 2
PCNA
Cdc25A
CycD
CDK4,6
CycE
CDK2
CycA
CDK2
CycH
CDK7
CycA
CDK1
CycB
CDK1
Plk1
p107,130
E2F4,5
DP-1,2
Rb
E2F1,2,3
DP-1,2
Abl
HDAC
Cdc6
Cdc45
ORC
MCM
Cdc7
Dbf4
Rb
Wee
Myt1
Smc1
Smc3
Stag1,2
Rad21
Cohesin
Espl
Separin
PTTG
Securin
Mps1
Mad1
Mad2
BubR1
Bub3
Bub1
APC/C
Cdc20
14-3-3
Cdc25B,C
Ubiquitin mediated proteolysis
APC/C
Cdh1
Cdc14
Bub2
MEN
ORC (Origin Recognition Complex)
Orc1
Orc2
Orc3
Orc4
Orc5
Orc6
MCM (Mini-Chromosome Maintenance) complex
Mcm2
Mcm3
Mcm4
Mcm5
Mcm6
Mcm7
DNA
S-phase proteins, CycE
DNA
DNA biosynthesis
G1
S
G2
M
Data on KEGG graph
Rendered by Pathview

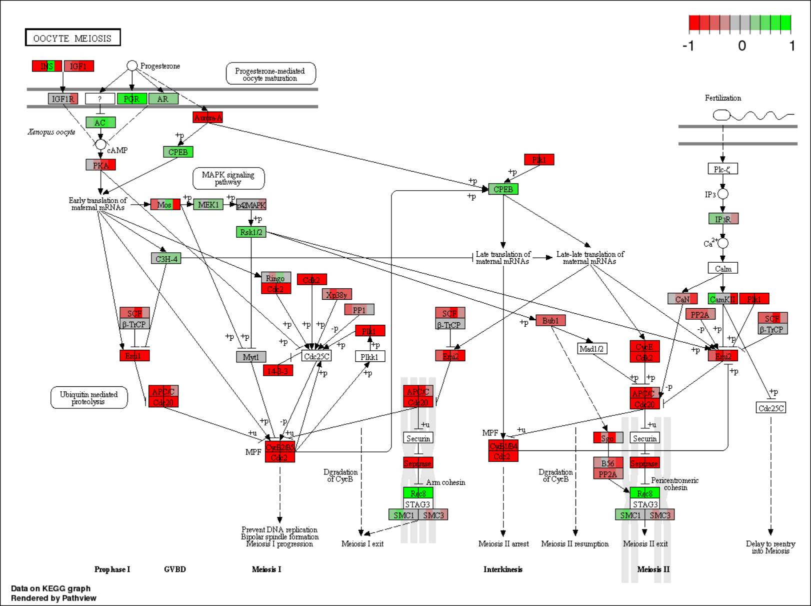
OOCYTE MEIOSIS
-1
0
1
INS
IGF1
Progesterone
Progesterone-mediated oocyte maturation
IGF1R
?
PGR
AR
AC
Aurora-A
Xenopus oocyte
cAMP
+p
CPEB
PKA
MAPK signaling pathway
Plk1
Early translation of maternal mRNAs
Mos
MEK1
p42MAPK
Rsk1/2
C3H-4
Late translation of maternal mRNAs
Late-late translation of maternal mRNAs
Fertilization
Plc-ζ
IP3
IP3R
Ca2+
Calm
CaN
CamKII
PP2A
Ringo
Cdc2
Cdk2
Xp38y
PP1
SCF
β-TrCP
Bub1
Mad1/2
CycE
Emi1
Emi2
Myt1
Cdc25C
Plkk1
14-3-3
Ubiquitin mediated proteolysis
APC/C
Cdc20
Securin
Separase
Arm cohesin
Rec8
STAG3
SMC1
SMC3
MPF
CycB2/B5
CycB1/B4
Sgo
B56
Pericentromeric cohesin
Dgradation of CycB
Prevent DNA replication
Bipolar spindle formation
Meiosis I progression
Meiosis I exit
Meiosis II arrest
Meiosis II resumption
Meiosis II exit
Delay to reentry into Meiosis
Prophase I
GVBD
Meiosis I
Interkinesis
Meiosis II
Data on KEGG graph
Rendered by Pathview

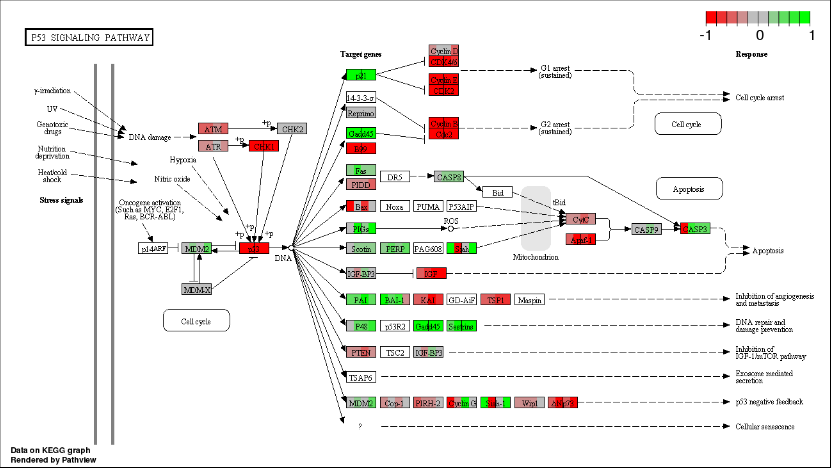
P53 SIGNALING PATHWAY
-1
0
1
Target genes
Response
γ-irradiation
UV
Genotoxic drugs
Nutrition deprivation
Heat/cold shock
Stress signals
DNA damage
ATM
ATR
CHK2
CHK1
Hypoxia
Nitric oxide
Oncogene activation (Such as MYC, E2F1, Ras, BCR-ABL)
p14ARF
MDM2
MDM-X
p53
DNA
Cell cycle
p21
14-3-3-σ
Reprimo
Gadd45
B99
Cyclin D
CDK4/6
Cyclin E
CDK2
Cyclin B
Cdc2
G1 arrest (sustained)
G2 arrest (sustained)
Cell cycle arrest
Cell cycle
Fas
PIDD
DR5
CASP8
Bid
tBid
Bax
Noxa
PUMA
P53AIP
ROS
PIGs
CytC
Apaf-1
CASP9
CASP3
Apoptosis
Apoptosis
Scotin
PERP
PAG608
Siah
Mitochondrion
IGF-BP3
IGF
PAI
BAI-1
KAI
GD-AiF
TSP1
Maspin
Inhibition of angiogenesis and metastasis
P48
p53R2
Gadd45
Sestrins
DNA repair and damage prevention
PTEN
TSC2
IGF-BP3
Inhibition of IGF-1/mTOR pathway
TSAP6
Exosome mediated secretion
MDM2
Cop-1
PIRH-2
Cyclin G
Siah-1
Wip1
ΔNp73
p53 negative feedback
?
Cellular senescence
Data on KEGG graph
Rendered by Pathview

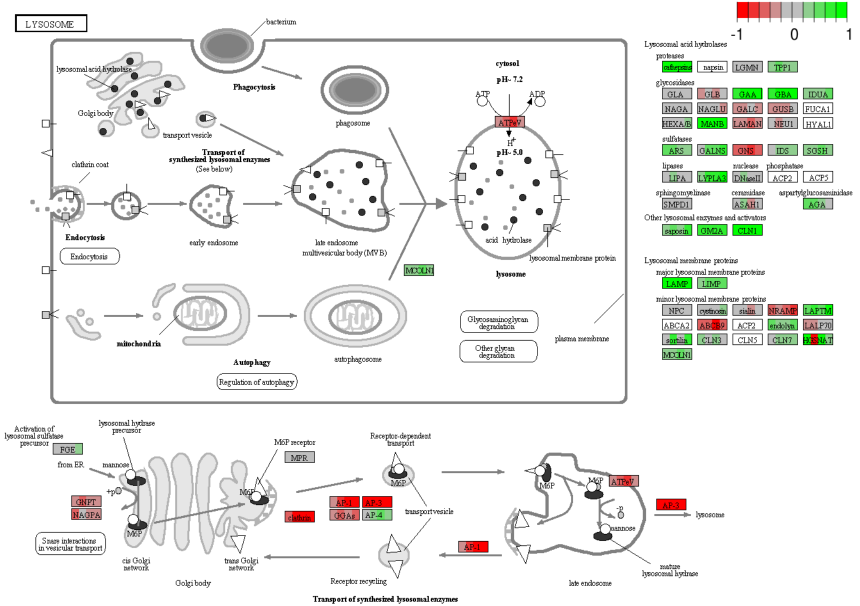

Data on KEGG graph
Rendered by Pathview

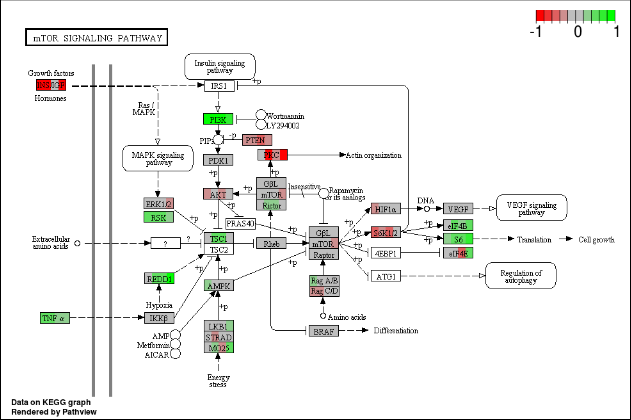

-1 0 1
mTOR SIGNALING PATHWAY
Growth factors
INS/IGF
Hormones
Insulin signaling pathway
IRS1
+p
Ras / MAPK
PI3K
Wortmannin
LY294002
PIP3
-p
PTEN
PKC
Actin organization
MAPK signaling pathway
PDK1
+p
GβL
mTOR
Rictor
Insensitive
Rapamycin or its analogs
AKT
ERK1/2
RSK
PRAS40
HIF1α
DNA
VEGF
VEGF signaling pathway
S6K1/2
eIF4B
S6
Translation
Cell growth
4EBP1
eIF4E
Extracellular amino acids
?
TSC1
TSC2
Rheb
Raptor
ATG1
Regulation of autophagy
REDD1
AMPK
Rag A/B
Rag C/D
Hypoxia
Amino acids
TNF α
IKKβ
BRAF
Differentiation
AMP
Metformin
AICAR
LKB1
STRAD
MO25
Energy stress
Data on KEGG graph
Rendered by Pathview

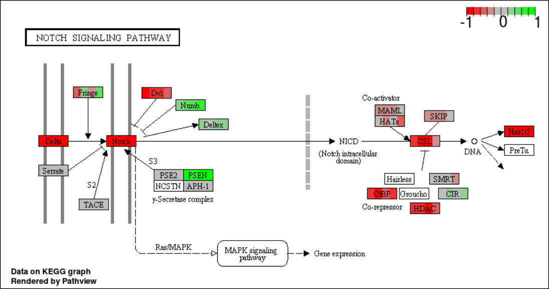

-1
0
1
NOTCH SIGNALING PATHWAY
Fringe
Dvl
Numb
Deltex
Delta
Notch
Serrate
S2
TACE
S3
PSE2
PSEN
NCSTN
APH-1
γ-Secretase complex
NICD
(Notch intracellular domain)
Co-activator
MAML
HATs
SKIP
CSL
DNA
Hes1/5
PreTα
Hairless
SMRT
CtBP
Groucho
CIR
Co-repressor
HDAC
Ras/MAPK
MAPK signaling pathway
Gene expression
Data on KEGG graph
Rendered by Pathview

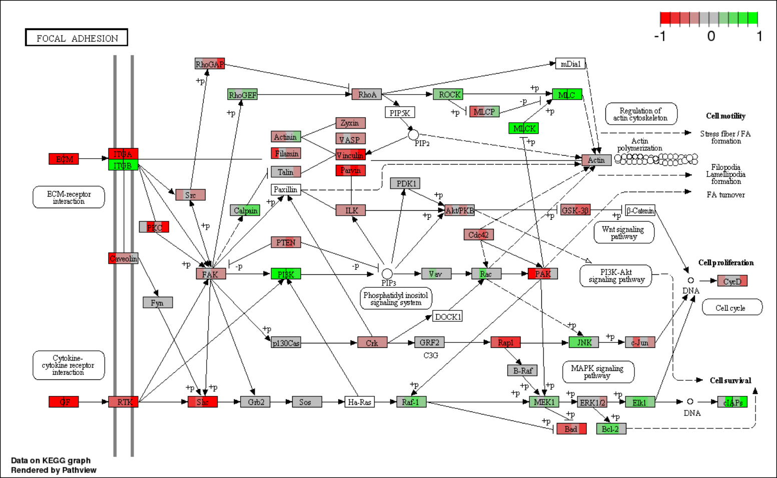

FOCAL ADHESION
-1
0
1
RhoGAP
mDia1
RhoGEF
RhoA
ROCK
MLC
MLCP
PIP5K
MLCK
Regulation of actin cytoskeleton
Cell motility
Zyxin
Actinin
VASP
PIP2
Stress fiber / FA formation
Actin polymerization
ECM
ITGA
ITGB
Filamin
Vinculin
Talin
Actin
Parvin
Filopodia Lamellipodia formation
ECM-receptor interaction
Src
Paxillin
PDK1
FA turnover
Calpain
ILK
Akt/PKB
GSK-3β
β-Catenin
PKC
Cdc42
Wnt signaling pathway
PTEN
Caveolin
FAK
PI3K
PIP3
Vav
Rac
PAK
PI3K-Akt signaling pathway
Cell proliferation
DNA
CycD
Fyn
Phosphatidyl inositol signaling system
DOCK1
Cell cycle
p130Cas
Crk
GRF2
C3G
Rap1
JNK
c-Jun
Cytokine-cytokine receptor interaction
B-Raf
MAPK signaling pathway
Cell survival
GF
RTK
Shc
Grb2
Sos
Ha-Ras
Raf-1
MEK1
ERK1/2
Elk1
DNA
cIAPs
Bad
Bcl-2
Data on KEGG graph
Rendered by Pathview

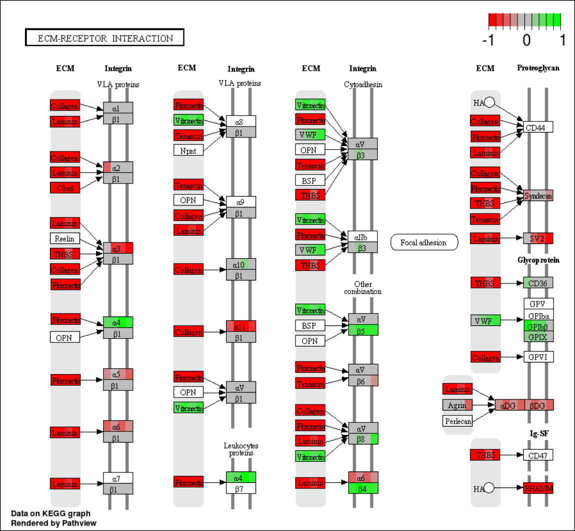
ECM-RECEPTOR INTERACTION
-1 0 1
ECM
Integrin
VLA proteins
Collagen
Laminin
α1
β1
α2
Chad
Reelin
THBS
Fibronectin
α3
α4
OPN
α5
α6
α7
α8
Vitronectin
Tenascin
Npnt
α9
α10
α11
αV
Leukocytes proteins
β7
Cytoadhesin
VWF
BSP
β3
αIIb
Other combination
β5
β6
β8
β4
Focal adhesion
Proteoglycan
HA
CD44
Syndecan
SV2
Glycoprotein
CD36
GPV
GPIbα
GPIbβ
GPIX
GPVI
Agrin
Perlecan
αDG
βDG
Ig-SF
CD47
RHAMM
Data on KEGG graph
Rendered by Pathview

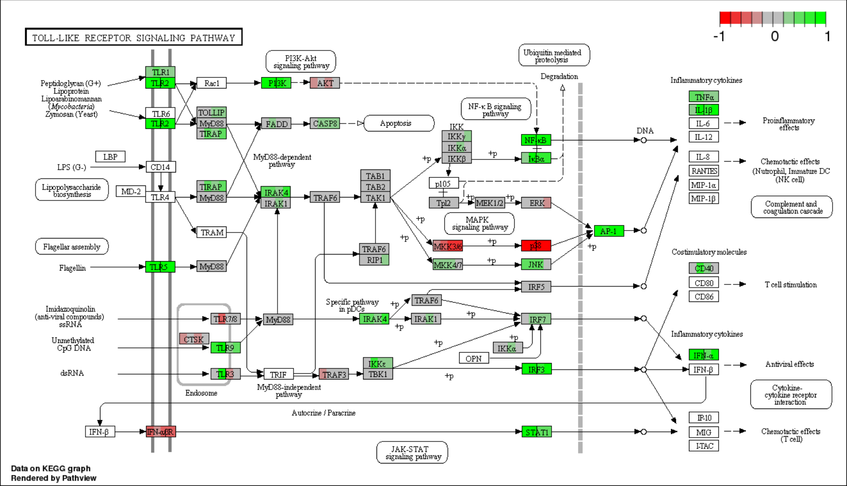
TOLL-LIKE RECEPTOR SIGNALING PATHWAY
-1
0
1
Peptidoglycan (G+)
Lipoprotein
Lipoarabinomannan
(Mycobacteria)
Zymosan (Yeast)
TLR1
TLR2
TLR6
TLR2
Rac1
PI3K
AKT
PI3K-Akt signaling pathway
TOLLIP
MyD88
TIRAP
FADD
CASP8
Apoptosis
Ubiquitin mediated proteolysis
Degradation
NF-κB signaling pathway
IKK
IKKγ
IKKα
IKKβ
NF-κB
IκBα
DNA
Inflammatory cytokines
TNFα
IL-1β
IL-6
IL-12
Proinflammatory effects
IL-8
RANTES
MIP-1α
MIP-1β
Chemotactic effects
(Nutrophil, Immature DC (NK cell)
Complement and coagulation cascade
LBP
LPS (G-)
CD14
Lipopolysaccharide biosynthesis
MD-2
TLR4
TIRAP
MyD88
IRAK4
IRAK1
TRAF6
TAB1
TAB2
TAK1
MyD88-dependent pathway
p105
Tpl2
MEK1/2
ERK
MAPK signaling pathway
AP-1
TRAM
Flagellar assembly
TRAF6
RIP1
MKK3/6
p38
MKK4/7
JNK
Costimulatory molecules
CD40
CD80
CD86
T cell stimulation
Flagellin
TLR5
MyD88
IRF5
Imidazoquinolin
(anti-viral compounds)
ssRNA
TLR7/8
MyD88
Specific pathway in pDCs
IRAK4
TRAF6
IRAK1
IRF7
Unmethylated CpG DNA
CTSK
TLR9
IKKα
OPN
Inflammatory cytokines
IFN-α
IFN-β
Antiviral effects
dsRNA
TLR3
TRIF
TRAF3
IKKε
TBK1
IRF3
Endosome
MyD88-independent pathway
Cytokine-cytokine receptor interaction
Autocrine / Paracrine
IFN-β
IFN-αβR
STAT1
IP-10
MIG
I-TAC
Chemotactic effects
(T cell)
JAK-STAT signaling pathway
+p
Data on KEGG graph
Rendered by Pathview

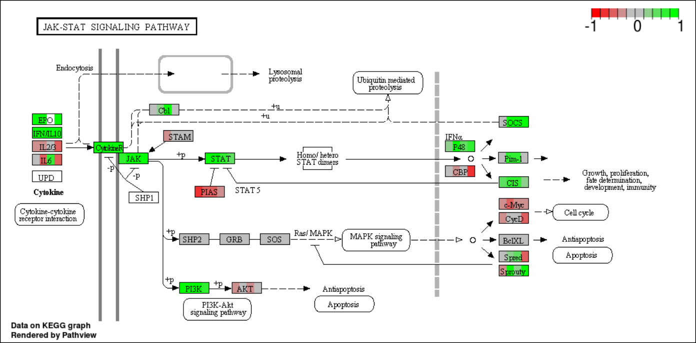

JAK-STAT SIGNALING PATHWAY
-1
0
1
Endocytosis
Lysosomal proteolysis
Ubiquitin mediated proteolysis
Cbl
+u
+u
SOCS
EPO
IFN/IL10
IL2/3
IL6
UPD
Cytokine
CytokineR
STAM
JAK
+p
STAT
Homo/ hetero STAT dimers
IFNα
P48
CBP
Pim-1
CIS
Growth, proliferation, fate determination, development, immunity
-p
-p
SHP1
PIAS
STAT 5
Cytokine-cytokine receptor interaction
c-Myc
CycD
Cell cycle
+p
SHP2
GRB
SOS
Ras/ MAPK
MAPK signaling pathway
BclXL
Antiapoptosis
Apoptosis
Spred
Sprouty
+p
PI3K
+p
AKT
Antiapoptosis
Apoptosis
PI3K-Akt signaling pathway
Data on KEGG graph
Rendered by Pathview

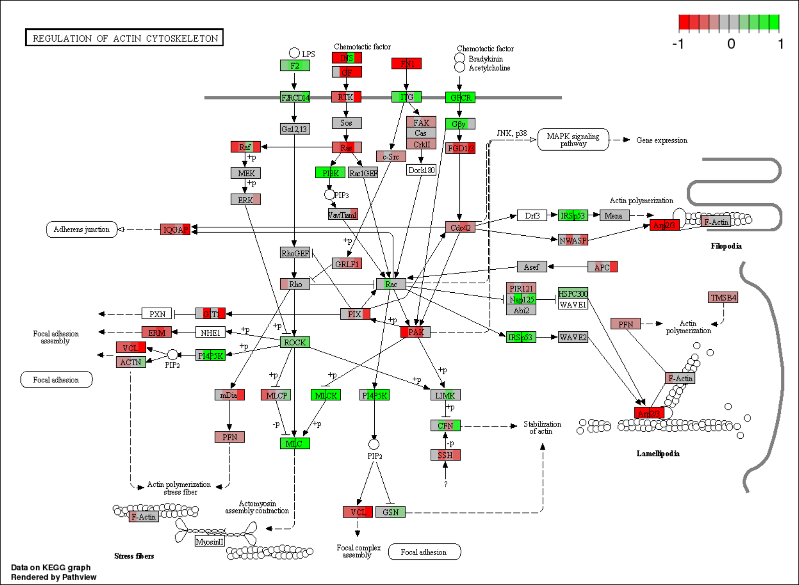

REGULATION OF ACTIN CYTOSKELETON
-1
0
1
LPS
F2
F2R/CD14
Gα12,13
Chemotactic factor
INS
GF
RTK
Sos
Ras
Raf
+p
MEK
+p
ERK
FN1
ITG
FAK
Cas
CrkII
Dock180
c-Src
PI3K
Rac1GEF
PIP3
Vav/Tiam1
Chemotactic factor
Bradykinin
Acetylcholine
GPCR
Gβγ
FGD1/3
JNK, p38
MAPK signaling pathway
Gene expression
Adherens junction
IQGAP
Cdc42
Drf3
IRSp53
Mena
Actin polymerization
Arp2/3
F-Actin
Filopodia
NWASP
RhoGEF
GRLF1
+p
Rho
Rac
Asef
APC
PIR121
Nap125
Abi2
HSPC300
WAVE1
TMSB4
PFN
Actin polymerization
PXN
GIT1
PIX
+p
PAK
IRSp53
WAVE2
Focal adhesion assembly
ERM
NHE1
+p
+p
ROCK
VCL
ACTN
PIP2
PI4P5K
+p
Focal adhesion
F-Actin
+p
+p
+p
+p
mDia
MLCP
MLCK
PI4P5K
LIMK
+p
Arp2/3
Lamellipodia
-p
+p
CFN
Stabilization of actin
PFN
MLC
PIP2
-p
SSH
?
Actin polymerization stress fiber
Actomyosin assembly contraction
VCL
GSN
F-Actin
MyosinII
Stress fibers
Focal complex assembly
Focal adhesion
Data on KEGG graph
Rendered by Pathview

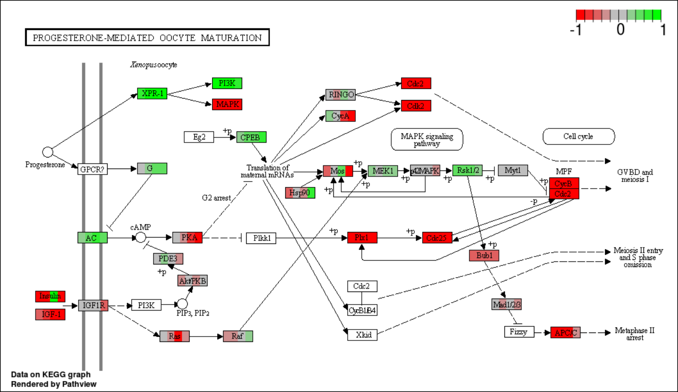
PROGESTERONE-MEDIATED OOCYTE MATURATION
-1
0
1
Xenopus oocyte
XPR-1
PI3K
MAPK
Progesterone
GPCR?
G
AC
cAMP
PKA
PDE3
Akt/PKB
Insulin
IGF-1
IGF1R
PI3K
PIP3, PIP2
Ras
Raf
Eg2
CPEB
Translation of maternal mRNAs
G2 arrest
RINGO
CycA
Cdc2
Cdk2
Mos
Hsp90
MEK1
p42MAPK
Rsk1/2
Myt1
MPF
CycB
Cdc2
MAPK signaling pathway
Cell cycle
GVBD and meiosis I
Plkk1
Plx1
Cdc25
Bub1
Mad1/2/3
Fizzy
APC/C
Metaphase II arrest
Meiosis II entry and S phase omission
Cdc2
CycB1/B4
Xkid
Data on KEGG graph
Rendered by Pathview
