# Supplementary material for: RNA-seq of the aging brain in the short-lived fish N. furzeri – conserved pathways and novel genes associated with neurogenesis
Source: Aging Cell. 2014 Jul 25;13(6):965–74. doi: 10.1111/acel.12257 (PMC4326923; doi:10.1111/acel.12257)
Supplement: Supplementary file 12 [file acel0013-0965-sd12.docx]

**MATERIALS & METHODS**

Extraction of Total RNA from *N. furzeri*

For expression profiling whole brains from male animals of *N. furzeri* (strain MZM-04/10) were collected from different ages: at 5, 12, 20, 27, and 39 weeks of age. To avoid effects of circadian rhythms and feeding, animals were always sacrificed at 10 a.m. in fasted state. For tissue preparation, fish were euthanized with MS-222 and cooled on crushed ice. The protocols of animal maintenance and experiments were approved by the local authority in the State of Thuringia (Veterinaer- und Lebensmittelueberwachungsamt). The whole fish brains were dissected and transferred into 2 ml tubes with 1 ml cooled QIAzol (Qiagen, Hilden, Germany) and one 5 mm stainless steel bead (Qiagen) was added. Homogenization was performed using a TissueLyzer II (Qiagen) at 20 Hz for 3x 1 min. After incubation for 5 min at room temperature 200 µl chloroform was added. The tube was shaken for 15 s and incubated for 3 min at room temperature. Phase separation was achieved by centrifugation at 12,000x g for 20 min at 4°C. The aqueous phase was transferred into a fresh cup and 10 µg of Glycogen (Invitrogen, Darmstadt, Germany), 0.16x volume NaAc (2 M; pH 4.0) and 1.1x volume isopropanol were added, mixed and incubated for 10 min at room temperature. The RNA was precipitated by a centrifugation step with 12,000x g at 4°C for 20 min. The supernatant was removed and the pellet was washed with 80% Ethanol twice and air dried for 10 min. The RNA was resuspended in 20 µl DEPC-treated water by pipetting up and down, followed by incubation at 65°C for 5 min. The RNA was quantified with a NanoDrop 1000 (PeqLab, Erlangen, Germany) and stored at -80°C until use.

RNA-seq

Sequencing procedure was done using Illumina methodology ([Bentley *et al.* 2008](#_ENREF_3)). Around 2.5 µg of total RNA was used for library preparation (Illumina, TruSeq™ RNA Sample Prep Kit v2) using the manufacturer’s description. Libraries were sequenced using a HiSeq2000 (Illumina) running in 50 bp single-read mode using sequencing chemistry v2. This resulted in around 29-54 million reads per sample with 50 bp length. Reads were extracted in FASTQ format using CASAVA v.1.8 (Illumina). FASTQ formatted reads were used for following analysis. Read mapping was performed using Bowtie ([Langmead](#_ENREF_9) *[et al.](#_ENREF_9)* [2009](#_ENREF_9)) versus a transcriptome catalogue of *N. furzeri* containing 19,812 transcript contigs ([Petzold](#_ENREF_11) *[et al.](#_ENREF_11)* [2013](#_ENREF_11)). On average, 52% of all reads could be mapped uniquely and were used for counting afterwards. The transcripts were annotated additionally using Ensembl gene identifiers from *D. rerio*, while 13,561 shared a best bi-directional BLAST hit between both species ([Petzold](#_ENREF_11) *[et al.](#_ENREF_11)* [2013](#_ENREF_11)). All 19,812 transcripts were used for further analysis. Since there is currently no gene-enrichment tool available for *N.  furzeri* specific gene identifier, official gene names of the best BLAST hit were used to annotate *N. furzeri* transcript contigs ([Petzold](#_ENREF_11) *[et al.](#_ENREF_11)* [2013](#_ENREF_11)). The read counts were normalized with respect to the size of the individual transcript contigs and to the total amount of mappable reads obtained for the particular sample, resulting in reads per kilobase and million mapped reads (RPKM) values ([Mortazavi](#_ENREF_10) *[et al.](#_ENREF_10)* [2008](#_ENREF_10)) for each transcript contig.

DEG identification and clustering

Differentially expressed genes (DEGs) were identified by applying the statistical tests of the edgeR ([Robinson *et al.* 2010](#_ENREF_12)), DESeq ([Anders & Huber 2010](#_ENREF_1)) and baySeq ([Hardcastle & Kelly 2010](#_ENREF_7)) packages, while DEGs were selected according a FDR < 0.05 by all three tests.

The RPKM counts were clustered in order to identify common expression profiles. Therefore, the fuzzy c-means (FCM) clustering algorithm ([Bezdek & Hathaway 1992](#_ENREF_4)) was applied. The optimal number of clusters was estimated by the vote of several cluster validity indices, which capture different aspects of a clustering structure ([Guthke](#_ENREF_6) *[et al.](#_ENREF_6)* [2005](#_ENREF_6)). The FCM algorithm converges to a local optimum – six expression clusters were identified as optimal result.

Identification of orthologs and comparison with metanalysis.

Orthologous genes between N.furzeri and human were identified with the orthology R package (Priebe and Menzel, 2013) by the use of 3 databases (HomoloGene, Ensembl Compara and Inparanoid). Data for comparison were downloaded from http://genomics.senescence.info/gene_expression/signatures_supplement.zip

The human orthologe gene symbols extracted from 4 lists (“Genes_overexpressed”, “Genes_underexpressed”, ”chi_overexpressed”, ”chi_underexpressed“) have been used for comparison with DEG found in N.furzeri. 1655 DEG selected from Cluster 2,4 & 6 (up-regulated with age) were mapped to 1542 orthologous human gene symbols and 2449 DEG from cluster 1,3,5 (down-regulated with age) to 2365 orthologous human gene symbols using the R orthology package (Priebe and Menzel, 2013).

Network analysis

Networks were generated in Cytoscape 3.0.1 ([Shannon *et al.* 2003](#_ENREF_13)) giving as input pairs of genes and their relative Pearson correlation. As layout, spring embedded weighted on correlation and default settings were always used.

For the general DEGs network, correlation was calculated between 25 elements long vectors corresponding to expression values for all samples ordered according to age, generating a matrix 4104x4104. A threshold of r > 0.95 was set.

For the age-specific networks, Pearson’s correlation was similarly calculated, but with vectors of five elements corresponding to expression values for all individuals sacrificed at the same age. A threshold of r ≥ 0.99 was set and for each time point a network was produced. Nodes were colored using VizMapper according to their cluster membership (discrete mapping, rainbow colors).

ClueGO analysis

Gene sets of each cluster were imported in Cytoscape and then used for Gene Ontology analysis with ClueGO plug-in ([Bindea](#_ENREF_5) *[et al.](#_ENREF_5)* [2009](#_ENREF_5)). The attribute used to import the network in the tool was “name” and the comparison was made with *Homo sapiens* identifiers. Gene Ontology data were updated on the 9/19/2013 and then used for the whole set of analysis. As statistical test “Enrichment (Right-sided hypergeometric test)” was used, without correction for multiple testing and a p-value threshold of 0.05. Moreover, the options “Use GO Term Fusion” and “Use GO Term Grouping” were selected. The same settings were used to analyze connected components in the 39 weeks network: each connected component was selected and imported in ClueGO to perform a comparative analysis of GO terms specifically enriched in either of the two networks.

KEGG pathway maps

The pathview R package (Luo and Brouwer, 2013) was used to visualize fold-changes for KEGG pathways found as differentially regulated. The mean RPKM values have been used for calculation of fold-changes (12w/5w, 20w/5w, 27w/5w, 39w/5w) which were included in the pathview maps using 4 different colors in each node. D.rerio was used as reference species. The corresponding genes were mapped to D.rerio Ensembl gene ids using the orthology R package (Priebe and Menzel, 2013) and used as input for pathview.

Quantitative real-time PCR

Real-time PCR was performed with the CFX384 (Biorad) and the Quantitect PCR system (Qiagen). Steps were processed as recommended by the manufacturer. In brief, we used 500 ng total RNA for cDNA sysnthesis in a 20 µl Volume. After cDNA synthesis samples were diluted to a final volume of 200 µl with ultra-pure water. PCR reactions were performed in 10 µl volume with 1 µl diluted cDNA using the Quantitect SYBR Green PCR kit (Qiagen). Forward and reverse primers were always located in two different exons (primer sequences can be found in Table S2). A cDNA pool was serially diluted (from 80 to 2.5 ng per reaction) and used to create standard as well as melting curves and to calculate amplification efficiencies for each primer pair prior use for quantification. All reactions were performed in triplicates and negative (water) as well as genomic (without reverse transcriptase) controls were always included. Fold changes describe the difference in expression level between young and old age animals normalised to TATA box binding protein (TBP). Sequences for TBP primers were kindly provided by Dr. Nils Hartmann. Statistical analysis of real-time data was performed with the relative expression software tool REST (Qiagen, Pfaffl et al., 2002). This software tool uses a mathematical model that compares unknown and control samples and significance is tested by a randomisation test (pair wise fixed reallocation randomisation test).

Zebrafish gene cloning and *in situ* hybridization

Zebrafish embryos were obtained and staged as previously described ([Kimmel 1995](#_ENREF_8)). 72 hpf zebrafish embryos were fixed in 4% paraformaldehyde. *In situ* hybridization (ISH) was then performed according to published protocols ([Thisse & Thisse 2008](#_ENREF_14)). To prepare Dig-labeled antisense RNA probes for ISH, *cbx1a* (NCBI, NM_199746), *dnmt3aa* (NCBI, NM_001018134), *mex3a* (ENSEMBL, ENSDARG00000076638), *scml4* (NCBI, XM_001332397) and cDNA fragments were amplified by PCR from 48 hpf zebrafish embryos total RNA using primer pairs listed in Table S7. The corresponding cDNAs were subcloned into pGEM-T vector (Promega) and DIG-labeled RNA antisense probes were transcribed using standard protocols. The EST clone I.M.A.G.E. 7430154 (Gene Bank, IRB067) was found to correspond to the coding region of the zebrafish *znf367* gene. The coding region of *znf367* was subcloned into the pSK- vector in the EcoRI-EcoRV sites and then used for preparing RNA antisense probe for ISH. Full-length *agr2* and *krcp*c DNAs were PCR-amplified from zebrafish embryo total RNA and directly *in vitro* transcribed. The *pcna* probe was generated as described ([Beetz](#_ENREF_2) *[et al.](#_ENREF_2)* [2007](#_ENREF_2)).

*N. furzeri in situ* hybridization

To generate the probes, RNA was extracted from a pool of 2-3 brains for *N. furzeri* and a pool of 20 zebrafish embryos. Brains and embryos were put in 700 µl Qiazol (QIAzol Lysis Reagent, Qiagen) and RNA was extracted using RNeasy Micro Kit (Qiagen). cDNA synthesis was carried out using Reverse transcriptase Core kit (Eurogentec). PCR was performed on cDNA using GoTaq polymerase (Promega). 0.5 µg of PCR products containing T7 RNA polymerase promoter at the 3' ends were used as templates for *in vitro* transcription. Probes were transcribed using DIG RNA labeling kit (SP6/T7) (Roche) according to the manufacturer's protocol. The sequences of the primers and probes used in this study are reported in Table S8.

*In situ* hybridization was performed on 16 μm thick cryo-sections of fish brain. Slides were dried for 2 h at 37°C, washed in PBS twice for 3 min, and then treated for 8 min with Proteinase K (diluted 1:80000 starting from stocks of 20 mg/ml). Slides were washed in Glycine (2 mg/ml in PBT) twice for 5 min, to stop the reaction. Sections were fixed with 4% PFA for 20 min at room temperature, and washed in PBT (three times for 3 min). Pre-hybridization was performed covering the slides with 200 μl of hybridization buffer under parafilm coverslips (to avoid evaporation) at 60°C hybridization temperature for 30 min. Hybridization was done by covering each slide with a solution of the specific antisense probe in 200 μl of hybridization buffer to a final concentration of 1 μg/ml. Parafilm coverslips were used and slides incubated at hybridization temperature overnight in a humidified chamber. Before use diluted RNA probes were denatured for 5 min at 80°C.

After hybridization, 2x SSC was used to remove the coverslip. Slides were first washed in 1x SSC, twice for 20 min, and then in 0.2x SSC twice for 20 min, always at hybridization temperature. A final washing step was done in PBT three times for 5 min at room temperature.

For probe revelation slides were incubated with blocking solution for 30 min at room temperature and then with Anti-Dig-AP Fab Fragments Ab (Roche; 1:2000) in blocking solution overnight at 4°C.

Washings in PBT, 3 times for 5 min, and in NMNT, 3 times for 5 min at room temperature, was conducted before adding Fast Red solution (Roche Tablets; 1 in 2 ml Tris-HCl 0.1 M, pH = 8.2). To avoid the formation of precipitate, Fast Red tablets were vortexed for 5 min in Tris-HCl and then filtered. Slides were observed every 20 min with the microscope until signal detection (1-10 h depending on the probe used). The staining was stopped by washing in PBS (at least 3 times for 5 min) at room temperature. Then slides were mounted with a specific mounting medium (Fluoroshield, Sigma) and analyzed with a confocal microscope (Leica TCS).

Immunohistochemistry

Sections from brains were deparaffinized and subjected to 95°C for 30 min for antigen retrieval in a solution of 0.01 M sodium citrate buffer (Abcam). Sections were incubated for 1 h with the background reducing solution (DAKO) to block nonspecific protein-binding sites and overnight at 4°C with Histone 3 Lysine 27 trimethylation (H3K27me3) antibody (1:50, monoclonal, Abcam). After a brief rinse, sections were incubated with the Alexa Fluor® 546 secondary antibodies (1:200; Life Technologies). Nuclei were counterstained with 3.3 µM TOPRO3 (Life Technologies) for 20 min at RT. Samples were analyzed using a Leica TCS SP8 Confocal Microscope with 40x magnification. Lasers power, beam splitters, filter settings, pinhole diameters and scan mode were the same for all examined samples of each sample. Fields reported in the figures are representative of all examined fields.

**References for Methods**

Anders S, Huber W (2010). Differential expression analysis for sequence count data. *Genome Biol*. **11**, R106.

Beetz S, Diekhoff D, Steiner LA (2007). Characterization of terminal deoxynucleotidyl transferase and polymerase mu in zebrafish. *Immunogenetics*. **59**, 735-744.

Bentley DR, Balasubramanian S, Swerdlow HP et al. (2008). Accurate whole human genome sequencing using reversible terminator chemistry. *Nature*. **456**, 53-59.

Bezdek JC, Hathaway RJ (1992). Numerical convergence and interpretation of the fuzzy c-shells clustering algorithm. *IEEE transactions on neural networks / a publication of the IEEE Neural Networks Council*. **3**, 787-793.

Bindea G, Mlecnik B, Hackl H, Charoentong P, Tosolini M, Kirilovsky A, Fridman WH, Pages F, Trajanoski Z, Galon J (2009). ClueGO: a Cytoscape plug-in to decipher functionally grouped gene ontology and pathway annotation networks. *Bioinformatics*. **25**, 1091-1093.

Guthke R, Moller U, Hoffmann M, Thies F, Topfer S (2005). Dynamic network reconstruction from gene expression data applied to immune response during bacterial infection. *Bioinformatics*. **21**, 1626-1634.

Hardcastle TJ, Kelly KA (2010). baySeq: empirical Bayesian methods for identifying differential expression in sequence count data. *BMC bioinformatics*. **11**, 422.

Kimmel CB, Ballard W.W., Kimmel, S.R., Ullmann, B., Schilling, T.F. (1995). Stages of embryonic development of the Zebrafish. *Developmental Dynamics*. **203**, 253-310.

Langmead B, Trapnell C, Pop M, Salzberg SL (2009). Ultrafast and memory-efficient alignment of short DNA sequences to the human genome. *Genome Biol*. **10**, R25.

Luo, W and Brouwer C (2013). Pathview: an R/Bioconductor package for pathway-based data integration and visualization. *Bioinformatics*, 29(14), pp. 1830-1831.

Mortazavi A, Williams BA, McCue K, Schaeffer L, Wold B (2008). Mapping and quantifying mammalian transcriptomes by RNA-Seq. *Nature methods*. **5**, 621-628.

Petzold A, Reichwald K, Groth M, Taudien S, Hartmann N, Priebe S, Shagin D, Englert C, Platzer M (2013). The transcript catalogue of the short-lived fish Nothobranchius furzeri provides insights into age-dependent changes of mRNA levels. *BMC Genomics*. **14**, 185.

Priebe, S. and Menzel, U (2013). Assignment of Orthologous Genes by Utilization of Multiple Databases - The Orthology Package in R. BIOINFORMATICS 2013 - International Conference on Bioinformatics Models, Methods and Algorithms. (eds Fernandes, P., Sole-Casals, J., Fred, L. N. A. & Gamboa, H.) 105–110.

Robinson MD, McCarthy DJ, Smyth GK (2010). edgeR: a Bioconductor package for differential expression analysis of digital gene expression data. *Bioinformatics*. **26**, 139-140.

Shannon P, Markiel A, Ozier O, Baliga NS, Wang JT, Ramage D, Amin N, Schwikowski B, Ideker T (2003). Cytoscape: a software environment for integrated models of biomolecular interaction networks. *Genome Res*. **13**, 2498-2504.

Thisse C, Thisse B (2008). High-resolution in situ hybridization to whole-mount zebrafish embryos. *Nat Protoc*. **3**, 59-69.
